# Canonical Notch2 signaling regulates development and maintenance of iron-recycling macrophages and iron homeostasis

**DOI:** 10.1038/s41467-026-77938-7

**Published:** 2026-09-23

**Authors:** Frauline Nicole Schroth, Tamar Kapanadze, Stefan Sablotny, Yuangao Xu, Alessia Ascierto, Matthias Ballmaier, Katharina Mauel, Bo Mee Chung, Lena Deuper, Andrey S. Shaw, Andreas Kispert, Matthias Lochner, Tibor Kempf, Elvira Mass, Hermann Haller, Kai M. Schmidt-Ott, Jaba Gamrekelashvili, Florian P. Limbourg

**Affiliations:** 1 Vascular Medicine Research, Department of Nephrology and Hypertension, Hannover Medical School, Hannover, Germany; 2 Department of Nephrology and Hypertension, Hannover Medical School, Hannover, Germany; 3 MDI Biological Laboratory, Bar Harbor, Maine, USA; 4 Department of Translational Medicine and Laboratory for Technologies of Advanced Therapies (LTTA), University of Ferrara, Ferrara, Italy; 5 Central Research Facility Cell Sorting, Hannover Medical School, Hannover, Germany; 6 Developmental Biology of the Immune System, Life and Medical Sciences (LIMES) Institute, University of Bonn, Bonn, Germany; 7 Department of Cardiology and Angiology, Hannover Medical School, Hannover, Germany; 8 Institute of Molecular Biology, Hannover Medical School, Hannover, Germany; 9 Department of Research Biology, Genentech Research and Early Development, South San Francisco, California, USA; 10 Institute of Medical Microbiology and Hospital Epidemiology, Hannover Medical School, Hannover, Germany; 11 Present Address: Department of Cardiology and Intensive Care Medicine, Städtisches Klinikum Braunschweig, Braunschweig, Germany

**Keywords:** Haematopoiesis, Innate immunity, Monocytes and macrophages, Signal transduction

## Abstract

Red pulp macrophages (RPM) and bone marrow macrophages (BMM) are iron-recycling cells, involved in iron homeostasis and erythropoiesis. Here we show, by conditional deletion strategies using Cx3cr1Cre-mediated targeting to inactivate Notch signalling components in mice, that canonical Notch2 signalling regulates the development of RPM and BMM. Loss of functional Notch2, or its nuclear mediator Rbpj, impair RPM and BMM development and cause iron overload in the spleen and bone marrow. In the absence of Notch2, prototypic RPM genes are downregulated, which is accompanied by splenic extramedullary haematopoiesis and changes in splenic microarchitecture. Treatment of mice with an anti-Notch2 blocking antibody recapitulates the impaired erythrophagocyte and de novo extramedullary hematopoiesis phenotypes characteristic to the genetic model. Furthermore, early postnatal transfer of bone marrow and fetal liver progenitors rescues the defects in RPM and BMM in a Notch2-dependent manner, demonstrating the potential to restore defective tissue resident macrophage niches by Notch-competent progenitors. Thus, our study demonstrates that canonical Notch2 signalling is required for the development, maintenance, and function of iron-recycling macrophages.

## Abstract (web summary)

Red pulp and bone marrow macrophages play roles in iron homeostasis and erythropoiesis via molecular pathways not fully known. Here the authors show, via conditional deletion approaches in monocyte/macrophage progenitors in mice, that canonical Notch2 signalling is necessary for the development and function of iron-recycling macrophages.

## Introduction

Iron demands in mammals are largely supplied through the process of erythrophagocytosis and iron recycling from red blood cells (RBC), which is mainly carried out by iron-recycling macrophages. These are tissue-resident macrophage (TRM) populations comprising splenic red pulp (RP) macrophages (RPM), bone marrow (BM) macrophages (BMM) and liver Kupffer cells (KC)^1^.

The murine spleen (Spl) is organized in discrete microanatomical regions, each performing distinct functions^2^. The unique microanatomy of the Spl promotes the targeted elimination of red blood cells (RBC). This process takes place in the RP, where RPM phagocytose senescent, damaged, or infected erythrocytes, hemoglobin or heme complexes, and degrade them. The released iron is then either stored in the cell or shuffled back to the circulation, supplying most of the systemic iron necessary for erythropoiesis^3^. Recently, RPM has been implicated in host defense against pathogens such as *Streptococcus pneumoniae*, underscoring the significance of structure in relation to function^4,5^. The Spl also contains marginal zone (MZ) macrophages (MZM), marginal metallophilic macrophages (MMM) and white pulp (WP) macrophages (WPM)^6^. These macrophages are involved in clearance of blood-borne pathogens and control of adaptive immune responses^2^. Splenic CD169^+^ macrophages in the MZ transfer antigens to BATF3-dependent conventional dendritic cells (cDC) that promote the generation of antiviral effector CD8^+^ T cell responses^7^. Clodronate liposome depletion of MZM and MMM in mice causes enhanced spread of *Listeria monocytogenes* to peripheral organs, demonstrating a critical macrophage role in early infection control^8^. MZM depletion also leads to fatal autoimmunity by causing accumulation of exogenously administered apoptotic cells in the WP, further supporting the role of MZM in regulating immune activation and tolerance^9^, while WPM play a crucial role in clearance of apoptotic cells and maintain immune homeostasis within the germinal center^10^.

Like the Spl, the BM hosts distinct populations of macrophages^11^. BMM are specialized resident macrophages that function as iron-rich nurse cells for differentiating erythroblasts. They provide growth factors and iron critically needed for erythrocyte development and hemoglobin synthesis and facilitate erythropoiesis^3,12^. BMM exhibit high levels of proteins involved in iron homeostasis, sharing a similarity to the RPM machinery^13^, and selective deletion of BMM in conditions of erythropoietic stress leads to impaired erythropoietic recovery in mice^12,14,15^.

Previous studies have demonstrated that colony stimulating factor 1 (CSF-1), transcription factor Spi-C (encoded by *Spic*), peroxisome proliferator-activated receptor-γ (PPAR-γ, encoded by *Pparg*), Heme oxygenase 1 (HO-1, encoded by *Hmox1*), BACH1 and interleukin IL-33 are involved in development, maintenance and function of iron recycling RPM^16–22^. Mice lacking any of these factors exhibit reduced RPM numbers and present an iron overload phenotype in the Spl, emphasizing their essential role in the development and survival of RPM. However, despite recent progress in understanding ontogeny and heterogeneity of mononuclear phagocyte cells including TRM through advanced fate mapping studies, there remains a significant gap in our knowledge of their origin as well as in regulatory factors governing their development and the implications for disease contexts^11,23^.

Notch is a highly conserved signaling pathway that controls lineage commitment and cell development in tissues such as the mononuclear phagocyte system (MPS)^24,25^. For instance, Notch2, and its downstream regulator RBPJ (recombination signal binding protein for immunoglobulin κJ region) have been implicated in the development of specific cDC subsets in the Spl and intestine^26–30^. In the liver, sinusoidal endothelial cells seem to interact with recruited monocytes and drive delta-like 4 (DLL4)-Notch-RBPJ-mediated expression of *Nr1h3* and *Spic* genes with subsequent differentiation of monocytes into KC^31,32^. In the context of metabolic dysfunction-associated steatotic liver disease and/or steatohepatitis (MASLD/MASH), deletion of *Rbpj* alters the differentiation trajectory of Ly6C^hi^ monocytes from a pro-pathogenic macrophage phenotype toward a more protective Ly6C^lo^ patrolling monocyte, thereby mitigating some pathological features associated with MASLD/MASH^33^. RBPJ also regulates homeostasis and function of circulating Ly6C^lo^ monocytes by regulating CCR2 expression and controls development of CD16.2^+^ interstitial macrophages in the lung^34^.

Notch signaling interacts with CSF-1-signaling and controls macrophage maturation, promotes arteriogenesis and tissue repair during ischemia^35,36^. Previous work has shown that the DLL1-Notch2 signaling axis promotes the conversion of Ly6C^hi^ monocytes to Ly6C^lo^ patrolling monocytes, elucidating its role in the regulation of the MPS^25,37^.

Here, using *Cx3cr1*^*Cre*^-mediated targeting of components of the Notch signaling pathway and antibody-dependent blocking approaches, we show a previously unreported requirement for canonical Notch2 signaling in the development, maintenance and function of iron-recycling RPM and BMM, linking Notch2 activity to iron homeostasis and erythropoiesis.

## Results

### Canonical Notch2 signaling controls RPM and BMM development

To examine the role of Notch in the development of MPS, we generated mice with conditional deletion of *Notch2* (*N2*^*ΔCx3cr1*^) or *Rbpj* (*Rbpj*^*ΔCx3cr1*^) by crossing *Cx3cr1*^*Cre*^ mice^38^ with *Notch2*^*lox/lox*^^39^ or *Rbpj*^*lox/lox*^ animals^40^ and analyzed their offspring. This strategy targets embryonal and bone marrow progenitors of monocytes and macrophages, as well as mature phagocytes, and provides a practical approach to interrogate the role of *Notch2* within Cx3cr1^+^ lineages that potentially give rise to, and/or maintain iron-recycling macrophage populations. *Cre*-negative littermates served as controls (Ctrl) unless otherwise indicated.

Analysis of sorted Ctrl and *N2*^*ΔCx3cr1*^ RPM, BMM and Ly6C^hi^ populations revealed the presence of *Notch2* recombinant bands only in *N2*^*ΔCx3cr1*^ samples, confirming *Cre*-dependent specific recombination of the targeted locus (Supplementary Fig. 1a, b). Flow cytometry-based analysis (Supplementary Fig. 1c–h) revealed that within the splenic Ly6C^hi^ monocyte compartment, the frequency of Notch2^+^ cells was decreased in *N2*^*ΔCx3cr1*^ animals, and the deletion efficiency reached to ~21% and 51% in young and adult mice, respectively (Supplementary Fig. 1g, left panel), which was accompanied by decreased Notch2 MFI in monocytes (Supplementary Fig. 1g, right panel). Similarly, flow cytometry-based analysis showed effective deletion of Notch2 in BM Ly6C^hi^ and Ly6C^lo^ monocytes (deletion efficiency of ~34% and ~56%, respectively) as well as premonocytes and myeloid progenitors^41^. Although recombination was detectable in RPM and BMM populations (Supplementary Fig. 1a, b), evaluation of Notch2 surface expression in RPM and BMM was limited by their high autofluorescence (Supplementary Fig. 1e, f), and no differences were noted via flow cytometry (Supplementary Fig. 1h). QRT-PCR analysis further demonstrated the reduced *Notch2* expression at the mRNA level in the splenic monocyte populations, but not in RPM (Supplementary Fig. 1i). Collectively, our multi-dimensional analyses support *Cre*-dependent recombination of *Notch2*, concomitant with reduced Notch2 expression at the mRNA and surface protein expression levels in monocytes and myeloid progenitors, consistent with the reported activity of *Cx3cr1*^*Cre*^ in monocyte-macrophage lineages^38^ and confirm effective targeting in the monocyte compartment.

**Fig. 1:**
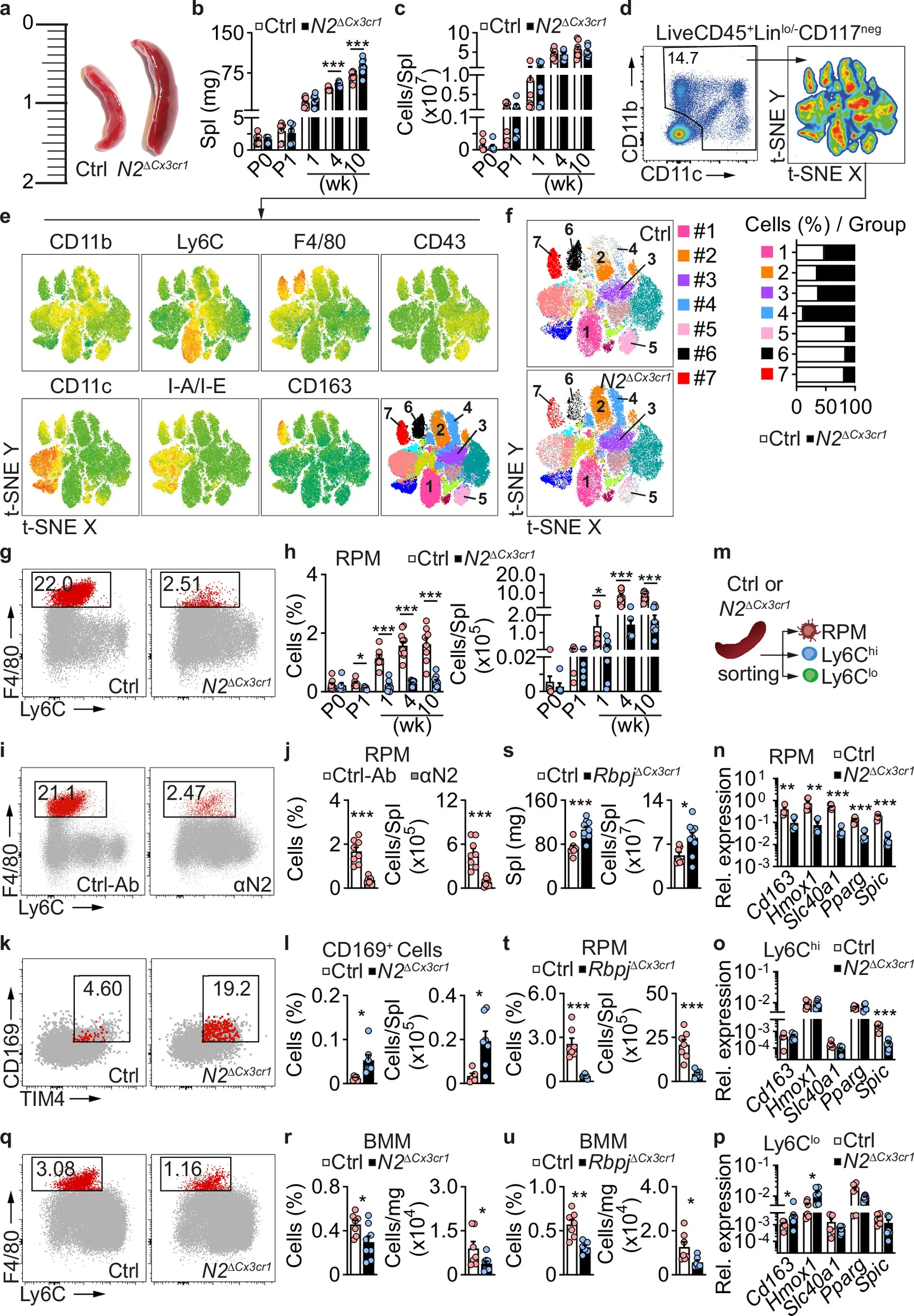
Canonical Notch2 signaling controls RPM and BMM development. **a**–**c** Splenomegaly in *N2*^*∆Cx3cr1*^ mice: (**a**) Spl size, (**b**) weight and (**c**) total cell numbers. **d**, **e** Gating strategy for t-SNE, PhenoGraph-based clustering and definition of splenic phagocytes (see also Supplementary Fig. 2i–m). **d** PhenoGraph-based clustering and mapping of phagocyte subsets on concatenated cells (**e**) using selected surface markers (*n* = 3/3). **f** t-SNE map of Ctrl or *N2*^*ΔCx3cr1*^ Spl (from Fig. 1e) showing altered composition of phagocytes clustered using PhenoGraph. **g** Representative flow cytometry plots showing Spl RPM in Ctrl and *N2*^*ΔCx3cr1*^ mice. Values are frequency of parent population (complete gating strategy in Supplementary Fig. 2n). **h** Relative frequency (defined from live cell gate) and absolute number of Spl RPM in Ctrl or *N2*^*ΔCx3cr1*^ mice. **i** Representative flow cytometry plots showing Spl RPM in wt mice treated with Ctrl-Ab or αN2-Ab. Values are frequency of parent population (complete gating strategy in Supplementary Fig. 2n). **j** Relative and absolute frequency of Spl RPM in Ctrl-Ab- or αN2-Ab-treated mice (*n* = 8/8). **k** Representative flow cytometry plot showing expansion of CD169^+^ cells in adult Spl. Values are frequency of parent population (see also Supplementary Fig. 3a, b). **l** Relative and absolute frequency of CD169^+^ cells in Spl (*n* = 5/7). **m** Experimental setup for sorting of RPM, Ly6C^hi^ and Ly6C^lo^ monocytes for gene expression analysis (gating in Supplementary Fig. 3e). **n**–**p** Expression of signature genes in RPM and splenic monocytes. **q** Representative flow cytometry plots showing BMM in Ctrl or *N2*^*ΔCx3cr1*^ mice. Values depict frequency of parent population (complete gating strategy as in Supplementary Fig. 2n). **r** Relative and absolute number of BMM normalized per mg BM in adult mice (*n *= 7/8). **s** Spl weight and total cell number in Ctrl or *Rbpj*^*ΔCx3cr1*^ mice (*n* = 7/9). **t**, **u** Relative and absolute frequency of RPM (**t**) and BMM (**u**) in Ctrl or *Rbpj*^*ΔCx3cr1*^ mice (*n* = 7/6). (**b, c**, **h**, **j**, **l**, **n**–**p**, **r**–**u**) Data are mean ± SEM pooled each from three experiments; exact n numbers (biological replicates) are provided in the Source Data. *P < 0.05; **P < 0.01; ***P < 0.001; (unpaired two-tailed Student’s *t* test). Red and blue data points indicate Ctrl and *N2*^*ΔCx3cr1 *^mice respectively. Source data are provided as a Source Data file.

*N2*^*ΔCx3cr1*^ mice exhibited splenomegaly starting with 4 weeks (wk) of age (Fig. 1a, b) but showed no changes in Spl total cell count and peripheral blood (PB) parameters (Fig. 1c, Supplementary Fig. 2a–h). We subjected Ctrl and *N2*^*ΔCx3cr1*^ Spl cells to flow cytometry and performed unsupervised t-distributed stochastic neighbor embedding (t-SNE) combined with PhenoGraph-based clustering analysis on concatenated CD45^+^Lin^lo/-^CD117^neg^ subsets after exclusion of CD11b/CD11c-double negative cells (Fig. 1d–f and Supplementary Fig. 2i). We identified 15 clusters with several subsets closely resembling Ly6C^hi^ (#1), CD43^+^Ly6C^lo^ monocytes (#5), CD11c^hi^I-A/I-E^+^ DCs (#8, #11) and F4/80^hi^ RPM (#6, #7) (Fig. 1e, f and Supplementary Fig. 2i, j). In *N2*^*∆Cx3cr1*^ mice, the frequency of F4/80^hi^ RPM (#6, #7), CD43^+^Ly6C^lo^ monocytes (#5) and CD11c^hi^CD11b^+^I-A/I-E^+^ DCs (#11) were strongly reduced (Fig. 1f and Supplementary Fig. 2k–m). At the same time, *N2*^*∆Cx3cr1*^ mice showed expansion of at least four subsets (#1, #2, #3, #4) of CD11b^+^CD11c^neg^ monocytes, which expressed altered levels of Ly6C, CD43 and CD163 (Fig. 1f and Supplementary Fig. 2j–m). Thus, *N2*^*ΔCx3cr1*^ mice show pronounced quantitative and qualitative changes in Spl MPS suggesting that *Notch2* regulates the development of RPM.

Next, we quantified RPM, BMM and Ly6C^hi^ monocytes in Spl and BM using a conventional flow cytometry gating strategy (Supplementary Fig. 2i, n, o and Supplementary Table 1), comparing Ctrl and *N2*^*ΔCx3cr1*^ mice from birth (P0, P1) to young (1 wk, 4 wk) and adulthood (10 wk) (Fig. 1g, h). In Ctrl mice, there was a rapid expansion of RPM from birth to around 1 wk of age, with numbers reaching a peak in 4 wk-old animals (Fig. 1g, h), similar to previous reports^21^. This rapid increase likely coincides with the expansion in blood volume and RBC mass during this period^42^. In contrast, RPM numbers in *N2*^*∆Cx3cr1*^ mice were reduced as early as postnatal day 1 (P1) and remained strongly suppressed into adulthood (10 wk) (Fig. 1g, h). Additional analysis of VCAM1 and MerTK expression confirmed the identity of RPM and BMM (Supplementary Fig. 2p–t). In addition, treatment of adult wild-type (wt) mice with anti-Notch2 (αN2) antibody, but not control antibody (Ctrl-Ab), was sufficient to phenocopy the loss of the RPM phenotype observed in *N2*^*∆Cx3cr1*^ animals (Fig. 1i, j).

At the same time, a CD169^+^ cell population expanded in *N2*^*∆Cx3cr1*^ mice (Fig. 1k, l and Supplementary Fig. 3a–c). The frequency of splenic Ly6C^hi^ monocytes did not differ between Ctrl and *N2*^*ΔCx3cr1*^ mice except at 4 wk of age, where there was a marked but transient increase in *N2*^*ΔCx3cr1*^ animals (Supplementary Fig. 3d).

To analyze the expression of RPM signature genes, we sorted RPM and monocytes from Ctrl and *N2*^*ΔCx3cr1*^ mice and performed quantitative real-time PCR (qRT-PCR) (Fig. 1m, and Supplementary Fig. 3e–h). The few remaining RPM in *N2*^*ΔCx3cr1*^ mice exhibited a reduced expression of *Cd163*, *Hmox1, Slc40a1*, *Pparg* and *Spic* (Fig. 1n). This coincided with downregulation of *Spic* in Ly6C^hi^ and upregulation of *Cd163* and *Hmox1* in Ly6C^lo^ monocyte subset from *N2*^*ΔCx3cr1*^ mice, suggesting a potential compensatory response to RPM loss (Fig. 1o, p). Similarly, the frequency of BMM was also reduced in *N2*^*ΔCx3cr1*^ mice, suggesting that *Notch2* plays a role in their development (Fig. 1q, r and Supplementary Fig. 3i).

To exclude a potential confounding effect of *Cx3cr1*^*Cre*^-mediated disruption of *Cx3cr1* function, we analyzed *Cx3cr1*^*gfp/+*^ (heterozygous) and *Cx3cr1*^*gfp/gfp*^ (homozygous) mice lacking one or both alleles of *Cx3cr1*^43,44^. Both strains displayed equal numbers of RPM (Supplementary Fig. 3j), suggesting that *Cx3cr1* loss-of-function does not account for RPM loss. Similarly, extended analysis of *N2*^*ΔCx3cr1*^ (*Cx3cr1*^*Cre/+*^ *Notch2*^*f/f*^) mice and three different respective controls namely, 1. Ctrl (*Cx3cr1*^*+/+*^ *Notch2*^*lox/lox*^), 2. *Cre-*negative *Notch2* non-floxed (*Cx3cr1*^*+/+*^ *Notch2*^*+/+*^), and 3. *Cre*-positive *Notch2* non-floxed (*Cx3cr1*^*Cre/+*^ *Notch2*^*+/+*^) animals excluded potential off-target effects of *Cre* activity (Supplementary Fig. 4a–e).

In *Rbpj*^*ΔCx3cr1*^ mice, conditional deletion of *Rbpj*, a key regulator of canonical Notch2 signaling^45^, recapitulated the phenotype of *N2*^*ΔCx3cr1*^ mice (Fig. 1s–u). In addition to splenomegaly and increased splenic cell numbers (Fig. 1s), *Rbpj*^*ΔCx3cr1*^ mice also exhibited substantial loss of RPM and BMM (Fig. 1t, u). Taken together, our data suggests that canonical Notch2 signaling controls RPM and BMM development in Spl and BM, respectively.

### CD163-expressing monocytes expand in *N2*^*ΔCx3cr1*^ mice

**Fig. 2:**
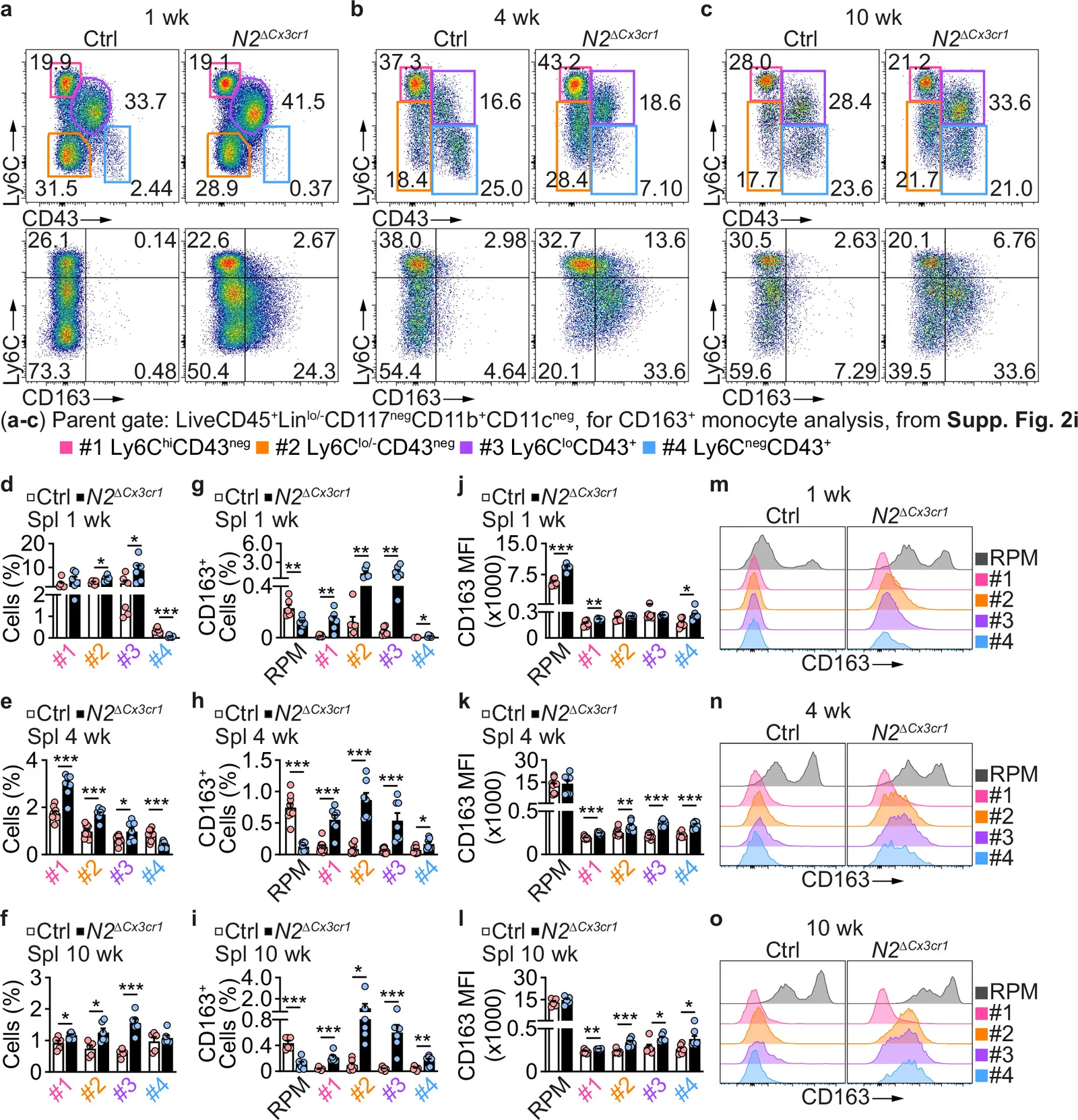
CD163-expressing monocytes expand in *N2*^*∆Cx3cr1*^ mice. **a**–**c** Representative flow cytometry plots showing Spl monocyte populations (#1-4, upper panel), and expression of CD163 in all monocytes (lower panel) from 1-, 4- and 10 wk-old mice. Values depict frequency of parent population (see also Supplementary Fig. 2i and Supplementary Fig. 5). **d**–**o** Analysis of monocyte subpopulations in 1-, 4- and 10 wk-old mice. **d**–**f** Relative frequency (from live cell gate) of monocyte populations. **g**–**i** Relative frequency (from live cell gate) of CD163^+^ cells within RPM and monocyte populations. **j**–**l** MFI of CD163 and (**m**–**o**) representative histograms showing CD163 expression. **d**–**l** Data are mean ± SEM. Each time point pooled from three experiments. *P < 0.05; **P < 0.01; ***P < 0.001; (unpaired two-tailed Student’s *t* test). 1 wk: *n* = 6/6; 4 wk: *n* = 10/7; 10 wk: *n* = 6/6. Red and blue data points indicate Ctrl and *N2*^*ΔCx3cr1*^mice respectively. Source data are provided as a Source Data file.

CD163 is a receptor involved in scavenging of haptoglobin-hemoglobin complexes^46^. Our gene expression analysis revealed that *N2*^*ΔCx3cr1*^ Ly6C^lo^ monocytes upregulate *Cd163* (Fig. 1p, Supplementary Fig. 4e), while PhenoGraph-based clustering showed an expansion of at least four monocyte subpopulations (#1-4) in *N2*^*ΔCx3cr1*^ mice (Fig. 1f). We hypothesize that RPM loss triggers the gradual expansion, from birth, of CD163^+^ monocytes as a compensatory mechanism to RPM loss in *N2*^*ΔCx3cr1*^ mice. Using a conventional gating strategy, we quantified splenic monocyte populations #1, #2, #3 and #4 and CD163 expression over time (Fig. 2a–c and Supplementary Fig. 5a–c). Compared to Ctrl, *N2*^*ΔCx3cr1*^ mice showed a gradual and sustained expansion of most monocyte subsets, starting with subset #2 in neonates (1 wk) but ultimately affecting subsets #1 and #3 from the age of 4 wk, while subset #4 was initially decreased but later recovered (Fig. 2d–f). Simultaneously, the frequency of CD163^+^ cells within monocytes (Fig. 2g–i), and CD163 expression levels (Fig. 2j–o), were strongly increased in all subsets in *N2*^*ΔCx3cr1*^ mice. At the same time, the frequency of CD163^+^ RPM was reduced early on and remained strongly suppressed, while CD163 expression in RPM was initially elevated but later returned to normal levels. In vitro phagocytosis assay using CFSE-labelled erythrocytes showed reduced phagocytic activity in remaining *N2*^*ΔCx3cr1*^ RPM, whereas Notch2-deficient monocytes displayed increased uptake compared to Ctrl counterparts (Supplementary Fig. 5d, e).

Interestingly, antibody-mediated blocking of Notch2 signaling in wt mice recapitulated the CD163 phenotype observed in *N2*^*ΔCx3cr1*^ mice. Upon Notch2 blockade, the monocyte subsets similarly expanded and CD163 expression increased (Supplementary Fig. 5f–j). Notably, residual RPM in αN2-treated wt mice displayed increased CD163 surface expression, as reflected by the increased CD163 MFI.

### Impaired canonical Notch2-signaling is associated with transcriptional changes across RPM and Ly6C^lo/int^ monocytes

**Fig. 3:**
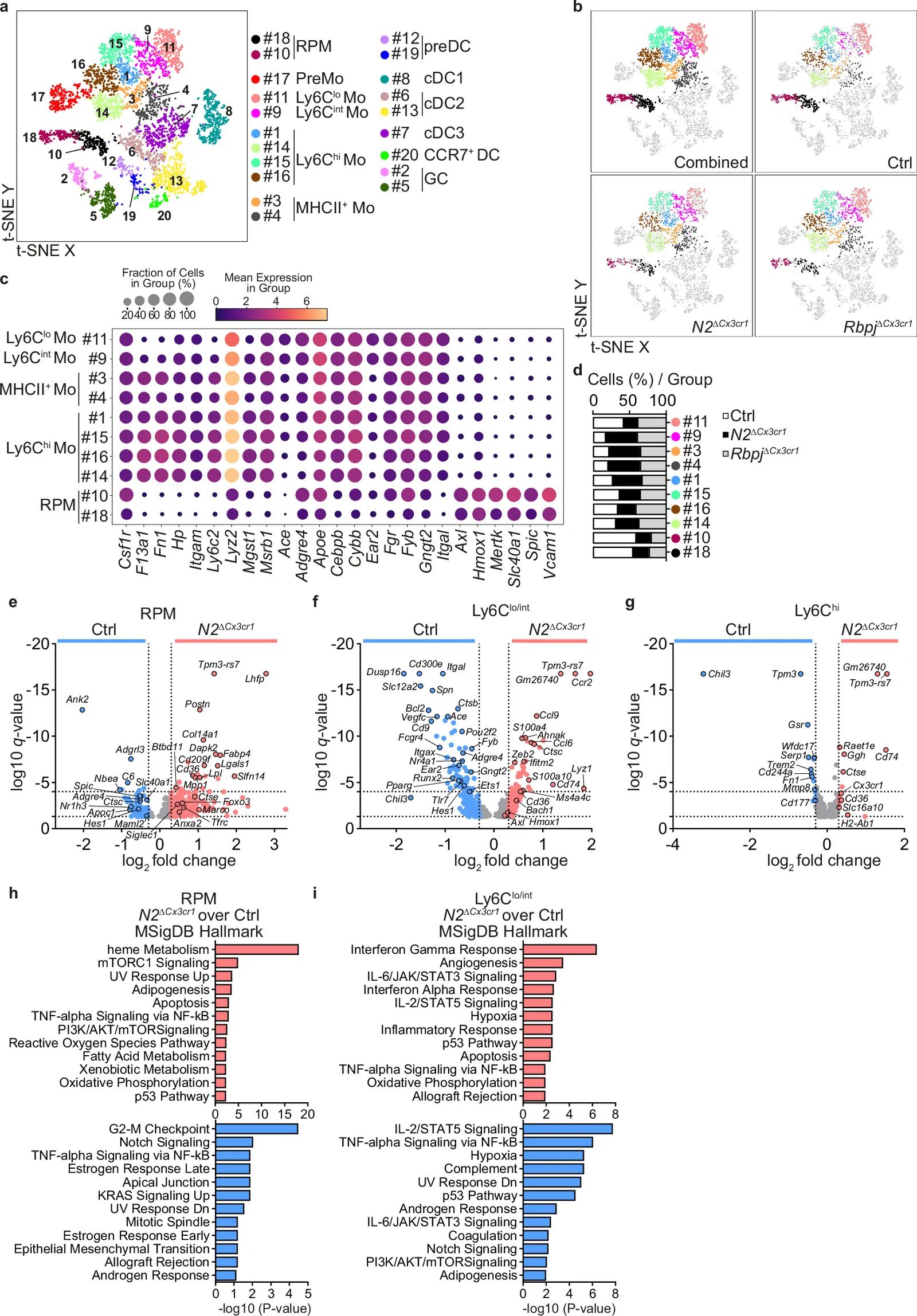
Impaired canonical Notch2-signaling is associated with transcriptional changes across RPM and Ly6C^lo/int^ monocytes. **a** Unsupervised t-SNE plot of scRNA-seq data of sorted live Lin^neg^ Spl myeloid cells. Populations were mapped using PhenoGraph-based clustering in concatenated cells from Ctrl, *N2*^*∆Cx3cr1*^ and *Rbpj*^*∆Cx3cr1*^ mice (*n* = 6633 cells). **b** t-SNE plots of monocyte and RPM subsets from concatenated data or individual genotypes after demultiplexing, corresponding to Ctrl, *N2*^*∆Cx3cr1*^, and *Rbpj*^*∆Cx3cr1*^. **c** Bubble heat map showing the expression of monocyte or RPM signature genes in the respective subsets. Color intensity shows the average expression of each gene. Circle sizes depict the percentage of cells expressing the gene. **d** Ratio of cells in monocytes and RPM clusters normalized to input cell numbers corresponding to Ctrl, *N2*^*∆Cx3cr1*^ and *Rbpj*^*∆Cx3cr1*^ mice. **e**–**g** Volcano plots showing differentially expressed genes (DEGs) in splenic (**e**) RPM, (**f**) Ly6C^lo/int^, and (**g**) Ly6C^hi^ monocytes between Ctrl and *N2*^*ΔCx3cr1*^ mice. Vertical dash lines indicate log_2_ fold change = 0.3 or −0.3. Horizontal lines indicate *q* value = 0.05 or 0.0001. **h**, **i** Bar chart of top 12 enriched Hallmark pathways from MSigDB for upregulated (red) or downregulated (blue) genes in *N2*^*∆Cx3cr1*^ (**h**) RPM or (**i**) Ly6C^lo/int^ monocytes compared to Ctrl. (*P* < 0.05). Plotted values represent *P* values computed via Enrichr (Fisher’s exact test) for the top 12 ranked terms; False Discovery Rates (FDR, *q* values) are provided in Supplementary Data 4, 5.

We labeled Ctrl, *N2*^*∆Cx3cr1*^ and *Rbpj*^*∆Cx3cr1*^ Spl cells with different hashtag-oligos (HTO)^47^, pooled them, sorted and subjected to exploratory single-cell RNA sequencing (scRNA-seq). We performed unsupervised t-SNE analysis followed by partitioning of scRNA-seq data into phenotypically distinct subpopulations using the PhenoGraph function from SeqGeq^48–50^. In the Spl, we identified 20 clusters and annotated them based on expression pattern of previously defined signature genes^17,51–54^ (Fig. 3a). Focusing on monocytes and RPM (Fig. 3b), gene expression analysis revealed prototypical gene expression patterns that correspond to cluster identity (Fig. 3c). Cluster #10 and #18 showed enriched expression of hallmark RPM genes (*Axl, Hmox1, Mertk, Slc40a1, Spic, Vcam1*) and both clusters were reduced in knockouts (Fig. 3d). Clusters of monocytes expressed hallmark genes of different monocyte subsets (Fig. 3c) and Ly6C^int^ monocytes (Cluster #9) were expanded as compared to Ctrl (Fig. 3d).

To gain exploratory insight into transcriptional programs associated with *Notch2* or *Rbpj* loss, we performed differential gene expression (DEG) analysis comparing *N2*^*∆Cx3cr1*^ *or Rbpj*^*∆Cx3cr1*^ RPM (#10, #18), Ly6C^lo/int^ (#9, #11) and Ly6C^hi^ (#1, #14, #15, #16) monocyte subsets to Ctrl counterparts (Supplementary Data 1–3).

*N2*^*∆Cx3cr1*^ RPM and Ly6C^lo/int^ monocyte subsets showed marked transcriptional changes, while changes in Ly6C^hi^ monocytes were minimal (Fig. 3e–g). The few remaining *N2*^*∆Cx3cr1*^ RPM were associated with increased expression of multiple genes, including *Mpp1*, *Tfrc*, *Foxo3*, *Lhfp, Postn, Fabp4, Lpl, Cd209f* and *Slfn14*, while *Spic*, *Slc40a1, Hes1*, *Nr1h3, Apoc1, Ctsc, Csf1r*, and *Maml2* were among the downregulated genes (Fig. 3e and Supplementary Data 1). Similarly, several patrolling-monocyte-associated genes (*Nr4a1, Fcgr4*, *Itgal*, *Itgax, Adgre4*, *Ace*, *Vegfc*, *Spn*, *Ear2* and *Gngt2*) appeared to be downregulated in Ly6C^lo/int^ monocyte in *N2*^*∆Cx3cr1*^ mice, while upregulation of *Ccr2*, *Ccl9*, *Ccl6, Cd74, Cd36, Nrp1*, *Zeb2, Ctsc, Ifitm2, S100a4, and S100a10* were observed together with modestly upregulated *Axl*, *Bach1*, and *Hmox1*–genes typical for RPM (Fig. 3f and Supplementary Data 2).

To identify potential candidate pathways associated with these transcriptional changes, exploratory enrichment analysis using Enrichr^55–57^ with Molecular signatures database (MSigDB) Hallmark gene sets^58^ was performed. This suggested that *N2*^*∆Cx3cr1*^ RPM were enriched for upregulated genes associated with heme metabolism, alongside metabolic- and lipid-associated processes such as mTORC1 Signaling, oxidative phosphorylation, as well as fatty acid metabolism and adipogenesis (Fig. 3h and Supplementary Data 4). Additional exploratory analysis with Gene Ontology (GO) Biological Process gene sets^59,60^ suggested enrichment in GO terms associated with aerobic electron transport chain, mitochondrial electron transport, and cellular respiration (Supplementary Fig. 6a and Supplementary Data 4). Downregulated genes in *N2*^*∆Cx3cr1*^ RPM were associated with cell cycle and signaling pathways, including Notch while GO terms related to calcium homeostasis, as well as lipid metabolism (Regulation of Fatty Acid Biosynthetic Process) and Negative Regulation of Erythrocyte Differentiation were enriched (Fig. 3h and Supplementary Fig. 6a).

Upregulated genes in *N2*^*∆Cx3cr1*^ Ly6C^lo/int^ monocytes appeared to be enriched for inflammatory pathways, including interferon gamma response and IL-6/JAK/STAT3 signaling (Fig. 3i and Supplementary Data 5), and were also associated with enrichment of response to cytokine and phagocytosis, and positive regulation of reactive oxygen species metabolic processes by GO (Supplementary Fig. 6b and Supplementary Data 5). Downregulated genes in this subset appeared to be enriched for IL-2/STAT5 signaling and TNF-alpha Signaling via NF-κB, while GO analysis also suggested enrichment of terms related to positive regulation of intracellular signal transduction and regulation of inflammatory response (Fig. 3i and Supplementary Fig. 6b).

Similar to the *N2*^*∆Cx3cr1*^, DEG analysis of *Rbpj*^*∆Cx3cr1*^ RPM and monocyte subsets revealed most transcriptional changes in the RPM and Ly6C^lo/int^ monocytes but not in Ly6C^hi^ monocytes (Supplementary Fig. 7a–c and Supplementary Data 1–3). *Rbpj*^*∆Cx3cr1*^ RPM shared similarly upregulated genes with *N2*^*∆Cx3cr1*^ RPM, including *Mpp1*, *Tfrc, Fabp4, Lpl, Cd209f, and Slfn14* while *Cd163*, *Pparg*, *Ctsc*, *Apoc1, Apoe*, and *Hif1a* were downregulated (Supplementary Fig. 7a and Supplementary Data 1).

The Ly6C^lo/int^ subset in *Rbpj*^*∆Cx3cr1*^ mice similarly exhibited substantial transcriptional changes compared to Ctrl, with upregulation of *Ccr2, Ccl6, Ccl9, Cd36, Cd74, Slamf9, Zeb2, S100a4, S100a10, Ifitm2, and Ctsc* as well as *Hmox1* and *Bach1*, and downregulation of *Nr4a1, Ace, Vegfc*, *Spn*, *Itgal, Itgax, Adgre4*, and *Hes1* (Supplementary Fig. 7b and Supplementary Data 2).

Heme metabolism was also the top-enriched pathway in *Rbpj*^*∆Cx3cr1*^ RPM (Supplementary Fig. 7d and Supplementary Data 6). *Rbpj*^*∆Cx3cr1*^ RPM upregulated genes were likewise associated with positive regulation of protein localization to nucleus and aerobic electron transport chain, while downregulated genes were enriched for interferon gamma response and type II interferon-mediated signaling pathway by GO (Supplementary Fig. 7d, e).

Enrichment analysis of upregulated genes in *Rbpj*^*∆Cx3cr1*^ Ly6C^lo/int^ monocytes identified terms associated with metabolic and stress-related pathways, such as mTORC1 signaling and hypoxia, while downregulated genes were enriched for complement and TNF-alpha signaling via NF-κB (Supplementary Fig. 7f and Supplementary Data 7). Upregulated genes were enriched for TOR signaling and mitochondrial transport. Downregulated genes were associated with intracellular signaling cassette as well as negative regulation of cell cycle (Supplementary Fig. 7g).

Comparing DEGs between *N2*^*∆Cx3cr1*^ and *Rbpj*^*∆Cx3cr1*^ revealed that the majority of shared transcriptional changes were in RPM and Ly6C^lo/int^ subsets with substantial overlap in both upregulated and downregulated genes (Supplementary Fig. 7h, i). In contrast, Ly6C^hi^ monocytes were associated with markedly fewer overlapping genes, which may suggest more limited transcriptional changes in *N2*^*∆Cx3cr1*^ and *Rbpj*^*∆Cx3cr1*^ mice in this subset (Supplementary Fig. 7j).

Together, these findings support the observed phenotypes and suggest that conditional targeting of *Notch2* and its nuclear mediator *Rbpj* is associated with broad alterations in transcriptional programs across the remaining few RPM and in expanded Ly6C^lo/int^ monocytes.

### Impaired splenic microarchitecture and iron homeostasis in canonical *Notch2* signaling-deficient mice

**Fig. 4:**
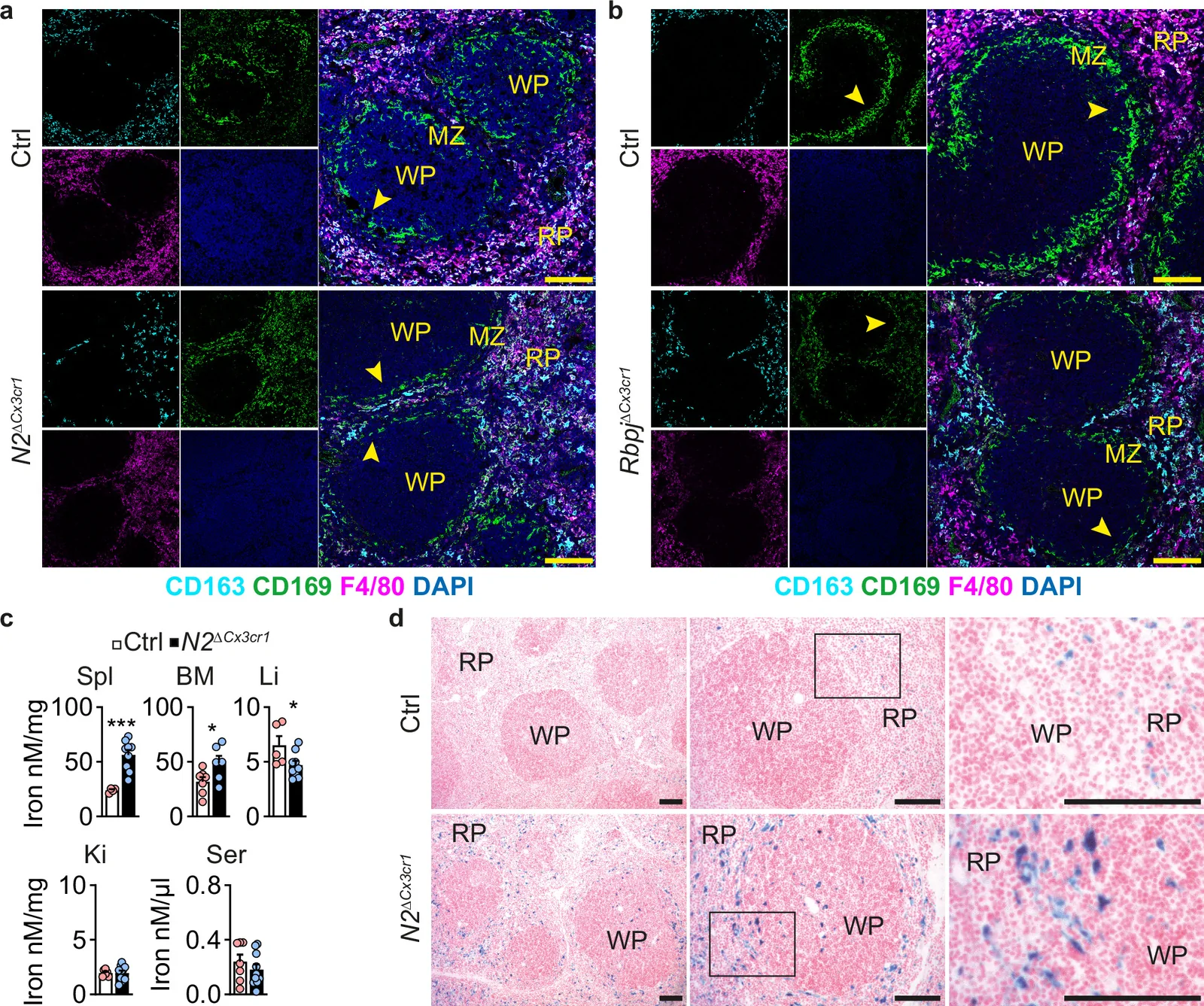
Impaired splenic microarchitecture and iron homeostasis in canonical *Notch2* signaling-deficient mice. **a**, **b** CLSM images of Spl from 10 wk old (**a**) Ctrl or *N2*^*ΔCx3cr1*^- and (**b**) Ctrl or *Rbpj*^*ΔCx3cr1*^ mice. Arrows indicate CD169^+^ MZ ring corresponding to MMM. Note the ring disruption in *N2*^*ΔCx3cr1*^ mice (see also Supplementary Fig. 8 and Supplementary Video 1, 2). **c** Iron quantification in Spl, BM, liver (Li), kidney (Ki) and serum (Ser) of mice. Data are mean ± SEM. Spl: *n* = 4/9; BM: *n* = 7/6; Li: *n* = 5/9; Ki: *n* = 6/10; Ser: *n* = 7/10. Pooled from three independent experiments. *P < 0.05; **P < 0.01; ***P < 0.001; (unpaired two-tailed Student’s *t*-test). Red and blue data points indicate Ctrl and *N2*^*ΔCx3cr1*^mice respectively. Source data are provided as a Source Data file. **d** Perl’s Prussian blue staining showing iron deposits in 10 wk old Spl of Ctrl and *N2*^*ΔCx3cr1*^ mice. **a**, **b**, **d** Representative of three independent experiments. Scale bar 100 µm. See also Supplementary Fig. 9.

We next investigated the splenic microarchitecture and iron metabolism. Confocal laser scanning microscopy (CLSM) revealed a normal splenic architecture in Ctrl mice, as previously described^2^. Specifically, there was strong, homogenous F4/80 and CD163 co-staining in the RP, which was separated from the WP by a defined CD169^+^ MZ ring structure consisting of CD169^+^ MMM (Fig. 4a, b). In contrast, in *N2*^*ΔCx3cr1*^ mice, F4/80 staining was reduced and the MZ ring structure was disrupted, with a shift of the CD169 signal to the RP colocalizing with CD163 (Fig. 4a and Supplementary Fig. 8a, b). A similar phenomenon was observed in *Rbpj*^*ΔCx3cr1*^ mice, confirming that canonical Notch2-signaling is responsible for this phenotype (Fig. 4b and Supplementary Fig. 8c). A 3D reconstruction of the Spl showed the stark differences between genotypes: F4/80^+^ RPM predominantly populated the RP in Ctrl, resulting in a dense RP appearance while *N2*^*ΔCx3cr1*^ mice showed a sparse RP with scattered CD169 positivity (Supplementary Video 1, 2). No changes were observed in CD68, which stains RPM and WPM, as well as MARCO and Tim4, which stain subsets of MZM and MMM (Supplementary Fig. 8b–e). Similarly, F4/80, CD169 and Tim4 staining, as well as splenic microarchitecture remained unchanged in *Cx3cr1*^*gfp/+*^ and *Cx3cr1*^*gfp/gfp*^ mice (Supplementary Fig. 8f, g), confirming data obtained by flow cytometry (Supplementary Fig. 3j). Collectively, these data show that the deletion of *Notch2* or *Rbpj* significantly alters the splenic microarchitecture due to changes in RPM and CD169^+^ MMM in the splenic niche.

Next, we tested whether changes in the RPM pool result in impaired iron recycling in *N2*^*∆Cx3cr1*^ mice. Despite normal PB parameters (Supplementary Fig. 2a–h), there was iron accumulation in Spl and BM, but not in kidney or serum, and a reduction of iron content in the liver in *N2*^*∆Cx3cr1*^ mice (Fig. 4c and Supplementary Fig. 9). Perl’s Prussian Blue staining confirmed visible and focal iron deposition in the RP of *N2*^*∆Cx3cr1*^ Spl sections (Fig. 4d). Consistent with flow cytometry findings (Supplementary Fig. 4), the iron overload in the Spl and BM was specific to *N2*^*∆Cx3cr1*^ mice and was not recapitulated in *Cre*-positive controls, arguing against *Cre* off-target activity as the nonspecific driver of this phenotype (Supplementary Fig. 10a–c).

### Deletion of *Notch2* does not affect RPM proliferation or cell death

To investigate whether proliferation differences could account for the early onset impairment in RPM development, we performed Ki-67 staining. In situ analysis by CLSM showed prominent Ki-67 staining in the RP of both Ctrl and *N2*^*ΔCx3cr1*^ mice at the age of 2 wk, which was absent in 10 wk-old mice, indicating transient proliferation preceding full maturation (Supplementary Fig. 11a, b). The majority of these proliferating cells were located in the RP with a few Ki-67^+^ cells in the WP (Supplementary Fig. 11a). We found bright F4/80^+^ cells that partially colocalized with Ki-67 in the RP, and with concentric CD169^+^ MMM rings in young Ctrl mice, suggestive of the proliferative capacity of RPM during the onset of normal splenic architecture formation (Supplementary Fig. 11a, b). In contrast, young *N2*^*ΔCx3cr1*^ Spl already showed the evident loss of RPM and aberrant CD169 staining in the RP and although Ki-67 was present, these cells were not positive for F4/80 (Supplementary Fig. 11a, b). BrdU incorporation in RPM was similar in both strains (Supplementary Fig. 11c), suggesting that deletion of *Notch2* does not affect proliferation capacity of RPM. We also analyzed cell death by Annexin V (AnnV) and propidium iodide (PI) staining of Spl cells. There was no significant difference in the number of apoptotic or necrotic cells within the RPM population from Ctrl and *N2*^*∆Cx3cr1*^ mice (Supplementary Fig. 11d). Thus, deletion of *Notch2* affects neither death nor proliferation ability of RPM.

### Reconstitution of the splenic niche with BM monocyte-derived RPM

**Fig. 5:**
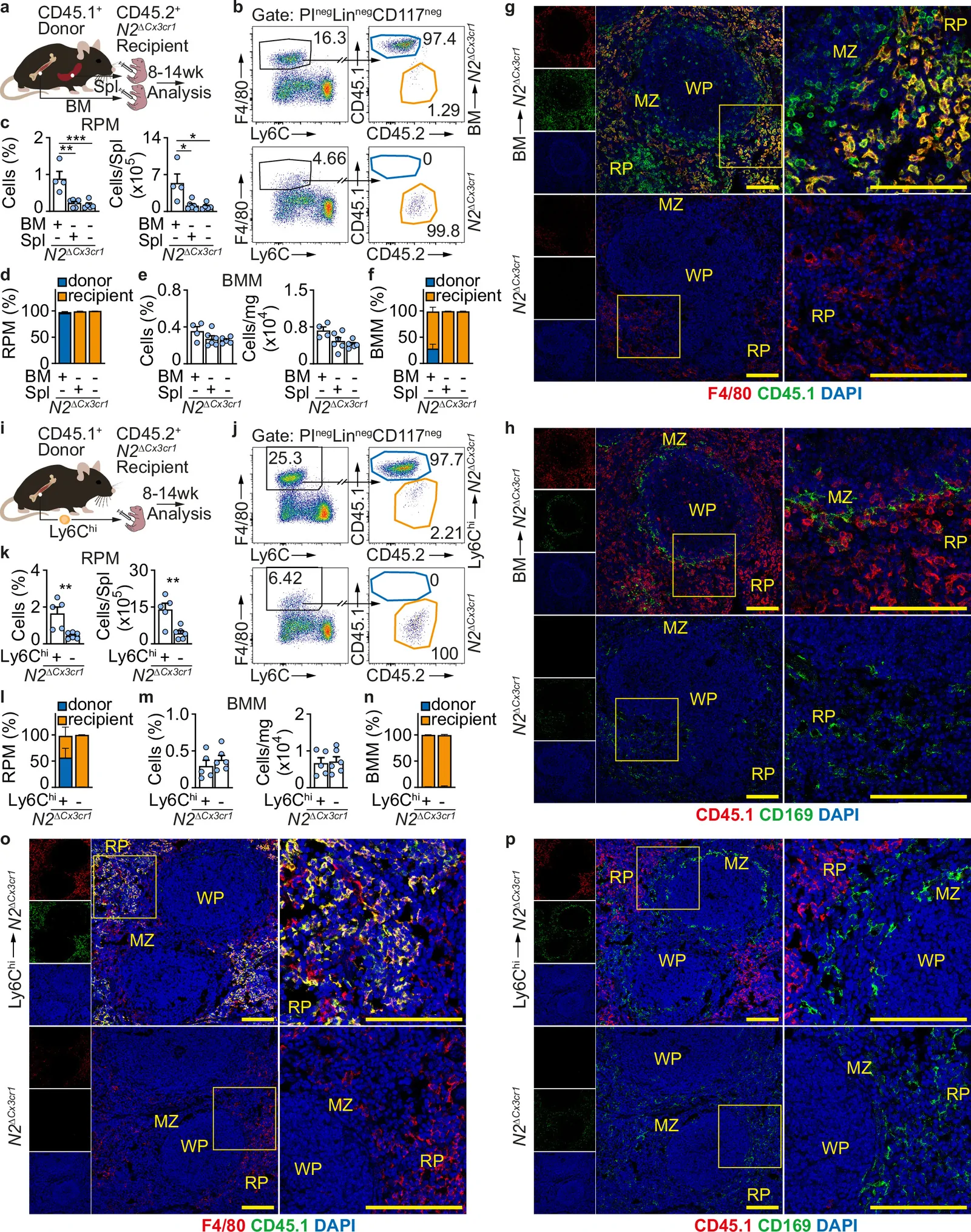
Reconstitution of the splenic niche with BM monocyte-derived RPM. **a** Experimental scheme of reconstitution studies. **b** Representative flow cytometry plots showing donor-derived RPM in *N2*^*ΔCx3cr1*^ recipient Spl. Values indicate frequency of parent population (see also Supplementary Fig. 12a). **c** Relative (from live cell gate) and absolute frequency of RPM in recipient mice. **d** Frequency of donor- and recipient-derived RPM in whole RPM pool. **e** Relative and absolute frequency of BMM calculated per mg BM in recipient mice. **f** Frequency of donor- and recipient-derived BMM in whole BMM pool of recipients. **g**, **h** CLSM images of Spl from BM-reconstituted-, or non-reconstituted *N2*^*ΔCx3cr1*^ mice. Images are from the same sample but shown separately for clarity. **i** Experimental scheme of reconstitution studies using BM Ly6C^hi^ monocytes. **j** Representative flow cytometry plots showing donor-derived RPM after reconstitution. **k** Relative and absolute frequency of RPM in recipient mice. **l** Frequency of donor- and recipient-derived RPM in whole RPM pool. **m** Relative and absolute frequency of BMM in recipient mice. **n** Frequency of donor- and recipient-derived BMM in whole BMM pool. **o**, **p** Spl CLSM images from recipient *N2*^*ΔCx3cr1*^ mice reconstituted or not with BM Ly6C^hi^ monocytes. Images are from the same sample but shown separately for clarity. (**c**–**f**, **k**–**n**) Mean ± SEM pooled from (**c**–**f**) two (*n* = 4/6/5) or (**k**–**n**) three (*n* = 5/6) experiments are shown. (**c**, **e**) *P < 0.05; **P < 0.01; ***P < 0.001 (One-way ANOVA with Bonferroni’s multiple comparison test). **k**, **m** *P < 0.05; **P < 0.01; ***P < 0.001; (unpaired two-tailed Student’s *t* test). **g**, **h**, **o**, **p** Representative of two (**g**, **h**) or three (**o**, **p**) experiments. Scale bar 100 µm. See also Supplementary Fig. 12. Blue data points indicate *N2*^*ΔCx3cr1*^ recipient mice. Source data are provided as a Source Data file.

To test whether Notch2 controls cell differentiation or cell commitment to an RPM fate, we performed several adoptive transfer studies. First, we transferred CD45.1^+^ whole BM or whole Spl cells into CD45.2^+^ *N2*^*∆Cx3cr1*^ neonates and analyzed the CD45.1^+^ donor population 8–14 weeks later (Fig. 5a and Supplementary Fig. 12a). BM cells gave rise to RPM which reconstituted the defective niche and rescued the *N2*^*∆Cx3cr1*^ phenotype, while donor Spl cells failed to do so (Fig. 5b–d and Supplementary Fig. 12b). Neither BM nor Spl cell transfer rescued BMM (Fig. 5e, f) despite the presence of a distinct donor-derived population (~30%) in recipients of BM (Fig. 5f), nor did it change the total number of BM cells in recipients (Supplementary Fig. 12c).

CLSM revealed a strong F4/80 signal in the RP and defined CD169^+^ MMM ring in the Spl of BM-reconstituted mice (Fig. 5g, h, Supplementary Fig. 12d), phenocopying the normal splenic architecture seen in Ctrl (Fig. 4a, b). The F4/80^+^ RPM costained with CD45.1, confirming their donor origin. This was not the case for CD169, which did not colocalize with CD45.1 (Fig. 5h). In line with these data, we saw little or no accumulation of iron in BM-reconstituted *N2*^*∆Cx3cr1*^ Spl, supporting a phenotype rescue (Supplementary Fig. 12e, f).

We next hypothesized that BM Ly6C^hi^ monocytes are capable of differentiating into RPM in the defective splenic niche. To test this, we transferred sorted CD45.1^+^ BM Ly6C^hi^ monocytes into CD45.2^+^ *N2*^*∆Cx3cr1*^ newborns and analyzed their fate after 8–14 weeks (Fig. 5i and Supplementary Fig. 12a). BM Ly6C^hi^ monocytes reconstituted the defective niche and rescued RPM numbers compared to non-reconstituted *N2*^*∆Cx3cr1*^ mice (Fig. 5j–l). As with whole BM reconstitution, RPM were mostly (~56%) donor-derived (Fig. 5l), while the frequency of Spl CD45^+^ cells remained unchanged (Supplementary Fig. 12g). Transfer of Ly6C^hi^ monocytes influenced neither BMM nor total BM cells (Fig. 5m, Supplementary Fig. 12h) and the BMM pool present in reconstituted *N2*^*∆Cx3cr1*^ mice was of recipient origin (Fig. 5n). Again, characteristic F4/80^+^ RPM in the RP and a defined CD169^+^ MMM ring were present in recipients of CD45.1^+^ Ly6C^hi^ monocytes but not in *N2*^*∆Cx3cr1*^ animals without reconstitution (Fig. 5o, p and Supplementary Fig. 12i). F4/80 colocalized with CD45.1, revealing the donor origin of RPM (Fig. 5o), but the CD169^+^ MMM ring structure were mainly CD45.1 negative (Fig. 5p). Despite successful repopulation with BM Ly6C^hi^ monocytes, the Spl of reconstituted *N2*^*∆Cx3cr1*^ mice showed iron deposits in the RP (Supplementary Fig. 12j, k). Thus, BM Ly6C^hi^ monocytes do not fully functionally rescue the iron accumulation phenotype seen in the *N2*^*∆Cx3cr1*^ mice.

### Development of RPM and BMM requires intrinsic Notch2 signaling

To directly address the requirement of intrinsic *Notch2* for RPM and BMM development, we generated *N2*^*∆Cx3cr1*^ *mG* (KO *mG*) and Ctrl *mG* strains by crossing *ROSA26-mT/mG* mice^61^ with *Cx3cr1*^*Cre*^ *Notch2*^*lox/lox*^ *(N2*^*ΔCx3cr1*^) or *Cx3cr1*^*Cre*^ *Notch2*^*+/+*^ (Ctrl) animals, respectively. This targeting strategy leads to the simultaneous deletion of *Notch2* and expression of *mG* (membrane-targeted GFP) in *Cx3cr1*-expressing cells and their progeny, which can be used for repopulation and subsequent cell fate studies specifically addressing the role of Notch2.

**Fig. 6:**
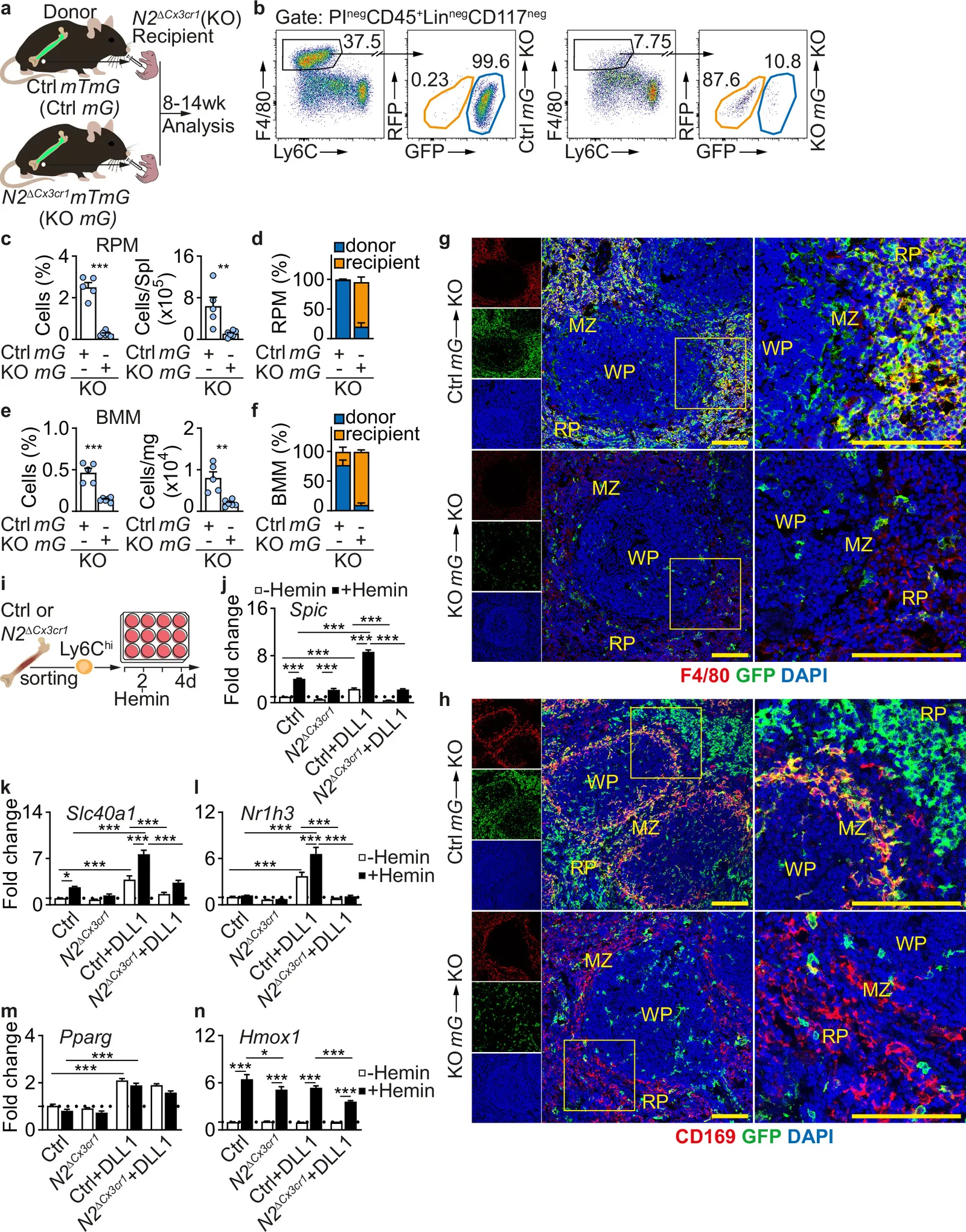
Development of RPM and BMM requires intrinsic Notch2 signaling. **a** Experimental scheme of reconstitution study. **b** Representative flow cytometry plots showing donor-derived RPM in *N2*^*ΔCx3cr1*^ recipients. Values indicate frequency of parent population (see also Supplementary Fig. 13a). **c** Relative (from live cell gate) and absolute frequency of RPM in recipient mice. **d** Frequency of donor- and recipient-derived RPM in whole RPM pool. **e** Relative and absolute frequency of BMM calculated per mg BM in recipient mice. **f** Frequency of donor- and recipient-derived BMM in whole BMM pool. **g, h** CLSM images of Spl from Ctrl *mG* or KO *mG* BM-reconstituted *N2*^*ΔCx3cr1*^ mice. Representative of three experiments. Scale bar 100 µm. **c**–**f** Data are mean ± SEM and pooled from three experiments (*n* = 5/6). (**c, e**) *P < 0.05; **P < 0.01; ***P < 0.001; (unpaired two-tailed Student’s *t* test). Blue data points indica*t*e *N2*^*ΔCx3cr1*^ recipient mice. See also Supplementary Fig. 13. **i** Experimental scheme: Sorted BM Ly6C^hi^ monocytes cultured in vitro with Ctrl or DLL1 ligands, CSF-1 and Hemin and differentiated into RPM-like macrophages. **j**–**n** Expression of RPM signature genes in RPM-like macrophages generated from Ctrl or *N2*^*ΔCx3cr1*^ Ly6C^hi^ monocytes in vitro (*n* = 8/8). Data are mean ± SEM from three experiments. *P < 0.05; **P < 0.01; ***P < 0.001; Two-way ANOVA with Bonferroni’s multiple comparison test. Source data are provided as a Source Data file.

We transferred BM cells from Ctrl *mG* or KO *mG* mice into newborn *N2*^*∆Cx3cr1*^ animals and analyzed the recipients 8-14 wk later (Fig. 6a, b and Supplementary Fig. 13a). Ctrl *mG* BM, but not KO *mG* BM restored RPM, which were donor-derived (Fig. 6b–d) and prevented splenomegaly without altering total splenic CD45^+^ cells in recipients (Supplementary Fig. 13b). Furthermore, the transfer of Ctrl *mG* BM cells in *N2*^*∆Cx3cr1*^ mice also rescued BMM, which were mostly (~80%) of donor origin but the total number of BM cells remained unchanged in recipient mice (Fig. 6e, f, and Supplementary Fig. 13c).

By CLSM, mice reconstituted with Ctrl *mG* BM showed F4/80^+^ RPM in the RP and CD169^+^ MMM rings that stained positive for GFP, revealing donor origin (Fig. 6g, h). In contrast, KO *mG* BM-reconstituted mice contained few F4/80^+^ cells and lacked the defined CD169^+^ MMM rings (Fig. 6g, h). Furthermore, Ctrl *mG* but not KO *mG* BM-reconstituted animals exhibited lower iron levels and only minimal iron deposits in the Spl, again suggesting a rescued phenotype (Supplementary Fig. 13d, e).

Fetal liver (FL) cells can colonize empty niches and develop into functional tissue resident macrophages including RPM^21,62^. In this context, we transferred E14.5 Ctrl *mG*, and KO *mG* FL cells into *N2*^*∆Cx3cr1*^ neonates and analyzed RPM development in recipients at 8-14 wk (Supplementary Fig. 14a, b). Ctrl *mG* FL cells restored RPM and BMM pools with cells of donor origin (Supplementary Fig. 14b–f) and rescued the splenomegaly phenotype (Supplementary Fig. 14g, h), while the few remaining RPM and BMM in KO *mG* FL-reconstituted mice were recipient-derived cells (Supplementary Fig. 14d, f). In situ analysis revealed that Ctrl *mG* FL-reconstitution, but not KO *mG* FL-reconstitution, restored RPM and CD169^+^ MMM subsets and splenic microarchitecture in recipients (Supplementary Fig. 14i, j), similar to Ctrl *mG* BM-reconstitution (Fig. 6g, h). As expected, Ctrl *mG* FL-reconstituted Spl showed reduced iron deposits in contrast to KO *mG* FL-reconstituted mice, which had abundant diffuse iron inserts in the RP (Supplementary Fig. 14k, l).

When pulsed at P5 / P7, at the time point when our repopulation studies were performed, inducible fate mapping models, *Ms4a3*^*CreERT2*^*Rosa*^*LSL-tdT*^ (*Ms4a3*^*itdT*^*)* and *Cx3cr1*^*CreERT2*^*Rosa*^*LSL-YFP*^ (*Cx3cr1*^*iYFP*^*)*, label few RPM and BMM ( ~ 1–5%) in adult mice, suggesting that under steady state conditions, RPM and BMM pools might not be fully developed at the time of pulsing or are derived from progenitors that do not express *Ms4a3* or *Cx3cr1* at these early postnatal stages, with substantial contribution occurring at earlier or later time points (Supplementary Fig. 14m). At the same time, constitutive *Ms4a3*^*Cre*^*Rosa*^*LSL-tdT*^ (*Ms4a3*^*tdT*^) targets ~10% of RPM while Ctrl *mG* mice show 100% targeting, suggesting minimal contribution of GMP (granulocyte-monocyte progenitor) lineage to adult RPM or a high turnover rate of monocyte-derived subsets within the RPM pool (Supplementary Fig. 14n).

In the BM, constitutive *Ms4a3*^*tdT*^ targeted ~50% of BMM while Ctrl *mG* mice similarly showed 100% targeting (Supplementary Fig. 14n). This difference likely reflects distinct kinetics of Spl and BM macrophage niche formation, as was suggested by Chen et al.^42^, with the BM niche forming later than the Spl, and in parallel with the development of GMP. Nevertheless, independent of origin, Notch2 is indispensable for the development of both RPM and BMM. Thus, Notch2 is unequivocally required for the development of RPM and BMM which can arise from BM or FL progenitors.

### DLL1 - Notch2 regulates heme-induced RPM signature genes in vitro

Heme functions as a physiological cue to promote monocyte differentiation into iron-recycling macrophages^17^. To test whether Notch2 regulates RPM signature genes in response to heme in a defined in vitro system, we cultured Ctrl or *N2*^*ΔCx3cr1*^ BM Ly6C^hi^ monocytes on immobilized recombinant Notch ligand DLL1 in the presence of CSF-1 and hemin and analyzed the resultant macrophages for the expression of signature RPM genes (*Spic*, *Slc40a1*, *Nr1h3*, *Pparg*, *Hmox1*) (Fig. 6i).

Hemin and DLL1 upregulated *Spic and Slc40a1* (encoding ferroportin) individually and synergistically, which was mediated by *Notch2*, while *Nr1h3* (encoding LXR-alpha) was strictly dependent on DLL1-Notch2 (Fig. 6j–l). *Pparg* was expressed in cells cultured on DLL1, but was not dependent on *Notch2* or hemin (Fig. 6m). *Hmox1*, in contrast, was strictly hemin-dependent and only partially regulated by Notch2 (Fig. 6n). These data indicate that DLL1-Notch2 signaling in conjunction with hemin promotes expression of *Spic, Slc40a1* and *Nr1h3*, and is required for RPM development.

### Enhanced extramedullary erythropoiesis in *N2*^*∆Cx3cr1*^ mice

**Fig. 7:**
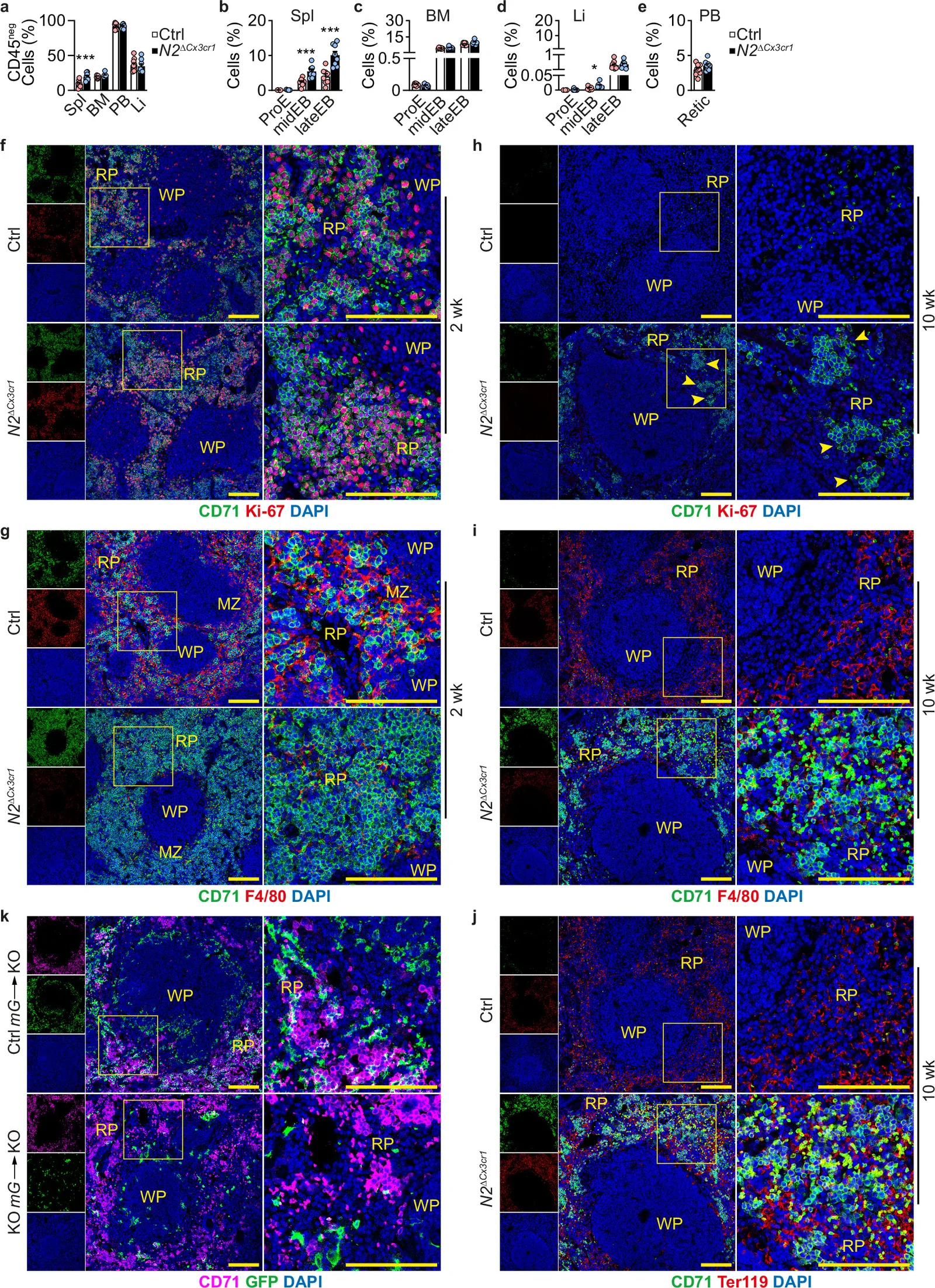
Enhanced extramedullary erythropoiesis in *N2*^*∆Cx3cr1*^ mice. **a**–**e** Relative frequency of (**a**) CD45^neg^ cells, **b**–**d** erythroid precursors in the Spl, BM, liver (Li) and (**e**) reticulocytes in PB. Data are mean ± SEM pooled from three experiments; exact *n* numbers are provided in the Source Data. *P < 0.05; **P < 0.01; ***P < 0.001; (unpaired two-tailed Student’s *t*-test). Red and blue data points indicate Ctrl and *N2*^*ΔCx3cr1*^mice respectively. Source data are provided as a Source Data file. See also Supplementary Fig. 15a, b and Supplementary Table 1. **f**–**j** Spl CLSM images from 2 wk (**f**, **g**), or 10 wk (**h**–**j**) old Ctrl or *N2*^*ΔCx3cr1*^ mice. **h** Arrowheads depict erythropoietic clusters. **f**, **g** Images are from the same sample but shown separately for clarity. **i**, **j** Images are from the same sample but shown separately for clarity. See also Supplementary Fig. 16c. **k** CLSM images of *N2*^*ΔCx3cr1*^ Spl reconstituted with Ctrl *mG* or KO *mG* BM cells. **f**–**k** Images are representative of three experiments. Scale bar 100 µm.

During erythropoietic stress, erythropoiesis shifts to extramedullary sites such as the Spl, thus expanding production of reticulocytes and mature erythrocytes^63^. Flow cytometry analysis revealed that *N2*^*∆Cx3cr1*^ mice exhibit an expansion of CD45^neg^ erythroid precursors, midEB and lateEB^64,65^ specifically in the Spl and minor changes in liver (Fig. 7a–e and Supplementary Fig. 15a, b). An alternative staining to identify reticulocytes and erythroid precursors using the nucleic acid stain thiazole orange (TO) (Supplementary Fig. 15c, d) yielded similar results consistent with those obtained without TO (Supplementary Fig. 15e–i, and Fig. 7a–e).

Antibody-mediated blocking of Notch2 signaling in wt mice recapitulated the observed expansion of CD45^neg^ erythroid precursor cells in the Spl (Supplementary Fig. 15j, k), demonstrating that acute Notch2 blockade is sufficient to induce the extramedullary erythropoiesis  phenotype observed in the *N2*^*∆Cx3cr1*^ model.

The mouse Spl sustains an active erythropoietic activity from birth that gradually ceases around 7 weeks of age when the BM establishes itself as the main erythropoietic organ^42^. Independent of genotype, we saw abundant, proliferating CD71^+^ erythropoietic cells in 2 wk-old mice (Fig. 7f, g). In Ctrl mice, these cells formed clusters of isolated erythropoietic islands surrounded by F4/80^+^ RPM, but in *N2*^*∆Cx3cr1*^ mice, they were distributed throughout the RP. In 10 wk-old mice, proliferation was absent regardless of genotype, but in contrast to Ctrl mice, *N2*^*∆Cx3cr1*^ mice maintained abundant clusters of CD71^+^ erythropoietic islands (Fig. 7h, i). Additional staining revealed smaller, non-nucleated CD71^+^Ter119^+^ double-positive early erythrocytes in proximity to the islands (Fig. 7j). Furthermore, in *N2*^*∆Cx3cr1*^ mice, CD71^+^ erythropoietic islands were surrounded by CD169^+^ cells, while in Ctrl mice, Ter119^+^ erythrocytes were scattered in between F4/80^+^ RPM in the RP (Fig. 7j and Supplementary Fig. 16a–c). Interestingly, despite the rescue of RPM in *N2*^*∆Cx3cr1*^ mice after repopulation with BM or FL cells (Fig. 6 and Supplementary Fig. 14), CD71^+^ cell islands remained in the Spl (Fig. 7k and Supplementary Fig. 16d).

To test if active splenic erythropoiesis is responsible for the altered splenic architecture in *N2*^*∆Cx3cr1*^ mice (Fig. 4a, Supplementary Video 1, 2), we performed phlebotomy and analyzed Spl 5 days after using CLSM. We found no alterations in the RP and CD169^+^ MMM ring of phlebotomized Ctrl mice despite active splenic erythropoiesis (Supplementary Fig. 16e, f). These data suggest that the loss of RPM and the CD169^+^ MMM ring structure in *N2*^*∆Cx3cr1*^ mice is independent of splenic erythropoiesis and is due to *Notch2* deletion.

### *Notch2*-deficiency is associated with an altered early response to PHZ-induced hemolytic anemia

**Fig. 8:**
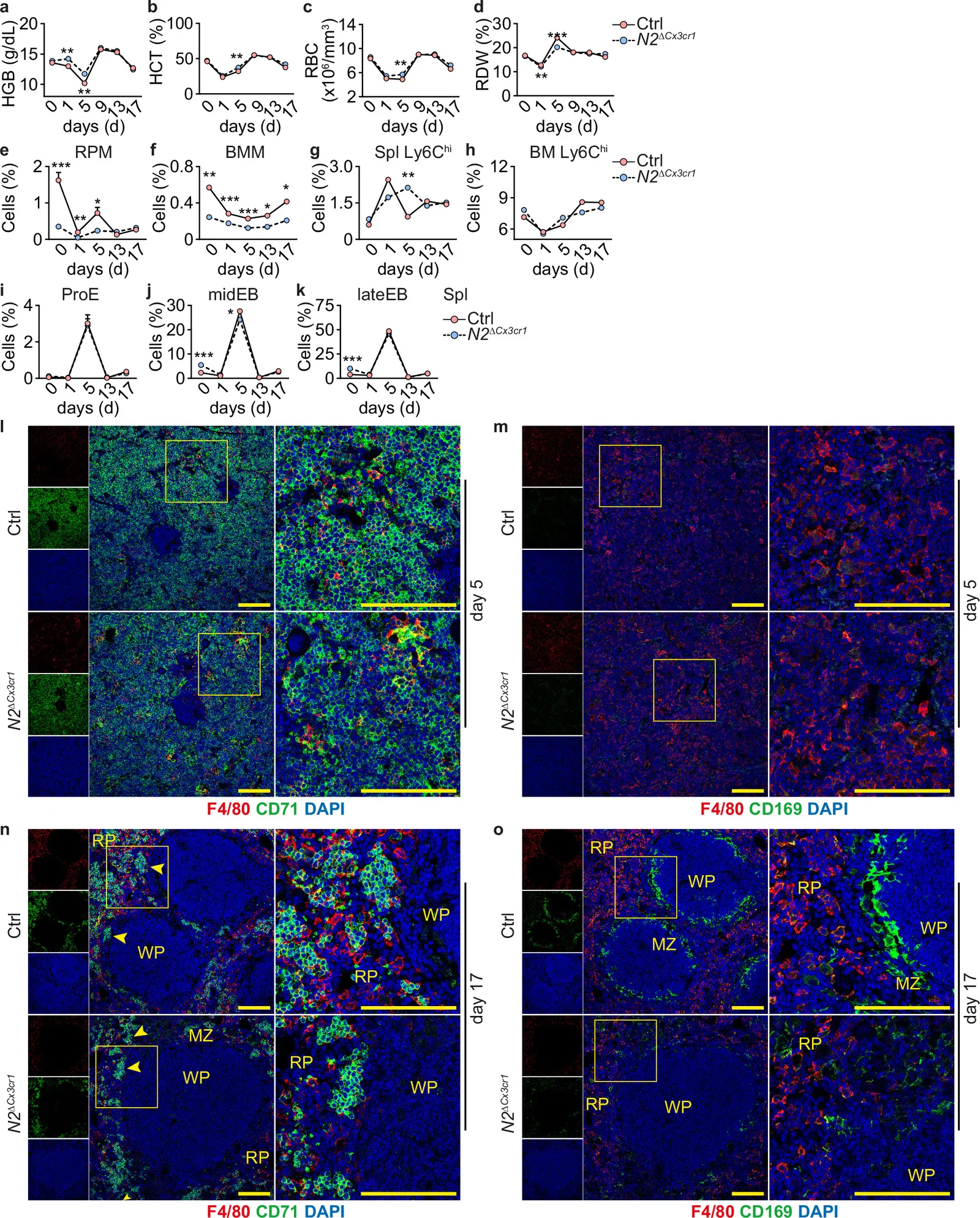
*Notch2*-deficiency is associated with an altered early response to PHZ-induced hemolytic anemia. **a**–**d** Blood count parameters in Ctrl and *N2*^*ΔCx3cr1*^ mice injected with PHZ. **e**–**h** Relative frequency (from live cell gate) of (**e**) RPM, (**f**) BMM, **g** Spl Ly6C^hi^ monocytes, and (**h**) BM Ly6C^hi^ monocytes in PHZ-treated mice. **i**–**k** Relative frequency (from live cell gate) of Spl erythroid precursors in PHZ-treated mice. **l**–**o** CLSM images of Spl on day 5 (**l**, **m**) or 17 (**n**, **o**) after PHZ-injection. Representative of two (**l**, **m**) and three (**n**, **o**) experiments. Arrowheads depict erythropoietic clusters. Scale bar 100 µm. (**l**, **m**) Images are from the same samples but depicted separately for simplicity. (**n**, **o**) Images are from the same samples but depicted separately for simplicity. **a**–**k** Data are mean ± SEM. *P < 0.05; **P < 0.01; ***P < 0.001; (unpaired two-tailed Student’s *t* test). Each time point pooled from three experiments except (**a**–**d**) where 0 d is pooled from five experiments. Exact n numbers are provided in Source Data. Source data are provided as a Source Data file.

To test whether *Notch2* deletion alters the response to hemolytic anemia, we induced hemolysis through phenylhydrazine (PHZ) administration and analyzed PB and organ parameters (Supplementary Fig. 17a–n). Following PHZ, hemoglobin (HGB) levels decreased significantly faster and more pronounced in Ctrl than in *N2*^*ΔCx3cr1*^ mice in the early response period, while recovery of hematocrit (HCT) and RBC counts was weaker at d5 (Fig. 8a–d and Supplementary Fig. 17c–n). Mice also developed splenomegaly, which at its peak was not different between genotypes (Supplementary Fig. 17b). This observation indicates better adaptation to hemolytic stress in *Notch2*-deficient mice in the initial response to anemia.

In Ctrl mice, PHZ administration led to a strong and persistent reduction in RPM numbers, which only transiently and partially recovered (Fig. 8e and Supplementary Fig. 17o). BMM frequency also decreased after PHZ but partially recovered during the late phase (Fig. 8f and Supplementary Fig. 17p). This was in stark contrast to *N2*^*ΔCx3cr1*^ animals, which showed strongly reduced RPM and BMM frequencies at baseline and during follow up (Fig. 8e, f, Supplementary Fig. 17o, p). Concomitantly, the number of Ly6C^hi^ monocytes on day 1 transiently increased in Spl and decreased in BM, preceding an increase of RPM at day 5, suggesting recruitment and active differentiation of monocytes into RPM, as described by Haldar et al.^17^ (Fig. 8g, h and Supplementary Fig. 17q, r). This did not occur in *N2*^*ΔCx3cr1*^ animals, where Ly6C^hi^ monocytes remained elevated in parallel to low numbers of RPM (Fig. 8e, g). Thus, the defects in RPM and BMM observed in *N2*^*ΔCx3cr1*^ mice are pronounced and persistent.

In response to PHZ, there was a strong increase in splenic early erythrocyte precursors on day 5, which was comparable between Ctrl and *N2*^*ΔCx3cr1*^ mice with a modest difference in midEB frequencies (Fig. 8i–k). A similar increase was observed on day 5 in BM and liver accompanied by increased number of reticulocytes in PB (Supplementary Fig. 17s–y). Approximately 9–17 days post PHZ administration, erythroid parameters in PB mainly returned to baseline levels (Fig. 8a–d). This coincided with decreased number of erythroid precursors in the Spl, BM and liver, likely due to differentiation into mature erythrocytes (Fig. 8i–k, Supplementary Fig. 17s–y). Despite normalization of erythroid parameters 17 days after PHZ administration, the cellularity in both the Spl and BM continued to exhibit aberrations, with low levels of RPM and BMM in both Ctrl and knockout animals (Fig. 8e, f). This may suggest that rapid generation of new RBC has priority over the recovery of ablated RPM and BMM, which occur at a much slower rate.

This was also reflected in the cellular and spatial changes in the Spl by CLSM. On day 5 post-PHZ administration, the Spl lost the characteristic architecture (Fig. 8l, m), and evidence for an active erythropoietic state, seen by the presence of CD71^+^ erythroblastic islands in RP, persisted until day 17 independent of genotype (Fig. 8n, o). At the late recovery phase, the Spl of Ctrl mice started to return to the normal structure where distinct RP, MZ and WP were visible (Fig. 8n, o). Notably in Ctrl mice, usual CD169^+^ MMM rings and F4/80 RPM were observed, while *N2*^*ΔCx3cr1*^ Spl showed the typical phenotype with lack of distinct MMM ring and CD169^+^ cells diffused in RP (Fig. 8o). Taken together, our data suggests that the enhanced erythropoietic activity in the *N2*^*ΔCx3cr1*^ animals may provide an altered initial response following PHZ-induced anemia. Moreover, an intact Notch2 is essential for the restoration of the typical splenic structure and recovery of the BMM population following hemolysis.

## Discussion

Here, we show that canonical Notch2 signaling is essential for the development and maintenance of iron-recycling TRM, as summarized in Supplementary Fig. 18. Mice with loss of *Notch2* and its downstream mediator *Rbpj* displayed defects in RPM and BMM populations and iron overload in the Spl and BM, while prototypic RPM genes required for iron handling were downregulated. This was accompanied by splenic extramedullary erythropoiesis, expansion of CD163-expressing monocytes and CD169^+^ cells and changes in splenic microarchitecture. Furthermore, defects in RPM and BMM and iron overload were rescued by early postnatal transfer of bone marrow and fetal liver progenitors in a *Notch2*-dependent manner, demonstrating the potential to restore defective TRM niches by Notch-competent progenitors.

Analogous to reports from *Spic-*^18^ and *Pparg*-deficient mice^21^, deletion of *Notch2* led to RPM and BMM reduction, suggesting the possibility that *Spic*, *Pparg* and *Notch2* may operate within a shared or overlapping pathway regulating development and function of these macrophages. It may also indicate that Notch2 regulates the numbers or differentiation of a potential shared progenitor, which could explain the selective macrophage loss in these specific organs. On the other hand, our findings that antibody-mediated blockade of Notch2 receptors in adult wt mice results in a subsequent reduction of RPM indicates a requirement for Notch2 in the maintenance of an established erythrophagocyte pool, thereby adding an additional layer of complexity to the regulation of TRM.

Although it is widely accepted that the majority of TRM originate from distinct fetal progenitors during embryonic and fetal liver developmental waves^66,67^, evidence for the precise progenitor of RPM and BMM is scarce. In addition, niche-specific signals, derived from the microenvironment, play a crucial role in shaping the identity and function of macrophages, regardless of origin^68^. Defective TRM pools can be reconstituted by transfer of FL or BM cells in newborn mice supporting niche-mediated instruction of macrophage development^21,68,69^. Our studies demonstrate that BMM and RPM in *Notch2*-deficient newborn mice are reconstituted by FL or BM cells in a *Notch2*-dependent manner. Several lines of evidence suggest a unique, but possible shared origin and developmental pathway of RPM and BMM, as was proposed by Haldar et al.^17^: (1) the isolated defect of RPM and BMM in *Notch2*-mutant mice not affecting other TRM (such as KC in the liver), (2) the localized iron deposition in their resident niche, (3) the fact that both populations are reconstituted via BM or FL cell transfer.

On the other hand, induced reporter studies indicate that under steady-state conditions majority of RPM and BMM are not developed, or *Ms4a3* or *Cx3cr1* are not expressed at the time point of pulsing (P5-P7). In contrast, in constitutive reporter models, only 10% of RPM but 50% of BMM show a GMP-origin. The complex spatial and temporal overlaps, combined with the lack of appropriate tools, make it challenging to precisely determine the timing and extent to which *Notch2* may influence RPM or BMM development. Similarly, the extent to which BM- or FL-derived RPM or BMM are transcriptionally equivalent to their bona fide embryonic-derived counterparts is not addressed in this study and requires further investigation. However, independent of the origin or developmental kinetics, Notch2 is critically important for the formation and maintenance of proper RPM and BMM pools.

The principal scavenger receptor for extracellular hemoglobin is CD163^70^, which, unlike humans, is expressed on murine macrophages, including RPM, but not on circulating monocytes^71,72^. The observed expansion of CD163^+^ monocytes locally in *N2*^*ΔCx3cr1*^ spleen may reflect a compensatory response to RPM loss. Consistent with this, anti-Notch2 treatment likewise induced expansion of this population, suggesting a Notch2-dependent consequence of RPM pool disruption.

Interestingly, although changes in CD163 expression in *N2*^*ΔCx3cr1*^ Ly6C^lo^ monocytes and RPM was supported by both flow cytometry and gene expression analyses (qRT-PCR), *Cd163* was not identified as a significantly differentially expressed gene in the corresponding scRNA-seq analysis in *N2*^*ΔCx3cr1*^ mice. This discrepancy likely reflects differences in sensitivity and resolution between different experimental approaches.

Our data also demonstrate that the normal splenic architecture was disrupted in *Notch2*-deficient mice. In the absence of RPM, the CD169^+^ MMM ring forming the border between white and red pulp was missing and the expanding pool of CD169^+^ cells accumulated in the RP, partially resembling results from LXRα-deficient (Liver-X Receptor alpha, encoded by *Nr1h3*) mice, which lack splenic MZ macrophages, including MMM^73^. LXRα-deficient mice, however, retain a normal RPM pool with no signs of iron deposits. The structural loss of the CD169^+^ MMM ring in *N2*^*ΔCx3cr1*^ mice suggests that LXRα signaling might also be regulated via *Notch2*. Indeed, we saw a reduced expression of *Nr1h3* in the absence of *Notch2* using an in vitro model mimicking macrophage differentiation. Moreover, reconstitution of *N2*^*ΔCx3cr1*^ mice with Ctrl BM monocytes rescued the CD169^+^ MMM ring defect, similar to BM reconstitution studies in LXRα-deficient mice^73^. Alternatively, loss of RPM may simply allow CD169^+^ cells to expand and translocate into the RP, indicative of either a compensatory mechanism to RPM loss or altered signaling in this cellular subset due to the lack of *Notch2*. The mechanism and the functional significance of such translocation remain to be explored.

DLL4-Notch-RBPJ signaling induces the expression of LXRα and Spi-C in BM monocytes and enables their differentiation into KC^31,32^. In the spleen, the ligand DLL1 is prominently expressed by endothelial cells in the MZ and regulates splenic MZ B-cell development but also monocyte conversion in a *Notch2*-dependent manner^25,74^. Indeed, *Dll1* is highly expressed in splenic capillary EC^75^, and recent work^76^ has also identified DLL1 on splenic fibroblasts as a physiologically relevant ligand for Notch2, while *Dll4* expression is limited to capillary arterial EC^75^. These Dll1-expressing EC and/or splenic fibroblasts could regulate the differentiation of potential precursors into RPM, similar to the process observed for Dll4^31^, and this may be contingent on *Notch2*. In line with these data, our in vitro culture experiments show *Notch2*-dependent upregulation of *Nr1h3*, *Spic*, and *Slc40a1* in the presence of DLL1, which was further enhanced by hemin. This suggests that DLL1-Notch2 and Heme-Bach1 pathways closely interact to promote the development of macrophages.

In the spleen, the myeloid growth factor CSF-1 is provided by WT1^+^ RP reticular fibroblasts, which is required for RPM maintenance^16^, while the CSF-1 receptor antagonist GW2580 reduced the number of RPM in mice, supporting a role for CSF-1 in RPM survival^36^. IL-33 has also been identified as a regulator of RPM development^20^. While it is evident that Notch, CSF-1, and IL-33 signaling axes are needed for the formation or maintenance of RPM, the link between these pathways remains to be established.

The neonatal Spl contains CD71-expressing erythroid precursors and is an active site of erythropoiesis but these precursor numbers gradually decrease, and in adult mice, the BM becomes the primary site of erythropoiesis^42^. In adult *N2*^*ΔCx3cr1*^ mice, we found signs of iron overload and persistence of splenic erythroid precursors, indicative of extramedullary and compensatory erythropoiesis, since no PB count abnormalities were noted. This may further be promoted by increased splenic iron levels, which enhances the erythropoietic potential by maintaining erythroid progenitors and promoting their differentiation into mature erythrocytes^77^. Consistent with this, anti-Notch2 antibody treatment similarly induced extramedullary erythropoiesis in wt mice, directly linking this phenotype to impaired Notch2 signaling and as a consequence of RPM loss. Given the central role of RPM in iron recycling, their loss leads to impaired iron handling and likely to the alteration of certain niche-derived signals, promoting or inducing sustained splenic erythropoiesis.

Furthermore, it has been shown that loss of CD169^+^ BMM facilitates the egress of HSPC from the BM into circulation^78^. A similar mechanism involving BMM loss may potentially explain the changes seen in *N2*^*ΔCx3cr1*^ mice, specifically the increased numbers of erythroid precursors in the *N2*^*ΔCx3cr1*^ Spl. However, the lack of Ki-67 staining within the CD71^+^ islands in adult mice might suggest an HSPC proliferation tropism to the BM before egress to the spleen. Thus, Notch2-dependent changes in the BMM and RPM likely shift or promote erythropoiesis to the spleen.

We propose that maintenance of high numbers of erythroblasts in the RP may offer a protective advantage during the initial phase of acute hemolytic anemia. This may have significant implications for traumatic blood loss, where early mitigation could substantially influence recovery outcomes. Nevertheless, *N2*^*ΔCx3cr1*^ mice are still able to recover from hemolysis, indicating that other key regulators beyond RPM or BMM are efficiently involved in restoring homeostasis. However, it still remains to be explored whether the loss of RPM and BMM with the associated structural changes might have an influence on iron homeostasis and erythropoiesis in iron deficiency conditions or on responses to infection. A recent study^4^ has directly linked RPM to the clearance of *Streptococcus pneumoniae*, wherein the absence of RPM had detrimental effects on recovery after infection. Given their use of *Spic*-deficient mice to elucidate this, it is plausible that *N2*^*ΔCx3cr1*^ mice may exhibit somewhat similar responses.

Limitations: Cx3cr1 is broadly expressed throughout the myeloid lineage, both during embryonal development and postnatal period. Thus, constitutive *Cx3cr1*^*Cre*^-based targeting is not able to identify the exact origin of macrophage progenitors. For this aim, new, more precise tools are needed to selectively target specific progenitors at different stages of development.

Additionally, the BM contains multiple macrophage subsets that are not yet well characterized. In this study, BMM were defined based on the currently accepted phenotypic markers. Consequently, while our data demonstrate a reduction in F4/80^+^ BMM populations upon Notch2 deletion, additional macrophage subsets in the BM that were not analyzed, might also contribute to the iron-related phenotypes observed in *N2*^*ΔCx3cr1*^ mice.

A further limitation is that the specific Notch2 ligand and its cellular source, potentially responsible for the development and maintenance of erythrophagocytes, were not directly examined. Similarly, the precise location within the microenvironment where such ligand–receptor interactions occur and contribute to the formation or the proper establishment of the TRM pool, remains unknown.

Our findings reveal a previously undescribed role of canonical Notch2 signaling in the formation, maintenance and function of RPM and BMM pools. These results highlight the indispensable function of *Notch2* for iron-recycling macrophages, iron homeostasis and erythropoiesis, which affects splenic structure.

## Methods

### Mice

Adult mice (8–16 weeks old) with age and sex-matched controls were used in experiments, unless otherwise indicated. B6.SJL*-Ptprc*^*a*^*Pepc*^*b*^*/*BoyJ (CD45.1^+^) mice were from the central animal facility of Hannover Medical School (ZTL, MHH). Wt mice (C57BL/6NRj) were purchased from Janvier-Labs. *Cx3cr1*^*Cre*^ (B6.Cg-*Cx3cr1*^*tm1.1(cre)Jung*^)^38^, *Cx3cr1*^*GFP/+*^ (B6.129P-*Cx3cr1*^*tm1Litt*^/J, JAX stock #005582)^43^, *Notch2*^*lox/lox*^ (B6.129-*Notch2*^*tm1Frad/J*^)^39^, *Rbpj*^*lox/lox*^ (B6.129*-Rbpj*^*tm1Hon*^)^40^, *ROSA26-mT/mG* (*Gt(ROSA)26Sor*^*tm4(ACTB-tdTomato,-EGFP)Luo*^*/J*, JAX stock #007576)^61^, *Ms4a3*^*CreERT2*^*Rosa*^*LSL-tdT*^^79^, *Ms4a3*^*Cre*^*Rosa*^*LSL-tdT*^^79^, *Cx3cr1*^*CreERT2*^^38^ (JAX stock #020940) and *Rosa*^*LSL-YFP*^^80^ (JAX stock #006148) mice have been previously described. *N2*^*ΔCx3cr1*^ (B6.Cg-*Cx3cr1*^*tm1.1(cre)Jung*^ *Notch2*^*tm1Frad*^) and *Rbpj*^*ΔCx3cr1*^ (B6.Cg-*Cx3cr1*^*tm1.1(cre)Jung*^ *Rbpj*^*tm1Hon*^) ^41^ mice were generated by crossing *Cx3cr1*^*Cre*^ mice^38^ with *Notch2*^*lox/lox*^^39^ or *Rbpj*^*lox/lox*^ animals^40^ respectively. *N2*^*∆Cx3cr1*^ *mG* (B6.Cg-*Cx3cr1*^*tm1.1(cre)Jung*^ *Notch2*^*tm1Frad*^ *Gt(ROSA)26Sor*^*tm4(ACTB-tdTomato,-EGFP)Luo*^) and Ctrl *mG* (B6.Cg-*Cx3cr1*^*tm1.1(cre)Jung*^ *Gt(ROSA)26Sor*^*tm4(ACTB-tdTomato,-EGFP)Luo*^) strains were generated by crossing *ROSA26-mT/mG* mice^61^ with *Cx3cr1*^*Cre*^*Notch2*^*lox/lox*^ *(N2*^*ΔCx3cr1*^) or *Cx3cr1*^*Cre*^*Notch2*^*+/+*^ (Ctrl) animals, respectively. *Cx3cr1*^*CreERT2*^*Rosa*^*LSL-YFP*^ (*Cx3cr1*^*iYFP*^*)* were generated by crossing *Cx3cr1*^*CreERT2*^^38^ with *Rosa*^*LSL-YFP*^^80^ mice. Mice were bred and housed under specific pathogen-free conditions at a 14:10 h light: dark cycle at 22 °C ± 2 °C and 55% ± 5% relative humidity in rodent areas in the central animal facility of Hannover Medical School (ZTL, MHH) or in the central animal facility of the University of Bonn.

Mice were euthanized under deep anesthesia using a mixture of Ketanest, Rompun and Dormicum as approved by the local animal welfare board (LAVES, Lower Saxony, Germany). Newborn mice (P0-1wk old) and embryos were euthanized by decapitation.

All experiments were approved by the local animal welfare board (LAVES, Lower Saxony, Germany). The majority of the experiments were performed with female mice with the exception of repopulation studies and experiments involving P0 and P1 mice, where both male and female recipients were analyzed. Experiments with *Ms4a3*^*CreERT2*^*Rosa*^*LSL-tdT*^, *Ms4a3*^*Cre*^*Rosa*^*LSL-tdT*^ and *Cx3cr1*^*iYFP*^ were approved by the local animal welfare board (LANUV, Landesamt für Natur, Umwelt und Verbraucherschutz) in North Rhine-Westphalia, Germany.

### Tissue and cell preparation

To prepare single-cell suspensions, mice were euthanized and organs were collected as previously described^37^. Briefly, Spl were mechanically dissociated, BM was collected by centrifugation and PB was collected from the inferior vena cava. Livers were digested at 37 °C for ~1 h in DMEM supplemented with 500 U/ml Collagenase II (Worthington). Erythrocytes were excluded using red blood cell lysis buffer (BioLegend) or by density centrifugation using Histopaque 1083 (Sigma-Aldrich). Cells were thoroughly washed and resuspended in PBS containing 10% fetal calf serum (FCS) and 2 mM EDTA and used for flow cytometry. For splenic macrophage subset analysis, spleens were digested on ice for 30 min. in DMEM supplemented with 500 U/ml Collagenase II (Worthington), 1.5 U/ml DNAse (Sigma-Aldrich) and 2% FCS.

### Flow cytometry and cell sorting

To reduce non-specific binding of antibodies to Fc-receptors in single-cell suspensions prepared from Spl, PB, BM or Liver, anti-mouse CD16.2 (FcγRIV) and anti-mouse CD16/32 (TruStain FcX™) blocking antibodies were used. Cells were then thoroughly washed, stained with primary and secondary antibodies or streptavidin-fluorochrome conjugates and subjected to flow cytometry (LSR II, BD Biosciences) or sorting (FACSAria III Fusion, FACSAria IIu, BD Biosciences). All Antibodies used in flow cytometry and cell sorting are described in Supplementary Table 2. All flow cytometry data were analyzed using FlowJo software (FlowJo LLC).

FSC and SSC parameters were used to identify the cells. After doublet exclusion (based on SSC-W and SSC-A parameters), the relative frequency of the cells (calculated from live cell gate, PI^neg^) or absolute numbers of each subset (normalized per Spl, per mg BM or per µl PB) was determined and is shown as mean ± SEM in the figures, unless otherwise stated. Unsupervised t-distributed stochastic neighbor embedding (t-SNE)^81^ and clustering analysis with PhenoGraph^49^ (plugin functions in FlowJo software) was performed on live CD45^+^Lin^neg^CD117^neg^ population after exclusion of CD11b/CD11c double negative cells in concatenated samples. Values shown in representative flow cytometry plots represent the frequency of the parent population within the parent gate, as calculated by the FlowJo.

The deletion efficiency of Notch2 in splenic and BM populations was calculated from the reduction in Notch2^+^ cells in *N2*^*ΔCx3cr1*^ relative to Ctrl mice after staining and flow-cytometry analysis.

### Apoptosis and proliferation assay

Apoptosis assay was performed according to the manufacturer’s instructions (Biolegend). Briefly, single-cell suspensions were stained with primary and secondary antibodies or streptavidin-fluorochrome conjugates, washed and resuspended in AnnexinV (AnnV) binding buffer (Biolegend). Cells were stained with AnnV at room temperature for 20 min. After incubation, propidium iodide (PI) (Sigma-Aldrich) was added and cells were immediately analyzed by flow cytometry.

BrdU incorporation assay was performed according to the manufacturer’s instructions (BD Biosciences). Briefly, 30 µl BrdU (10 mg/ml in sterile PBS) was administered to neonates (P5-P7) intraperitoneally (i.p.). The animals were sacrificed the next day. Single cell suspensions were stained with surface antibodies, fixed-permeabilized, stained with anti-BrdU and analyzed using flow cytometry.

### Immunohistochemistry and microscopy

Organs were harvested from euthanized mice, fixed in 4% PFA in PBS and embedded in optimum cutting temperature (OCT) compound. 8 µm thick cryosections were prepared using a cryostat (Leica CM3050S) and stored at −20 °C. Sections were blocked with anti-mouse CD16/CD32 (TruStain fcX from BioLegend) in 3% BSA and stained with appropriate primary and secondary antibodies. Sections were counterstained with DAPI. Images were taken using 20x oil objective on Leica Inverted 3 TCS SP8 DMi8 microscope.

For confocal imaging of cell populations, sorted cells were washed, fixed-permeabilized, placed on poly-L-lysine-coated glass coverslips and allowed to air dry. Coverslips were mounted on glass slides and imaged using a 63x oil objective on Leica Inverted 3 TCS SP8 DMi8 microscope. Confocal images of sections and sorted cells were processed with Leica Application Suite (LAS) AF Lite software (Leica).

For 3D reconstruction, 200 µm thick sections were processed and stained using the Ce3D™ Tissue Clearing Kit (Biolegend) according to the manufacturer’s instructions. Z-stack images were acquired using a Leica Inverted 3 TCS SP8 DMi8 microscope. Image processing and video generation were done using Imaris x64 v8.2.1.

Cytospins of sorted cells were stained with a modified Wright-Giemsa staining solution. Multiple images of each biological replicate were taken using a 63x oil objective on a Zeiss Axioplan 2 Imaging microscope with Zeiss Axiocam 208 color camera and Labscope software.

ImageJ was used for quantification of the RPM in the Spl. In brief, five different areas were randomly chosen in the red pulp. Each area was thresholded to distinguish RPM from background and % of F4/80^+^ area was calculated. Separately, intensity of F4/80 signal (F4/80 expression) was measured as the average grayscale brightness of the area, calculated from RGB values after conversion to 8-bit.

### Prussian blue iron staining

Ferric iron deposits were visualized using Perl’s Prussian blue stain in frozen tissue sections and counterstained with nuclear red. Multiple images of each biological replicate were taken using 10x and 20x objectives of Leica DMI 3000B DFC420C microscope or Zeiss Axioplan 2 Imaging microscope with Zeiss Axiocam 208 color camera. Images were acquired and processed using LAS v3 (Leica) or Labscope software.

### Iron quantification

Iron quantification was done using a Ferrozine-based method^82^ with modifications. Briefly, homogenizing buffer (HB) was prepared by dissolving sodium chloride (Roth) in NaOH-HEPES (pH 7.4, Sigma-Aldrich) at final concentrations of 0.15 M and 10 mM, respectively. Extraction buffer (EB) was prepared by combining 50% trichloroacetic acid (Sigma-Aldrich) and 8% sodium pyrophosphate tetrabasic (Sigma-Aldrich) and kept at 4 °C. Ferrozine solution (FS) was freshly prepared by combining 38 mM ascorbic acid (Sigma-Aldrich), 1.3 mM ferrozine (Sigma-Aldrich) and 1.4 M (pH 4.8) sodium acetate (Sigma-Aldrich). For Fe standards, a stock concentration of 1 mM FeCl_3_ (Sigma-Aldrich) was prepared in 10 mM HCl (Sigma-Aldrich). Standard curves were generated at a FeCl_3_ starting concentration of 100 µM.

Organs were manually disrupted/dissolved in 300 µl HB, then mixed with EB (at 1:1 ratio) and digested at 95 °C for 20 min. After centrifugation at 17,000 × *g* for 10 min., 25 µl of the supernatant was mixed with 75 µl FS in 96 half well flat bottom plates (Greiner) and absorbance was measured at 562 nm (Infinite® 200, Tecan). Each sample was analyzed in duplicates and the average value was used for calculation of Fe concentration per mg tissue or per µl blood.

### Phlebotomy

For induction of anemia, 400 µl of blood was removed from mice via retro-orbital puncture under isoflurane anesthesia. The blood loss was restituted immediately with i.p. injection of 400 µl NaCl solution.

### Adoptive transfer to neonates

BM or FL cells were harvested from 8–10 weeks old or E14.5 embryo donor mice, respectively. Single cell suspension was prepared in PBS. 8 × 10^6^ donor cells were injected once i.p. to neonates (P5-P7). In a separate experiment, BM Ly6C^hi^ monocytes were sorted from adult mice and used for reconstitution experiment. Recipient mice were euthanized 8–14 weeks later and reconstitution was analyzed using flow cytometry and confocal microscopy.

### Cell hashing and single-cell RNA sequencing

#### Cell hashing and sorting

Ctrl, *N2*^*ΔCx3cr1*^ and *Rbpj*^*ΔCx3cr1*^ Spl (n = 2 per genotype) were isolated and digested on ice for 30 min. in DMEM medium supplemented with 500 U/ml Collagenase II (Worthington) and 2% FCS. Erythrocytes were removed using red blood cell lysis buffer (BioLegend). Following thorough washing, filtering and counting, single cell suspensions of equal cell numbers (one tube per Spl) were prepared. For sample multiplexing, a cell hashing-based approach was performed using oligo-tagged antibodies (TotalSeq^TM^-A Hashtag derived Oligos, HTO, #A1-#A6, Biolegend). Blocking was done with anti-mouse CD16/32 (TruStain fcX) and anti-mouse CD16.2, followed by HTO tagging (#A1-#A6) and anti-F4/80-BV650 staining. After thorough washing, cells were pooled and stained with Lin-PE antibodies (CD3, CD19, B220, Ly6G, Ter119, NK1.1, CD90.2, CD49b, CD117 and FcεRIα and anti-mouse CD11b-FITC, CD11c-APC and Ly6C-PE-Cy7. Following the exclusion of dead (DAPI^+^) and CD11b/CD11c double-negative cells, Lin^neg^ cells were sorted using FACSAria Fusion (BD Biosciences). Collected cells were loaded on one 10x Genomics lane for downstream scRNA-seq processing and analysis.

#### Preparation of library and sequencing run

The library was prepared according to Chromium NextGEM Single Cell 3’ Reagend Kits v3.1 CellSurfaceProtein User Guide (Manual Part Number CG000206 Rev D; 10x Genomics). Briefly, a specified excess of cells was loaded to the 10x controller to achieve the target count of 25000 cells. The fragment length distribution of the library was assessed using Bioanalyzer High Sensitivity DNA Assay (Agilent Technologies). Library quantification was carried out using Qubit® dsDNA HS Assay Kit (Thermofisher Scientific). The generated mRNA expression libraries were pooled, denatured with NaOH and diluted to achieve 1.8 pM or 2 pM following the outlined guidelines in Denature and Dilute Libraries Guide (Document #15048776 v02 from Illumina). A 1.3 ml aliquot of the denatured pool (with 1% PhiX) was sequenced on an Illumina NextSeq550 sequencer using a single high output flowcell for 75 cycles and 400 million clusters (#20024906, Illumina). The HTO libraries were loaded at a molar proportion that corresponds to ~5% of the sequencing run capacity. Sequencing parameters were as follows: Sequence read 1: 28 bp; Sequence read 2: 56 bp; Index read 1: 8 bp; Index read 2: none.

#### Data processing and analysis

The 10x Genomics CellRanger pipeline set (v7.10) was run with default settings. BCL files were demultiplexed into Fastq files using cellranger mkfastq, through the use of the 10x barcodes and respective sample sheets. Fastq files from the sequencing runs were pooled. The cellranger count pipeline aligned the reads to the Mus musculus reference genome (refdata-gex-mm10-2020-A, provided by 10x Genomics), counted reads per gene and generated summary statistics. Resulting outputs from cellranger count were aggregated, normalized to the same sequencing depth and the feature-barcode matrix was recomputed using cellranger aggr. During the cellranger count step, feature barcoding data was processed using a feature reference build based on TotalSeq™-A antibodies specifications.

The resulting HDF5 file (‘filtered_feature_bc_matrix’), which included data from all genotypes and HTOs (12384 cells), was imported into SeqGeq for normalization and multistep quality control filtering prior to downstream analyses.

Reads were normalized to counts per 10000 using all genes excluding HTOs. Initial quality control was performed in SeqGeq by manual gating (cell filter) on library size versus genes expressed. Cells expressing less than 300 genes and/or falling outside the main population, corresponding to empty wells, noise, debris, and doublets, were excluded, yielding a dataset of 11948 cells (“Quality cells”). Next, gene-level filtering was performed in SeqGeq Gene View by excluding genes detected in fewer than 5 cells as well as genes expressed by all cells, identifying 11723 “Quality genes”.

A synthetic parameter representing the summed expression of mitochondrial genes was generated in SeqGeq (Mito-Sum) from the “Quality cells” population. Cells with high mitochondrial gene scores were identified by manual gating and excluded from downstream analyses, yielding dataset of 10102 cells (“Live cells”).

HTO-based demultiplexing was performed on “Live cells” by manual gating of individual HTO expression distributions. Cells exhibiting HTO signal above predefined positivity threshold (10^1^) was assigned to the corresponding sample. To exclude doublets, each HTO-positive population was sequentially assessed for positivity of remaining HTOs. Cells positive for more than one HTO were excluded. This step yielded 9179 cells that were used for downstream analyses.

Following downsampling, dimensionality reduction was performed in SeqGeq using a combination of principal component analysis, t-SNE and clustering using PhenoGraph^49^. Known marker genes for different myeloid cell populations were used to annotate and characterize the resulting clusters^17,51–54^. Visualization of signature gene expression using bubble heatmap was done using the Violin Box Plug-in^83^ from SeqGeq.

Cell clusters of interest were pooled by population for downstream differentially expressed genes (DEGs) and exploratory enrichment analyses. DEGs between Ctrl and *N2*^*∆Cx3cr1*^ or *Rbpj*^*∆Cx3cr1*^ cells was assessed using conventional cell-level statistics (Mann-Whitney U-test) in the SeqGeq program. *P*-values were adjusted using the Benjamini-Hochberg false discovery rate (FDR; *q* value) method in SeqGeq. Genes with *q* value < 0.05 and log_2_ fold change ≥0.3 or ≤ -0.3 were considered differentially expressed. Exploratory enrichment analysis was performed using Enrichr^55–57^ with Molecular Signatures Database (MSigDB) Hallmark^58^ and Gene Ontology (GO) Biological Process gene sets^59,60^. Commonly upregulated and downregulated genes were defined as those meeting DEG criteria.

### In vitro culture studies

24-well plates were coated at room temperature for 2-3 h with IgG-Fc or DLL1-Fc ligands (all from R and D) reconstituted in PBS. Sorted BM Ly6C^hi^ monocytes were cultured for four days in coated plates in the presence of CSF-1 (10 ng/ml, Peprotech) at 37 °C. Cells were stimulated on day 2 with 40 µM porcine Hemin (Sigma-Aldrich) reconstituted in 2 mM NaOH (Sigma). Cultured cells were harvested and isolated RNA was used for gene expression analysis (qRT-PCR).

### In vitro phagocytosis assay

Murine erythrocytes (RBC) were stained with carboxyfluorescein succinimidyl ester (CFSE) following a modified protocol^84,85^. Briefly, following depletion of CD45^+^ cells, RBC were stained with 2.5 µM CFSE. After thorough washing steps, a fraction of the cells was heat-stressed at 48 °C for 30 min. Non-stressed RBC were incubated at 4 °C for 30 min. Splenocytes from Ctrl or *N2*^*∆Cx3cr1*^ mice were isolated and stained with the appropriate antibodies as described in the manuscript. Cells were coincubated with CFSE-labelled stressed or non-stressed RBCs at a 1:10 ratio for 1 h at 37 °C. Following brief lysis of remaining RBC using RBC-lysis buffer and extensive washing steps, Ctrl and *N2*^*∆Cx3cr1*^ splenocytes were subjected to flow cytometry. Phagocytic activity was determined by measuring the frequency of CSFE^+^ RPM or monocytes and the mean fluorescence intensity (MFI) of CFSE in these cells.

### Detection of Notch2 recombination using PCR

Polymerase chain reaction (PCR) was performed to amplify the floxed and recombinant regions of Notch 2 in DNA isolated from sorted cells using locus-specific primer pairs. PCR products were analyzed using agarose gel electrophoresis.

Primer sequences are as follows:

*Notch2* deletion forward: 5’-GTC GCT GTT GTC ATC ATC-3’

*Notch2* deletion reverse: 5’-GTG CAC ATA TGC CTT AGC-3’

*Notch2 floxed* forward: 5’-GAG AAG CAG AGA TGA GCA GAT G-3’

*Notch2 floxed* reverse: 5’-GTG AGA TGT GAC ACT TCT GAG C-3’

### RNA isolation and quantitative real-time PCR

Purification of total RNA from cell lysates was done using the Nucleospin™ RNA plus kit (Macherey-Nagel™) and reverse transcribed using SuperScript™ III First-Strand Synthesis System (Invitrogen) according to the manufacturer’s instructions. The resulting cDNA was used for quantitative real-time PCR (qRT-PCR) using specific primers and FastStart Essential DNA Green Master on a LightCycler 96 system (Roche). The resulting gene expression was normalized to *RPS9* and values were calculated using the comparative CT method. Primer sequences are as follows:

*Spic* forward: 5’-CCA CTT GGT TTT CCT GAA CGT-3’

*Spic* reverse: 5’-TTG CGG AAA TGT CAG CGA GTA-3’

*Hmox1* forward: 5’-GCC GAG AAT GCT GAG TTC ATG-3’

*Hmox1* reverse: 5’-TGG TAC AAG GAA GCC ATC ACC-3’

*Nr1h3* forward: 5’-TGT GCG CTC AGC TCT TGT-3’

*Nr1h3* reverse: 5’-TGG AGC CCT GGA CAT TAC C-3’

*Slc40a1* forward: 5’-CTA CCA TTA GAA GGA TTG ACC AGC T-3’

*Slc40a1* reverse: 5’-CAA ATG TCA TAA TCT GGC CGA-3’

*Pparg* forward: 5’-GAA AGA CAA CGG ACA AAT CAC C-3’

*Pparg* reverse: 5’-GGG GGT GAT ATG TTT GAA CTT G-3’

*Cd163* forward: 5′-CAT GTG GGT AGA TCG TGT GC-3’

*Cd163* reverse: 5′-TGT ATG CCC TTC CTG GAG TC-3’

*RPS9* forward: 5’-GGA TTT CTT GGA GAG GCG GC-3’

*RPS9* reverse: 5’-ACC TGC TTG CGG ACC CTA AT-3’

*Notch2* forward: 5’-TGG AGG TCT CAG TGG CTA TAA-3’

*Notch2* reverse: 5’-ATT CTG GCA TGG GTT AGA AAG A-3’

### Phenylhydrazine treatment

Phenylhydrazine stock (PHZ, Sigma-Aldrich) was diluted in PBS and administered in mice (2 mg/mouse) i.p. Mice were sacrificed at different time points and organs were collected for analysis. For blood parameter analysis, blood was drawn from the cheek and measured on a hematology analyzer (Scil Vet ABC, Antech Company, Viernheim, Germany).

### Anti-Notch2 antibody treatment

Anti-Notch2 and control antibodies have been previously described^86,87^ and were kindly provided by Genentech (San Francisco, CA, USA). Anti-Notch2 and control antibodies were injected at 20 mg/kg in PBS per adult mouse at two timepoints (days 0 and 7). Mice were euthanized on day 14.

### Inducible reporter studies

For inducible fate-mapping models, mice were subcutaneously injected at postnatal day 5 and 7 with 20 µl tamoxifen (10 mg/ml) (Sigma-Aldrich).

### Statistical analyses

All statistical analyses were performed using Graphpad Prism. Results are shown as mean ± standard error of mean (SEM). N numbers represent biological replicates and all experiments were performed at least two times unless otherwise stated. Groups were compared using unpaired two-tailed Student’s *t*-test with confidence interval of 95%. For comparison of multiple experimental groups, one-way or two-way analysis of variance (ANOVA) with Bonferroni’s multiple comparison test was used.

### Reporting summary

Further information on research design is available in the Nature Portfolio Reporting Summary linked to this article.

## Supplementary information

Supplementary Information

Description of Additional Supplementary Files

Supplementary Data 1

Supplementary Data 2

Supplementary Data 3

Supplementary Data 4

Supplementary Data 5

Supplementary Data 6

Supplementary Data 7

Supplementary Video 1

Supplementary Video 2

Reporting Summary

Transparent Peer Review file

## Source data

Source Data

## Data Availability

All relevant data are available in the Source Data provided with this paper. The scRNA sequencing data has been deposited to NCBI Gene Expression Omnibus under the accession code GEO GSE288621. Source data are provided with this paper.
