# Supplementary material for: Canonical Notch2 signaling regulates development and maintenance of iron-recycling macrophages and iron homeostasis
Source: Nat Commun. 2026 Sep 23;17:10132. doi: 10.1038/s41467-026-77938-7 (PMC13601512; doi:10.1038/s41467-026-77938-7)
Supplement: Supplementary file 1 [file 41467_2026_77938_MOESM12_ESM.pdf]

nature portfolio

Corresponding author(s): Prof. Florian P. Limbourg

Last updated by author(s): 18.08.2026

Reporting Summary

Nature Portfolio wishes to improve the reproducibility of the work that we publish. This form provides structure for consistency and transparency in reporting. For further information on Nature Portfolio policies, see our Editorial Policies and the Editorial Policy Checklist.

Statistics

For all statistical analyses, confirm that the following items are present in the figure legend, table legend, main text, or Methods section.

n/a	Confirmed	
☐	☒	The exact sample size (*n*) for each experimental group/condition, given as a discrete number and unit of measurement
☐	☒	A statement on whether measurements were taken from distinct samples or whether the same sample was measured repeatedly
☐	☒	The statistical test(s) used AND whether they are one- or two-sided<br>*Only common tests should be described solely by name; describe more complex techniques in the Methods section.*
☒	☐	A description of all covariates tested
☐	☒	A description of any assumptions or corrections, such as tests of normality and adjustment for multiple comparisons
☐	☒	A full description of the statistical parameters including central tendency (e.g. means) or other basic estimates (e.g. regression coefficient) AND variation (e.g. standard deviation) or associated estimates of uncertainty (e.g. confidence intervals)
☐	☒	For null hypothesis testing, the test statistic (e.g. *F*, *t*, *r*) with confidence intervals, effect sizes, degrees of freedom and *P* value noted<br>*Give P values as exact values whenever suitable.*
☒	☐	For Bayesian analysis, information on the choice of priors and Markov chain Monte Carlo settings
☒	☐	For hierarchical and complex designs, identification of the appropriate level for tests and full reporting of outcomes
☒	☐	Estimates of effect sizes (e.g. Cohen's *d*, Pearson's *r*), indicating how they were calculated

Our web collection on statistics for biologists contains articles on many of the points above.

Software and code

Policy information about availability of computer code

Data collection	Machines used for data collection are detailed in Methods Section of Paper.
Data analysis	Softwares used are detailed in Methods Section of Paper and additionally listed in Supplementary Table 2.

For manuscripts utilizing custom algorithms or software that are central to the research but not yet described in published literature, software must be made available to editors and reviewers. We strongly encourage code deposition in a community repository (e.g. GitHub). See the Nature Portfolio guidelines for submitting code & software for further information.

Data

Policy information about availability of data

All manuscripts must include a data availability statement. This statement should provide the following information, where applicable:

- Accession codes, unique identifiers, or web links for publicly available datasets
- A description of any restrictions on data availability
- For clinical datasets or third party data, please ensure that the statement adheres to our policy

A source data is provided with the paper. The authors declare that all relevant data are available in the source data. Data from sc-RNA Sequencing are deposited to NCBI Gene Expression Omnibus under the accession number GEO GSE288621 [https://www.ncbi.nlm.nih.gov/geo/query/acc.cgi?acc=GSE288621]

Research involving human participants, their data, or biological material

Policy information about studies with human participants or human data. See also policy information about sex, gender (identity/presentation), and sexual orientation and race, ethnicity and racism.

Reporting on sex and gender	*Use the terms sex (biological attribute) and gender (shaped by social and cultural circumstances) carefully in order to avoid confusing both terms. Indicate if findings apply to only one sex or gender; describe whether sex and gender were considered in study design; whether sex and/or gender was determined based on self-reporting or assigned and methods used.*<br>*Provide in the source data disaggregated sex and gender data, where this information has been collected, and if consent has been obtained for sharing of individual-level data; provide overall numbers in this Reporting Summary. Please state if this information has not been collected.*<br>*Report sex- and gender-based analyses where performed, justify reasons for lack of sex- and gender-based analysis.*
Reporting on race, ethnicity, or other socially relevant groupings	*Please specify the socially constructed or socially relevant categorization variable(s) used in your manuscript and explain why they were used. Please note that such variables should not be used as proxies for other socially constructed/relevant variables (for example, race or ethnicity should not be used as a proxy for socioeconomic status).*<br>*Provide clear definitions of the relevant terms used, how they were provided (by the participants/respondents, the researchers, or third parties), and the method(s) used to classify people into the different categories (e.g. self-report, census or administrative data, social media data, etc.)*<br>*Please provide details about how you controlled for confounding variables in your analyses.*
Population characteristics	*Describe the covariate-relevant population characteristics of the human research participants (e.g. age, genotypic information, past and current diagnosis and treatment categories). If you filled out the behavioural & social sciences study design questions and have nothing to add here, write "See above."*
Recruitment	*Describe how participants were recruited. Outline any potential self-selection bias or other biases that may be present and how these are likely to impact results.*
Ethics oversight	*Identify the organization(s) that approved the study protocol.*

Note that full information on the approval of the study protocol must also be provided in the manuscript.

Field-specific reporting

Please select the one below that is the best fit for your research. If you are not sure, read the appropriate sections before making your selection.

☒ Life sciences ☐ Behavioural & social sciences ☐ Ecological, evolutionary & environmental sciences

For a reference copy of the document with all sections, see nature.com/documents/nr-reporting-summary-flat.pdf

Life sciences study design

All studies must disclose on these points even when the disclosure is negative.

Sample size	Biometrical planning involved power analysis to determine Sample size. We used G*Power software (Faul, F., Erdfelder, E., Buchner, A., & Lang, A.-G. (2009). Statistical power analyses using G*Power 3.1: Tests for correlation and regression analyses. (Behavior Research Methods, 41, 1149-1160)
Data exclusions	Data from animals with unclear genotype or recombination were excluded
Replication	All experiments were performed twice or more to ensure data reproducibility. The number of experimental repeats are detailed in the figure legends.
Randomization	Mice were allocated to experimental groups on the basis of genotype: Ctrl mice are Cre negative, Experimental Mice are Cre positive.
Blinding	During data collection and processing, investigators were not aware of mice genotype.

Reporting for specific materials, systems and methods

We require information from authors about some types of materials, experimental systems and methods used in many studies. Here, indicate whether each material, system or method listed is relevant to your study. If you are not sure if a list item applies to your research, read the appropriate section before selecting a response.

Materials & experimental systems

n/a	Involved in the study
☐	☒ Antibodies
☒	☐ Eukaryotic cell lines
☒	☐ Palaeontology and archaeology
☐	☒ Animals and other organisms
☒	☐ Clinical data
☒	☐ Dual use research of concern
☒	☐ Plants

Methods

n/a	Involved in the study
☒	☐ ChIP-seq
☐	☒ Flow cytometry
☒	☐ MRI-based neuroimaging

Antibodies

Antibodies used	All antibodies used are listed and described in Supplementary Table 2.
Validation	All antibodies, with the exception of Ctrl and AntiNotch2 antibodies, are commercially available and validated. Ctrl and AntiNotch2 antibodies were kindly provided by Genentech and has been previously described and validated. Antibody titration for dilution was performed to determine specificity of staining before using experiment use. For immunohistochemistry experiments, we stained control slides of mouse organs that we know express the antigen (Immunochemistry staining and antibody titration on control slides). Positive controls were included during image acquisition.

Animals and other research organisms

Policy information about studies involving animals; ARRIVE guidelines recommended for reporting animal research, and Sex and Gender in Research

Laboratory animals	This study only involves the use of mice models. All mice strains used in this study are detailed in the Methods section of the manuscript under "Mice".
Wild animals	The study did not involve wild animals.
Reporting on sex	Findings apply to both sexes and therefore no sex-based analysis was performed. Experimental data were obtained from sex-matched animals with prespecified age. The majority of the experiments were performed with female mice with the exception of repopulation studies and experiments involving P0 and P1 mice, where both male and female recipients / mice were analyzed. Both sexes were used for these particular experiments as sex determination is challenging at early ages. Similarly, donor cells from fetal livers for repopulation studies were collected and pooled independent of sex. The majority of experiments were done with female mice to reduce aggression-related stress and variability, commonly observed in male mice.
Field-collected samples	The study did not involve field-collected samples
Ethics oversight	All animal studies were undertaken in accordance with German Animal Welfare legislation and with the European Communities Council Directive 2010/63/EU for the protection of animals used for experimental purposes. All experiments were approved by the local animal welfare board Niedersächsisches Landesamt für Verbraucherschutz und Lebensmittelsicherheit (LAVES) in Lower Saxony, Germany, or by Landesamt für Natur, Umwelt und Verbraucherschutz (LANUV) in North Rhine-Westphalia, Germany. This is also indicated in the Methods section of the manuscript.

Note that full information on the approval of the study protocol must also be provided in the manuscript.

Plants

Seed stocks	*Report on the source of all seed stocks or other plant material used. If applicable, state the seed stock centre and catalogue number. If plant specimens were collected from the field, describe the collection location, date and sampling procedures.*
Novel plant genotypes	*Describe the methods by which all novel plant genotypes were produced. This includes those generated by transgenic approaches, gene editing, chemical/radiation-based mutagenesis and hybridization. For transgenic lines, describe the transformation method, the number of independent lines analyzed and the generation upon which experiments were performed. For gene-edited lines, describe the editor used, the endogenous sequence targeted for editing, the targeting guide RNA sequence (if applicable) and how the editor was applied.*
Authentication	*Describe any authentication procedures for each seed stock used or novel genotype generated. Describe any experiments used to assess the effect of a mutation and, where applicable, how potential secondary effects (e.g. second site T-DNA insertions, mosiacism, off-target gene editing) were examined.*

Flow Cytometry

Plots

Confirm that:

☒ The axis labels state the marker and fluorochrome used (e.g. CD4-FITC).

☒ The axis scales are clearly visible. Include numbers along axes only for bottom left plot of group (a 'group' is an analysis of identical markers).

☒ All plots are contour plots with outliers or pseudocolor plots.

☒ A numerical value for number of cells or percentage (with statistics) is provided.

Methodology

Sample preparation	Sample preparation throughly described in Methods Section of this paper under "Tissue and Cell Preparation"
Instrument	LSR II, BD Biosciences
Software	FlowJo v. 10.8.0 LLC
Cell population abundance	For bone marrow monocytes, cell number was around 1-1.2x10^6 per mouse. For Spl, monocytes and RPM number was 1x10^4 - 5x10^5 per sample. Purity was more than 95%, determined by flow cytometry.
Gating strategy	Full gating strategies are provided in Supplementary Figures

☒ Tick this box to confirm that a figure exemplifying the gating strategy is provided in the Supplementary Information.
