# Supplementary material for: Canonical Notch2 signaling regulates development and maintenance of iron-recycling macrophages and iron homeostasis
Source: Nat Commun. 2026 Sep 23;17:10132. doi: 10.1038/s41467-026-77938-7 (PMC13601512; doi:10.1038/s41467-026-77938-7)
Supplement: Supplementary file 2 [file 41467_2026_77938_MOESM13_ESM.pdf]

nature portfolio

Peer Review File

Canonical Notch2 Signaling Regulates Development and Maintenance of Iron-recycling Macrophages and Iron Homeostasis

Corresponding Author: Professor Florian Limbourg

Parts of this Peer Review File have been redacted as indicated to maintain the confidentiality of unpublished data.

This file contains all reviewer reports in order by version, followed by all author rebuttals in order by version.

Version 0:

Reviewer comments:

Reviewer #1

(Remarks to the Author)
This study reveals that canonical Notch2 signaling is essential for the development and function of iron-recycling macrophages, specifically red pulp macrophages (RPM) in the spleen and bone marrow macrophages (BMM). Using conditional knockout mice (N2ΔCx3cr1 and RbpjΔCx3cr1), the authors demonstrate that loss of Notch2 or its mediator Rbpj disrupts RPM and BMM development, leading to iron overload in the spleen and bone marrow, as well as structural abnormalities in the spleen, including disrupted marginal zones and expanded CD169+ macrophages.
Minor points:
1.In Figure 5c, although all groups are compared to the first group, one-way ANOVA should still be used.
2.In Figure 4, since impaired splenic microarchitecture and iron homeostasis are critical phenotypes in Notch-deficient mice, the exact number of mice should be clearly indicated, rather than using a range (n=3-10) in the figure legend.
3.In Figure 6, the authors test whether Notch2 regulates RPM signature genes in response to heme, the results suggest that in the absence of DLL1, the impact of Notch deficiency on gene expression, even with hemin stimulation, is limited except for hmox1 and spic. Moreover, after culturing on immobilized DLL1, the regulation of gene expression by Notch deficiency becomes significant. Please explain the reasons for such results.

Reviewer #2

(Remarks to the Author)
The authors demonstrate that knockout of notch2, or its downstream effector Rbpj, in Cx3cr1-cre expressing cells causes defects in the number and organization of macrophage populations in the spleen, and the bone marrow though this is not as well characterized. These defects in the splenic macrophages could be rescued (partly) by transplant of WT but not Notch2 KO bone marrow or FL cells suggesting a requirement for notch 2 in the development of splenic red pulp macrophage (and bone marrow) both during development from embryonic macrophage progenitors and from fetal and adult definitive monocytes. Notch2 would represent a novel requirement for development and the phenotype is profound, but a lack of validation and necessary controls as well as dependence on a single cre-driver limit the scope and impact of the paper as described below.
1) Insufficient explanation is given for the use of CX3Cr1-cre; a brief explanation of the expression profile in development and across tissues would provide important context to interpret the results.

2) The authors state that Cre-negative littermates served as controls, but Cre+ mice are typically the preferred control for Cre-lox experiments due to the potential for off target effects of the cre recombinase activity, which would not be reflected in the cre- mice. I do appreciate the inclusion of the Cx3cr1-gfp control for the possibility of haploinsufficiency of the cx3cr1 gene; but cre+ Notch2 WT mice should also be analyzed to determine if the cre activity itself has any effect in the absence of the floxed gene.

3) While the phenotype is quite convincing, there is no data confirming the loss of Notch2 either at the protein or transcript level, or it is not prominently stated. This is necessary to support the author’s conclusion that “Notch2 regulates development

of RPM"

4) The quantification and presentation of the cell counts and flow data require some clarifications:
a. All bar graphs should show individual data points.
b. I am not familiar with the normalization of bone marrow cellularity by weight; this is typically done per 1e6 cells, per femur, per hind limb (all 3 bones), etc, but never by weight.
c. The relative and absolute quantifications are a bit confusing: in most cases the relative frequences do not match the range of values in the representative flow plots, so must be extrapolated to a % of total cells (or sometimes "monocytes" but this too is not defined"), and without information on the relative sizes of the parent populations, it is difficult to interpret the quantification of the flow data (see specific comments on Fig 2 below)
d. Confirmation of sorted populations such as the RPM and Ly6C monocytes by cytospin or flow analysis, or specific reference(s) in which these sorting strategies have already been validated, should be provided for the gating strategies for MMM, MZM, and WPM especially; alternatively, cytospins or confirmatory flow or IF of the sorted cell populations to confirm. In the bone marrow especially it has been shown that macrophage fragmentation during bone marrow collection results in F480+ membrane contamination on other cell populations, so extra care must be taken to ensure the population consists of the expected cell types.
e. Appropriate FMOs must be shown for the flow cytometry gating schemes, especially for the macrophage markers with unclear separation between + and – such as F480, Cd169, and TIM4. And an isotype control peak and/or unstained (on the same population) should be shown for the CD163 expression in fig 2. Macrophages often present with higher autofluorescence than other cells so evaluation of unstained for the mac subpopulation is essential to determine positivity.
f. Some experiments have as few as n=3 from just 2 independent experiments which seems a little low; at least 3 technical replicates would also be ideal, but this is difficult to gauge without the individual data points shown (as mentioned in a above)

5) In figure 2:
a. why is the gating diff for 1week compared to 4 and 10 weeks?
b. underneath flow plots it says "Gate: LiveCd45+lin lo/- CD117negCD11b+CD11Cneg", but clearly in the dot plots shown, there is Ly6C population, so parent pop is not Ly6C negative, so what is the parent gate here?
c. the quantification in fig 2 d-f says "relative frequency" of monocyte populations, but relative to what? I interpret relative frequency to refer to the proportion of the different populations gated out of the same parent population, in which case these should match the range of values in 2a-c (and this would give some indication of the consistency and reproducibility of the flow gating scheme), but these % are all much lower.
d. And for g-l, the % CD163+ cells, percents are very low for each population, again, % of what? And must have isotype or unstained control for cd163

6) The evaluation and description of erythropoiesis is inadequate. Proerythroblasts and the subsequent progressively maturing erythroblast stages (EryA and B, as used by the authors) are no longer capable of self-renewal and as such are erythroid PRECURSORS, not progenitors, as stated by the authors. And the pop they have labeled as EryC is just RBCs… and in the peripheral blood, pretty sure Ery B as gated would be mostly retics. RBC quantification should be by CBC from the whole blood and not evaluated in tissues since the appearance of RBCs is stochastic and a result of peripheral blood that is present in the vessels (or throughout the tissue as in the case of the spleen) but this does not necessarily reflect RBCs specifically being produced in the bone marrow or spleen and as such is irrelevant to quantify. CD71 loss during erythroid maturation is not very dramatic, particularly in stress erythropoiesis and is nut sufficient to separate early and late stages; if CD71/Ter119 is used, cells should be gated by a nuclear stain to exclude RBCs and best separation is CD71+/Ter119lo proE, then for the CD71+/Ter119+ cells you can separate on the basis of forward scatter FSChi (midEB) and FSClow (late EB) but all should have CD71 and nuclear stain to be erythroblasts. See Chen et al, PNAS 2009; https://www.pnas.org/doi/10.1073/pnas.0909296106

7) Extramedullary erythropoiesis can also affect the splenic architecture, expanding the red pulp and making the marginal zone and white pulp borders less clear so it would be helpful to compare the ko to a WT mouse stressed by phlebotomy to control for any the changes induced by the EMH alone.

[redacted]

Other general comments
1) If total spleen cellularity was not different, I would expect that the %CD45+ cells in the spleen goes down in the KO because of the increased abundance of CD45- erythroblasts, is this the case? and was total bone marrow cellularity different at all? (not by weight, but by bone or other method?)
2) I know (or at least I think) that the mG in the mTmG model is membranous GFP, but this should be stated at least once before the mG abbreviation is used.
3) Figures are too crowded and difficult to follow. There are few, if any cases where the % of cells shows anything different from the absolute counts so I don't see any reason to include the graphs of % of cells at all when the absolute number (per

spleen) is more relevant. Order of panels should be reviewed and made more logical.
4) In figure 1 b, c, h; spleen weight and cellularity, the P0 and p1 are too small to read, what is even the point of including these? Utilize broken axis (better than in c) or separate to separate fig, or even put in supplemental if there really is no difference
5) I am slightly confused by the qPCR data showing that spic expression is down in ly6Chi mono, but not CD163 which is a spic-induced gene. Similarly, what do you think is driving CD163 increase in the Ly6clo population where spic is unchanged, or maybe even down a little? The authors could discuss this more and speculate on on what this implies about the relationship between spic expression and CD163.
6) The IF images and videos are very nice, and qualitatively seem to confirm the quantitation from the flow, but when the macrophages are disorganized and spread out more, it is difficult to judge the abundance. I wonder if a volumetric analysis of the images could be done and provide an in situ confirmation of the trends observed in the flow (with respect to the macrophage populations) to exclude any potential artifacts or cell drop out due to preparation of the cells for flow.

Reviewer #3

(Remarks to the Author)
Schroth et al. studied the role of Notch2 in the development and function of iron-recycling macrophages. The researchers showed that Notch2 depletion in Cx3cr1-positive macrophages led to a significant decrease in red pulp macrophages (RPM) and moderately decreased levels of bone marrow macrophages (BMM), which was followed by a late compensatory response expansion of CD163+ monocytes. However, these cells were not studied further. The lack of macrophages resulted in previously known iron accumulation in the spleen (e.g. PMID: 19037245, PMID: 38272969). The researchers also performed elegant adoptive transfer studies with total fetal liver and adult bone marrow cells. They showed the reconstitution of macrophages and the structure of the spleen but did not indicate, more specifically, the Notch2-dependent progenitor(s) that would be responsible for this reconstitution.

Major:
1. A bar graph of the mean of data should be presented with overlapping dots for better individual observation of that variable and the real number of animals used in each experiment.

2. The author should clearly indicate which experiments were repeated only twice. To ensure more reliable data, a minimum of three independent experiments should be conducted in the majority of experiments. It is also unclear if both male and female mice were used as a mix in the same experiments or if some experiments were only performed with one sex.

3. The FASC gating should be presented more clearly. For example, the gating for the Ly6Clo population, sorted in Figure 1, is not displayed in any of the gating strategies. In Figures 2a-c, splenic monocytes are gated from CD11b+ CD11c- cells, but this gating is not shown. Overall, the gating strategy is too complex, and it is difficult to understand which gating was applied to each experiment.

4. In Figure 1, algorithms like unsupervised k-means should be applied to the original high-dimensional data. t-SNE is primarily a visualisation tool and not ideal for clustering or quantitative analysis since it does not preserve real distances or densities. The analysis should also be done from single samples to see the variation in decreased cell numbers in different cell populations between the genotypes.

5. ``The relative frequency or absolute numbers of each cell subset were calculated from the live cell gate and normalised per Spl``. Even the spleens were different sizes? How was this considered in calculations?

6. Figure 1, ``N2ΔCx3cr1 mice exhibited splenomegaly starting at 4 weeks (wk) of age but showed no changes in Spl total cell counts`` Why is spleen size growing but not total cell numbers? How was the data normalised?

[redacted]

Using models such as CX3CR1creERT2, Ms4a3cre/ERT2 mice, or RankCre, authors could point to the time but also the progenitor of Notch2-dependent RPM and BMM cells and provide evidence supporting the statement, “Several lines of evidence suggest a unique, but possible shared origin and developmental pathway of RPM and BMM”

8. Using a single-cell sequencing experiment to validate the finding in flow cytometry (Figure 3) is a bit confusing. Was there a difference in the phenotype between monocytes or remaining RPM in Ctrl, N2ΔCx3cr1 and RbpjΔCx3cr1? Or are the expanding monocytes seen in N2ΔCx3cr1 mice similar in phenotype to those in the ctrl mice?

9. Was the function of the remaining RPMs, such as phagocytosis, preserved in the N2ΔCx3cr1 model?

Minor:
1. Ly6Clo (#5) monocytes (line 118) or CD43+Ly6Clo monocytes (#5)(line 120) ?

2. In figures, some more arrows would help the reader, such as in Figure 4, showing the disruption of MZ ring structure.
3. The results section sometimes overstretches into a discussion and can complicate the observations.

Version 2:

Reviewer comments:

Reviewer #1

(Remarks to the Author)
General Comments
The revised manuscript by Schroth et al. has been significantly improved. The authors have rigorously addressed the concerns raised during the first round of review, particularly regarding the potential for Cre-mediated off-target effects. By incorporating comprehensive genetic controls (Cx3cr1+/+ Notch2f/f, Cx3cr1+/+ Notch2+/+, Cx3cr1Cre/+ Notch2+/+) and utilizing an anti-Notch2 antibody in wild-type mice, the study now convincingly demonstrates that the loss of canonical Notch2-Rbpj signaling directly disrupts red pulp macrophages (RPM) [redacted]

The manuscript provides valuable mechanistic insights into macrophage ontogeny and iron homeostasis. The elegant adoptive transfer models (CD45.1+ bone marrow, fetal liver cells, and mTmG) confirm that the reconstitution of the defective splenic niche is strictly dependent on Notch2-competent progenitors. Furthermore, the observation that Notch2 deficiency triggers a local compensatory expansion of CD163+ Ly6Clo/int monocytes and confers an initial adaptive advantage during PHZ-induced hemolytic anemia highlights a highly dynamic cross-talk between macrophage niches and extramedullary hematopoiesis. The findings are novel and carry substantial translational potential for manipulating iron-recycling macrophages in vascular and hemolytic diseases.

While the primary conclusions are now exceptionally well-supported, there are two remaining points that require the authors' attention prior to final acceptance.

Specific Comments

Inclusion of Functional Assays: In the rebuttal, the authors provided a CFSE-labeled erythrocyte phagocytosis assay demonstrating reduced phagocytic activity in residual Notch2-deficient RPMs and a compensatory increase in monocytes. This is a crucial piece of functional evidence. If this assay is solely confined to the response letter, the authors must either incorporate it into the main manuscript (or supplementary materials) or temper their claims regarding Notch2's regulation of macrophage "function" in the main text.

scRNA-seq Statistical Rigor: The single-cell transcriptomic data provides robust molecular confirmation of the flow cytometry results. However, given that the design multiplexes two mice per genotype onto a single 10x lane, the analytical approach needs further clarification. The authors must detail their QC thresholds, HTO demultiplexing criteria, and doublet handling strategies. More importantly, differential expression analysis (DEG) and pathway enrichment must be performed using a replicate-aware or pseudobulk framework to avoid statistical inflation. If conventional cell-level statistics were used, the transcriptomic conclusions should be explicitly framed as exploratory and supportive rather than definitive.

Recommendation:
Accept with Minor Revisions. (The authors have thoroughly addressed the majority of previous critiques, resulting in a robust manuscript; addressing the two minor methodological points above will finalize the work for publication).

Reviewer #2

(Remarks to the Author)
I commend the authors on the additional data provided which has at least attempted to address the reviewer concerns. The presentation of the data is improved with respect to transparency of individual values and number of replicates and important controls have been added. However, in some cases I feel the authors may have misunderstood the intention of the reviewer comments, and in many cases, the additions of figures or text do not improve the clarity of the data or the manuscript. I will concede that this may merely be a stylistic preference and defer to the authors if they are happy with the manuscript in its current form. I have listed a few minor comments for their consideration below:

Major
1) The authors added some lovely validation data showing that antibody-mediated blocking of notch2 can phenocopy the loss of RPM observed in Notch2-deleted animals however, this is a little confusing and requires better explanation in the text.
a. In the text they state "treatment of adult wild-type (wt) mice with anti-Notch2 (aN2) antibody, but not control antibody (Ctrl-Ab), was sufficient to phenocopy…" (lines 165-166), but this would demonstrate that notch2 is also required for maintenance of the RPM pool in adults, rather than a role in development. But,
b. In the methods (supplemental, line 1003), it states that anti-Notch2 and control antibodies were injected at 20mg/kg per mouse in PBS on days 0 and 7" which is much more appropriate for demonstrating a role in development.
This obviously requires clarification.

2) I appreciate the authors including the requested FMOs, which are meant to serve as guides for the setting gates on true positive events. What is the interpretation of the high % positive in the FMOS for CD169 and TIM4 (6.5% and 3.4% respectively, supp fig 3b). If 6.5% positive is the background for CD169, what then does 9% positive in the WT really mean?

3) line 448 references supp fig 14, but I believe this should be 15

Minor points

4) As the characterization of the phenotype relies heavily on abundance of particular cell populations, I find the continued reliance on relative frequence, or % of parent or live cell population, difficult to interpret. I concede that in some cases, this can accurately reflect the changes that are occurring, but also note that care must be taken in the interpretation of these because they are indeed "relative" and can be misleading depending on the circumstances, especially when there are changes in overall cellularity or abundance of the parent population. Absolute numbers would always be preferred and unless it serves some purpose, I see no reason to include both as in Fig 1.

5) In the tsne clustering in Fig 1, it is not clear why only clusters 1-7 are highlighted and quantified, especially when #5,6,7, and 8 were decreased in the N2 KO (line 149-150), so 8 at least should also be labeled and quantified in the main figure. Why only the first 7?

6) The authors state that residual RPM in αN2-treated wt mice displayed increased CD163 surface expression, as reflected by the increased CD163 MFI. (supp fig 5d-h) (line 213). In Supp Fig 5h, the rpm population is bimodal for CD163 and there appears to be an increase in the number with the higher expression. Could the authors clarify if the actual mfi of that population is increased relative to the same pop in WT or is the overall mfi is just higher because there more are in the CD163 high pop relative to the lo pop?

7) CD163 is not mentioned in the section on the transcriptomics differences in RPM and Ly6Clo monos, despite the previous paragraph being about increased CD163 expression...it is not shown in the bubble plot (3c, or any of the volcano plots 3e-g) nor is it identified as differentially expressed in most of the comparisons. Protein does not always = transcript and there may be many explanations for this, but the authors should at least mention this disparity rather than avoid it.

8) Much of the added transcriptomic analysis does not contribute much to the overall conclusions of the paper. The authors could really just hit the highlights in a quick summary and move the rest of the details to the legend of the supp figure.

9) The transplant experiments are nice, but not well explained. If "neither BM nor Spl cell transfer rescued BMM" (line 348) then why did "Transfer of the ctrl mG BM cells did rescue the BMM" (line 385). I searched for details if these were GFP+ sorted or something to explain the difference, but appeared to be total bone marrow? Why the difference in rescue?

10) If I understand the flow gating figures correctly, the same gating strategy was used for BMM as for RPM, meaning all CD11b-/CD11c- cells were excluded. The authors should take note that there is a macrophage population in the murine bone marrow which is Cd11b- that is involved in the erythropoietic niches which is not quantified by their analysis. [redacted] To be clear, I am not asking the authors to perform additional analyses, but rather to acknowledge the limitations of their bone marrow macrophage definition and the possibility that other niche macrophages may or may not play a role in their phenotype.

Reviewer #3

(Remarks to the Author)
I have reviewed the revised manuscript by Schroth et al. The authors have adequately addressed most of my comments through revisions to the text and the inclusion of additional experimental evidence. The revised article is clearly improved. I have no further comments.

Version 3:

Reviewer comments:

Reviewer #1

(Remarks to the Author)
I consider this to be a well-developed study with a clear rationale and a relatively complete body of data, and I recommend publication after minor revision. The manuscript focuses on the role of canonical Notch2-RBPJ signaling in the development, maintenance, and iron homeostatic function of iron-recycling macrophages, including RPM and BMM. By combining genetic models, antibody-mediated blockade, single-cell transcriptomics, tissue imaging, iron quantification, adoptive transfer experiments, and in vitro induction assays, the authors establish a coherent line of evidence.

The authors show that conditional deletion of Notch2 or its downstream mediator Rbpj leads to a marked reduction of splenic RPM and bone marrow BMM, accompanied by iron accumulation in the spleen and bone marrow, downregulation of RPM signature genes, expansion of CD163+ monocytes, and enhanced extramedullary hematopoiesis. Anti-Notch2 antibody treatment in adult wild-type mice recapitulates part of the phenotype, suggesting that Notch2 is involved not only in development but also in the maintenance of the mature iron-recycling macrophage pool. The in vitro DLL1-hemin experiment further links Notch2 signaling to the heme-induced RPM differentiation program. Compared with previous studies implicating Spic, Pparg, Hmox1, CSF-1, and IL-33 in RPM development and function, this study incorporates Notch2-RBPJ signaling into this regulatory network and provides a new mechanistic connection.

The following points could be improved:

1. Individual conclusions should be slightly tempered. For example, the statement “Notch2 deficiency confers improved adaptation to hemolytic anemia” could be revised to “is associated with an altered early response to PHZ-induced hemolytic anemia.” This result is based on early observations in a specific acute hemolysis model and should not be overinterpreted as indicating that Notch2 deficiency is generally beneficial.

2.The interpretation of the single-cell transcriptomic and pathway enrichment results should remain cautious in the Discussion. Some enriched pathways, such as metabolic, inflammatory, and mitochondrial pathways, are relatively broad. The authors should emphasize that these analyses mainly support and help interpret the observed phenotypes, rather than serving as standalone mechanistic conclusions.

3. The authors may consider adding or improving a summary model figure to help readers understand the relationships among Notch2-RBPJ, DLL1/heme, RPM/BMM, iron homeostasis, and extramedullary hematopoiesis.

Reviewer #2

(Remarks to the Author)
No further comments.

Open Access This Peer Review File is licensed under a Creative Commons Attribution 4.0 International License, which permits use, sharing, adaptation, distribution and reproduction in any medium or format, as long as you give appropriate credit to the original author(s) and the source, provide a link to the Creative Commons license, and indicate if changes were made.
In cases where reviewers are anonymous, credit should be given to 'Anonymous Referee' and the source.
The images or other third party material in this Peer Review File are included in the article’s Creative Commons license, unless indicated otherwise in a credit line to the material. If material is not included in the article’s Creative Commons license and your intended use is not permitted by statutory regulation or exceeds the permitted use, you will need to obtain permission directly from the copyright holder.
To view a copy of this license, visit https://creativecommons.org/licenses/by/4.0/

REVIEWER COMMENTS

Reviewer #1 (Remarks to the Author):

This study reveals that canonical Notch2 signaling is essential for the development and function of iron-recycling macrophages, specifically red pulp macrophages (RPM) in the spleen and bone marrow macrophages (BMM). Using conditional knockout mice (N2ΔCx3cr1 and RbpjΔCx3cr1), the authors demonstrate that loss of Notch2 or its mediator Rbpj disrupts RPM and BMM development, leading to iron overload in the spleen and bone marrow, as well as structural abnormalities in the spleen, including disrupted marginal zones and expanded CD169+ macrophages.

We would like to thank the Reviewer #1 for the important comments which we addressed with new experiments. We provide our response here in the point-by-point fashion and believe our effort has significantly improved the revised manuscript.

Minor points:

1. In Figure 5c, although all groups are compared to the first group, one-way ANOVA should still be used.

For **Fig. 5c, e**, we had used one-way ANOVA but mislabeled the figure legend. This has now been corrected (**Fig. 5c, e** in the revised manuscript).

2. In Figure 4, since impaired splenic microarchitecture and iron homeostasis are critical phenotypes in Notch-deficient mice, the exact number of mice should be clearly indicated, rather than using a range (n=3-10) in the figure legend.

As suggested by the editor and all reviewers, we have indicated the exact number of mice in all figure legends and plotted single data points in all the bar graphs. For graphs where it is difficult to discern (i.e. overlapping points or n numbers more than 10), please refer to source data and/or figure legend.

3. In Figure 6, the authors test whether Notch2 regulates RPM signature genes in response to heme, the results suggest that in the absence of DLL1, the impact of Notch deficiency on gene expression, even with hemin stimulation, is limited except for hmox1 and spic. Moreover, after culturing on immobilized DLL1, the regulation of gene expression by Notch deficiency becomes significant. Please explain the reasons for such results.

Activation of Notch signaling is strictly ligand-dependent, e. g. DLL1. Therefore, there is no difference in gene expression under control conditions (Ctrl and *N2*$^{\Delta Cx3cr1}$) in the absence of DLL1 and Heme (Hemin). In addition, target genes may be regulated independently and synergistically by DLL1-Notch2 and Hemin. Specifically, Hemin might trigger *Spic* and *Hmox1* expression [1]. Similarly, DLL1 can upregulate *Spic*, *Slc40a1* and *Nr1h3*. However, *Spic, Slc40a1* and *Nr1h3* upregulation is most prominent with Hemin and DLL1 co-stimulation, which is Notch2-dependent. Of note, synergistic signaling in the context of Notch2 has been described in our previous study [2]. On the other hand, genes *Pparg* and *Hmox1* are primarily regulated by Heme in a Notch-independent fashion.

Reviewer #2 (Remarks to the Author):

The authors demonstrate that knockout of notch2, or its downstream effector Rbpj, in Cx3cr1-cre expressing cells causes defects in the number and organization of macrophage populations in the spleen, and the bone marrow though this is not as well characterized. These defects in the splenic macrophages could be rescued (partly) by transplant of WT but not Notch2 KO bone marrow or FL cells suggesting a requirement for notch 2 in the development of splenic red pulp macrophage (and bone marrow) both during development from embryonic macrophage progenitors and from fetal and adult definitive monocytes. Notch2 would represent a novel requirement for development and the phenotype is profound, but a lack of validation and necessary controls as well as dependence on a single cre-driver limit the scope and impact of the paper as described below.

We would like to thank the Reviewer #2 for the constructive comments. Based on these, we have performed new experiments and additional analyses to address the questions raised by the Reviewer. In particular, we validated our observations in a Cre-independent manner through antibody-mediated blocking of Notch2 receptor signaling (**Fig. 1i, j**, **Supplementary Fig. 5d-h**, **Supplementary Fig. 15j, k**). Our results confirm and strengthen our statement that, independent of the model used, whether conditional gene- or pharmacological targeting, Notch2 is indispensable for the development and maintenance of these macrophage populations. We provide our response here in a point-by-point fashion and believe our effort has significantly improved the revised manuscript.

1) Insufficient explanation is given for the use of CX3Cr1-cre; a brief explanation of the expression profile in development and across tissues would provide important context to interpret the results.

We have now included a brief explanation in the revised manuscript regarding this point (line 116 in revised manuscript). In brief, *Cx3cr1* is specifically expressed in the progenitors of mononuclear phagocytes, both in the embryonic and postnatal period. Taking advantage of *Cx3cr1*'s restricted expression in myeloid-derived cells, *Cx3cr1-Cre* allows for targeted and stable genetic manipulation in macrophage progenitors in a Cre/loxP system-dependent manner. We would also like to refer to Reference #38 in the initial submission (Yona et. al) [3] in the main manuscript for a more detailed description of *Cx3cr1-Cre*.

2) The authors state that Cre-negative littermates served as controls, but Cre+ mice are typically the preferred control for Cre-lox experiments due to the potential for off target effects of the cre recombinase activity, which would not be reflected in the cre-mice. I do appreciate the inclusion of the Cx3cr1-gfp control for the possibility of haploinsufficiency of the cx3cr1 gene; but cre+ Notch2 WT mice should also be analyzed to determine if the cre activity itself has any effect in the absence of the floxed gene

As suggested by the reviewer and also requested by the editor, we performed additional extensive studies and analyzed four different groups of mice: 1. *Cx3cr1*$^{+/+}$ *Notch2*$^{f/f}$ (Ctrl), 2. *Cx3cr1*$^{+/+}$ *Notch2*$^{+/+}$, 3. *Cx3cr1*$^{Cre/+}$ *Notch2*$^{+/+}$. 4. *Cx3cr1*$^{Cre/+}$ *Notch2*$^{f/f}$ (*N2*$^{\Delta Cx3cr1}$) in four different analyses including, **a**) quantification of relative and absolute numbers of RPM and BMM (**Supplementary Fig. 4a, b), b**) Prussian blue staining of spleens (**Supplementary Fig. 10a**) and (**c**) quantification of iron (**Supplementary Fig. 10b, c**), and **d**) expression of iron metabolism-associated genes in sorted RPM and Ly6C^{hi} and Ly6C^{lo} monocytes (**Supplementary Fig. 4c-e**). These experiments confirmed our statements and excluded any

potential off-target effects of genetic (both *Cre* and *flox*) manipulations. Thus, neither *Cre* activity nor *flox* itself have any influence in the described phenotypes of *N2*$^{\Delta Cx3cr1}$ mice in **Fig. 1**.

[redacted]

[redacted]

3) While the phenotype is quite convincing, there is no data confirming the loss of Notch2 either at the protein or transcript level, or it is not prominently stated. This is necessary to support the author's conclusion that "Notch2 regulates development of RPM"

In the revised manuscript, we provide gel-electrophoresis images showing the presence of the recombinant (*ΔNotch2*, 599bp) band in sorted Ly6C^{hi} and Ly6C^{lo} monocytes in Spl and BM, and in RPM and BMM from *N2*$^{\Delta Cx3cr1}$ but not from Ctrl mice (**Supplementary Fig. 1a, b**). We have also included flow cytometry and qRT-PCR data showing the expression/deletion of Notch2 in BM and splenic monocytes, RPM and BMM (**Supplementary Fig. 1c-i**). In addition, we have previously confirmed the deletion of Notch2 in the BM monocytes and their progenitors in a recent publication from our group [4] and is also depicted in **Supplementary Fig. 1b, f for BM monocytes.**

We have also included information regarding the deletion efficiency (calculated from the reduction in Notch2^{+} cells in *N2*$^{\Delta Cx3cr1}$ relative to Ctrl mice) after staining and flow-cytometry analysis in the revised manuscript text and in materials and methods.

In these data, we did not observe direct loss of Notch2 receptor in the few remaining RPM and BMM in qRT-PCR and/or flow cytometry, despite the presence of the recombinant *ΔNotch2*, 599bp band. Although Notch2 deletion was mostly targeted to BM monocytes and their progenitors, as seen in **Supplementary Fig. 1b, c, f** and our recent publication [4], respectively, our data support a requirement for Notch2 signaling in the development of RPM/BMM populations. In line with this, our repopulation experiments with Notch2-competent BM, FL and sorted BM Ly6C^{hi} monocytes show that only Notch2-competent cells were able to reconstitute the defective niches (**Fig. 5, 6, Supplementary Fig. 14**). While we cannot exclude that Notch2 is not efficiently targeted in RPM/BMM populations using *Cx3cr1-Cre*, the inability of Notch2-deficient precursors to generate functional RPM/BMM pools demonstrate a functional requirement for Notch2 signaling in their establishment and maintenance. In confirmation of this, we observed deletion of Notch2 in *N2*$^{\Delta Cx3cr1}$ Ly6C^{hi} monocytes – a potential macrophage precursor cell - both in Spl and BM (**Supplementary Fig. 1**).

4) The quantification and presentation of the cell counts and flow data require some clarifications:

a. All bar graphs should show individual data points.

We have corrected all bar graphs in revised manuscript and addressed this point above (see our reply to Reviewer #1, Point 2).

b. I am not familiar with the normalization of bone marrow cellularity by weight; this is typically done per 1e6 cells, per femur, per hind limb (all 3 bones), etc, but never by weight.

We normalize bone marrow cellularity by the weight of the bone marrow in order to minimize experimental variation, as techniques for bone marrow isolation may slightly differ between individuals i.e. handling and processing of bones, bone size, as well as age and sex of mice. Also, this method has been referenced in several published studies, see **Fig. 5c** in Koh et. al [5], **Fig. 2d** in Gamrekelashvili et. al [6] and **Supplementary Fig. 2j-l** in Xu et. al [4].

Nevertheless, we also normalized bone marrow cellularity per $1x10^6$ cells as suggested and show that both methods of normalization yield the same significant outcome (**Fig. 2 for reviewers,** related to **Fig. 1r** in revised manuscript).

c. The relative and absolute quantifications are a bit confusing: in most cases the relative frequences do not match the range of values in the representative flow plots, so must be extrapolated to a % of total cells (or sometimes "monocytes" but this too is not defined"), and without information on the relative sizes of the parent populations, it is difficult to interpret the quantification of the flow data (see specific comments on Fig 2 below)

The relative values ('Cells (%)' or relative frequency of cells) in bar graphs reflect frequency of respective population within 'live cell gate'. In the representative flow cytometry plots (e.g. **Fig. 1g, i, k, q**), values are depicted as frequency of parental gate and is determined by FlowJo program. We have now provided this information in the materials and methods under "Flow Cytometry and Cell sorting" as well as in the figure legends (for example in Fig. 1 figure legend) for clarity.

d. Confirmation of sorted populations such as the RPM and Ly6C monocytes by cytospin or flow analysis, or specific reference(s) in which these sorting strategies have already been validated, should be provided for the gating strategies for MMM, MZM, and WPM especially; alternatively, cytospins or confirmatory flow or IF of the sorted cell populations to confirm. In the bone marrow especially it has been shown that macrophage fragmentation during bone marrow collection results in F480+ membrane contamination on other cell populations, so extra care must be taken to ensure the population consists of the expected cell types.

Cytospins as well as confocal images are now included in **Supplementary Fig. 3** in the revised manuscript. For information, we would also like to reference the following articles, where the gating strategy/definition for RPM and BMM populations are similar, if not identical [1, 7, 8].

e. Appropriate FMOs must be shown for the flow cytometry gating schemes, especially for the macrophage markers with unclear separation between + and – such as F480, Cd169, and TIM4. And an isotype control peak and/or unstained (on the same population) should be shown for the CD163 expression in fig 2. Macrophages often

present with higher autofluorescence than other cells so evaluation of unstained for the mac subpopulation is essential to determine positivity.

We have included this information in revised manuscript (**Supplementary Fig. 2o, r-t, Supplementary Fig. 3a, b and Supplementary Fig. 5a-c**).

f. Some experiments have as few as n=3 from just 2 independent experiments which seems a little low; at least 3 technical replicates would also be ideal, but this is difficult to gauge without the individual data points shown (as mentioned in a above)

We have corrected all bar graphs in revised manuscript and addressed this point above (see our reply to Reviewer #1, Point 2).

5) In Figure. 2:

a. why is the gating diff for 1week compared to 4 and 10 weeks?

In the early postnatal period, the spleen is undergoing dramatic structural and functional changes characterized by significant cellular recruitment and proliferation (**Supplementary Fig. 11a-c**), accompanied by active splenic erythropoietic activity [9]. We optimized settings for these conditions, since immature cells often present different forward/side scatter properties and some markers have variable expression in neonates. The gating shown for 1wk old mice was set up using staining controls. Gating the populations in the way as depicted for older mice (4- and 10 wk) would exclude the majority of $CD43^{+}$ cells in 1 wk old mice.

b. underneath flow plots it says "Gate: LiveCd45+lin lo/- CD117negCD11b+CD11Cneg", but clearly in the dot plots shown, there is Ly6C population, so parent pop is not Ly6C negative, so what is the parent gate here?

The gates in the top and bottom row (2D plots, **Fig. 2a-c**) are gated from $LiveCD45^{+}Lin^{lo/-}CD117^{neg}CD11b^{+}CD11C^{neg}$ cells as stated (below the representative flow cytometry plots). For visualization, 2D flow cytometry plots using Ly6C vs CD43 (top panel) or Ly6C vs CD163 (bottom panel) are shown. These 4 populations (#1-#4) are derived from $LiveCD45^{+}Lin^{lo/-}CD117^{neg}CD11b^{+}CD11C^{neg}$ parental gate. See **Supplementary Fig. 2i** for parental gate, and **Supplementary Fig. 5a, b** for downstream gating of $CD163^{+}$ monocytes. See **Supplementary Fig. 2n** and **Supplementary Fig. 5c** for downstream gating of $CD163^{+}$ RPM).

c. the quantification in fig 2 d-f says "relative frequency" of monocyte populations, but relative to what? I interpret relative frequency to refer to the proportion of the different populations gated out of the same parent population, in which case these should match the range of values in 2a-c (and this would give some indication of the consistency and reproducibility of the flow gating scheme), but these % are all much lower.

We have addressed this point in the revised manuscript and provided explanation in our reply above (see our reply to Reviewer #2, Point 4c).

d. And for g-I, the % CD163+ cells, percents are very low for each population, again, % of what? And must have isotype or unstained control for cd163

We have addressed this point in the revised manuscript and provided explanation in our reply above (see our reply to Reviewer #2, Point 4c).

We have included unstained and FMO controls as requested, in **Supplementary Fig. 5b, c** in revised manuscript.

6) The evaluation and description of erythropoiesis is inadequate. Proerythroblasts and the subsequent progressively maturing erythroblast stages (EryA and B, as used by the authors) are no longer capable of self-renewal and as such are erythroid PRECURSORS, not progenitors, as stated by the authors. And the pop they have labeled as EryC is just RBCs…and in the peripheral blood, pretty sure Ery B as gated would be mostly retics. RBC quantification should be by CBC from the whole blood and not evaluated in tissues since the appearance of RBCs is stochastic and a result of peripheral blood that is present in the vessels (or throughout the tissue as in the case of the spleen) but this does not necessarily reflect RBCs specifically being produced in the bone marrow or spleen and as such is irrelevant to quantify. CD71 loss during erythroid maturation is not very dramatic, particularly in stress erythropoiesis and is nut sufficient to separate early and late stages; if CD71/Ter119 is used, cells should be gated by a nuclear stain to exclude RBCs and best separation is CD71+/Ter119lo proE, then for the CD71+/Ter119+ cells you can separate on the basis of forward scatter FSChi (midEB) and FSClow (late EB) but all should have CD71 and nuclear stain to be erythroblasts. See Chen et al, PNAS 2009; https://www.pnas.org/doi/10.1073/pnas.0909296106

As suggested, we have replaced the word "progenitors" with "precursors". In the blood, we have taken out the graphs depicting "EryC" and replaced "EryB" with the term "Retic (Reticulocytes)", as suggested (**Fig. 7b-e, Supplementary Fig. 15**). RBC quantification using CBC was performed as shown in **Supplementary Fig. 2a-h, Supplementary Fig. 17c-n, Fig. 8a-d**.

In the revised manuscript, we show a gating strategy that includes a nuclear stain (thiazole orange, TO), as suggested (**Supplementary Fig. 15c-i**). We confirm in this alternative gating strategy that >99% of the cells of interest (now defined as ProE, midEB, lateEB, as suggested) are TO^+ (**Supplementary Fig. 15c-i**, with TO), showing that our original gating strategy (**Supplementary Fig. 15a, b**, without TO) is valid. For the ProE definition, we have also revised our gating strategy and included CD117 as a positive marker. We have taken the reviewer's suggestion of population definition for ProE, midEB and lateEB and have performed new analysis in the revised manuscript (**Fig. 7b-e, Supplementary Fig. 15a, b, Supplementary Fig. 15e-i**).

7) Extramedullary erythropoiesis can also affect the splenic architecture, expanding the red pulp and making the marginal zone and white pulp borders less clear so it would be helpful to compare the ko to a WT mouse stressed by phlebotomy to control for any the changes induced by the EMH alone.

In response to this insightful comment, we performed phlebotomy and confirmed that extramedullary erythropoiesis is not responsible for the altered splenic architecture observed in Notch2 KO mice. WT mice stressed by phlebotomy exhibit the typical splenic architecture despite induction of extramedullary hematopoiesis (**Supplementary Fig. 16e, f**).

In addition, PHZ-treated wt (Ctrl) mice on day 17 show normal MZ architecture (clear CD169^{+} MMM ring and F4/80 staining in RP, **Fig. 8o**) despite extramedullary hematopoiesis as shown by CD71^{+} islets in RP (**Fig. 8n**) which is in agreement with phlebotomy studies.

[redacted]

[redacted]

[redacted]

[redacted]

Other general comments

1) If total spleen cellularity was not different, I would expect that the %CD45+ cells in the spleen goes down in the KO because of the increased abundance of CD45- erythroblasts, is this the case? and was total bone marrow cellularity different at all? (not by weight, but by bone or other method?)

Indeed, the frequency of CD45^{+} cells (from live cell gate) is reduced in KO animals (**Fig. 4a for reviewers**). There is no significant difference in total bone marrow cellularity by leg (femur and tibia) between Ctrl and KO mice (**Fig. 4b for reviewers**).

2) I know (or at least I think) that the mG in the mTmG model is membranous GFP, but this should be stated at least once before the mG abbreviation is used.

A short description of the model is now included in the revised manuscript (Page 10, Line 379).

3) Figures are too crowded and difficult to follow. There are few, if any cases where the % of cells shows anything different from the absolute counts so I don't see any reason to include the graphs of % of cells at all when the absolute number (per spleen) is more relevant. Order of panels should be reviewed and made more logical.

We have chosen to present our flow cytometry data (bar graphs) as percent of live cells because this normalization allows for a more accurate comparison across experimental replicates. Absolute cell numbers (per spleen) can vary due to minor differences in sample processing and technical variability.

While we agree that some figures are complex, we are limited by journal standard in the number of main figures. We believe that presenting the main figures as we have in the manuscript maintains the visual continuity and overall flow of the manuscript, in line with a larger number of publications in Nature communications. Alternative arrangements or moving some figures to supplementary information might disrupt the coherence of the manuscript.

4) In figure 1 b, c, h; spleen weight and cellularity, the P0 and p1 are too small to read, what is even the point of including these? Utilize broken axis (better than in c) or separate to separate fig, or even put in supplemental if there really is no difference

As suggested, we have modified **Figures 1b, c, h** to utilize broken axes.

5) I am slightly confused by the qPCR data showing that spic expression is down in ly6Chi mono, but not CD163 which is a spic-induced gene.

Similarly, what do you think is driving CD163 increase in the Ly6clo population where spic is unchanged, or maybe even down a little? The authors could discuss this more and speculate on on what this implies about the relationship between spic expression and CD163.

Indeed, in splenic Ly6C^{hi} monocytes, *Spic* was reduced but no changes were observed in *Cd163* expression. A reasonable explanation may be that *Cd163* expression is not exclusively dependent on *Spic* and is influenced by alternative signals. I. e. CD163 is known to be regulated by mutiple signaling pathways [11] and its expression might be maintained independent of *Spic*. Also, the majority of Ly6C^{hi} monocytes are not yet fully mature phagocytic cells that are capable of iron scavenging. However, our flow cytometry data (**Fig. 2c, i, l, o**) indicate that a small fraction of Spl Ly6C^{hi} monocytes (population #1) in the KO also upregulate CD163, though at the lower level as compared to the other subsets (populations #2, #3), which may additionally suggest that CD163 is regulated post-transcriptionally or that *Cd163* mRNA remain relatively stable and changes are not apparent in response to modest changes in *Spic* expression.

Considering the role of *Cd163* and *Hmox1* in heme scavenging and recycling, the observed upregulation of these genes in Ly6C^{lo} monocytes likely reflects a compensatory or a context-dependent response to RPM loss. Additionally, anti-Notch2 antibody treatment in wt mice was also sufficient to recapitulate the observed expansion of CD163 expressing cells in KO mice, supporting our hypothesis that this is likely a compensatory response to RPM loss (**Supplementary Fig. 5d-h**).

The modest downregulation of *Spic* is likely sufficient but not strictly necessary for the upregulation of *Cd163* in this monocyte subset. It is possible that *Cd163* and *Spic* interactions are indirect and is influenced by additional transcriptional and/or environmental

signals, in this case RPM loss and/or increased heme burden, rather than representing a direct regulatory pathway.

6) The IF images and videos are very nice, and qualitatively seem to confirm the quantitation from the flow, but when the macrophages are disorganized and spread out more, it is difficult to judge the abundance. I wonder if a volumetric analysis of the images could be done and provide an in situ confirmation of the trends observed in the flow (with respect to the macrophage populations) to exclude any potential artifacts or cell drop out due to preparation of the cells for flow.

As suggested, we quantified the percent of F4/80 positive area as well as intensity of F4/80 signal on 2D confocal images (**Supplementary Fig. 8a** in revised manuscript), which quantitatively confirm Notch2-dependent RPM loss in-situ. In our manuscript, we have provided multiple evidences of decreased RPM populations in the absence of Notch2, both quantitatively through: 1. flow cytometry in a) N2KO mice (**Fig. 1g, h**), or in b) wt mice treated with anti-Notch2 antibody (**Fig. 1i, j**), 2. single cell sequencing (**Fig. 3d**); and qualitatively through numerous confocal imaging experiments (**Fig. 4a, Supplementary Fig. 8a, b, d**) as well the provided videos (**Supplementary Videos 1 and 2**). Moreover, our repopulation experiments using Notch2-competent monocytes (**Fig. 5, Supplementary Fig. 12**), Notch2-competent and Notch2 KO bone marrow (**Fig. 6, Supplementary Fig. 13**) and fetal liver cells (**Supplementary Fig. 14**), where a clear rescue of the RPM populations are seen, confirm that the observed phenotype in N2KO mice are neither due to artifacts nor to cell drop out during cell preparation for flow cytometry but is indeed Notch2-dependent.

Reviewer #3 (Remarks to the Author):

Schroth et al. studied the role of Notch2 in the development and function of iron-recycling macrophages. The researchers showed that Notch2 depletion in Cx3cr1-positive macrophages led to a significant decrease in red pulp macrophages (RPM) and moderately decreased levels of bone marrow macrophages (BMM), which was followed by a late compensatory response expansion of CD163+ monocytes. However, these cells were not studied further. The lack of macrophages resulted in previously known iron accumulation in the spleen (e.g. PMID: 19037245, PMID: 38272969). The researchers also performed elegant adoptive transfer studies with total fetal liver and adult bone marrow cells. They showed the reconstitution of macrophages and the structure of the spleen but did not indicate, more specifically, the Notch2-dependent progenitor(s) that would be responsible for this reconstitution.

We thank the Reviewer #3 for their positive feedback and critical comments which we have addressed with additional and new experiments and provide our response here in a point by point fashion.

Our data support a requirement for Notch2 signaling in the development of RPM/BMM populations. In line with this, our repopulation experiments (adoptive transfer in newborns) with Ctrl or $N2^{\Delta Cx3cr1}$ BM or FL cells show that only Notch2-competent cells were able to reconstitute the defective niches and develop into RPM (**Fig. 5, 6, Supplementary Fig. 14**).

In separate studies using sorted BM $Ly6C^{hi}$ monocytes for adoptive transfer/repopulation (**Fig. 5i-p**), we show that these cells possess intrinsic ability to differentiate into RPM and therefore serve as precursor cells. Whether this is the scenario under steady-state conditions remains a question for our future studies.

Major:

1. A bar graph of the mean of data should be presented with overlapping dots for better individual observation of that variable and the real number of animals used in each experiment.

We have corrected all bar graphs in revised manuscript and addressed this point above (see our reply to Reviewer #1, Point 2).

2. The author should clearly indicate which experiments were repeated only twice. To ensure more reliable data, a minimum of three independent experiments should be conducted in the majority of experiments. It is also unclear if both male and female mice were used as a mix in the same experiments or if some experiments were only performed with one sex.

The figure legends have been updated to depict exact number of experiments performed. The majority of the experiments were performed with female mice except for repopulation experiments and those involving P0, P1 mice. Here, a mix of male and female recipients from complete litters were analyzed as it was not feasible to determine the sex of the mice at the time of injection or analysis. This approach reduced interexperimental variation potentially coming from different litters and hence kept the number of experimental animals

to a minimum. We have updated this information in the methods section of the main manuscript and is also provided in the Reporting Summary file.

CD45.1$^+$ BM repopulation studies (**Fig. 5a-f** in revised manuscript) were performed twice. We performed additional BM repopulation studies using mTmG mice (**Fig. 6a-f** in revised manuscript) which confirm the results obtained by CD45.1$^+$ BM repopulation. For this reason and to reduce the number of mice used to a minimum, we did not perform a third independent repopulation experiment with CD45.1$^+$ BM.

3. The FASC gating should be presented more clearly. For example, the gating for the Ly6Clo population, sorted in Figure 1, is not displayed in any of the gating strategies. In Figures 2a-c, splenic monocytes are gated from CD11b+ CD11c- cells, but this gating is not shown. Overall, the gating strategy is too complex, and it is difficult to understand which gating was applied to each experiment.

We have revised the main and supplementary figures, and the accompanying figure legends to improve clarity regarding the applied gating strategy. We have now included the sorting gating strategy (**Supplementary Fig. 3e** in revised manuscript) as well as the gating strategy for **Fig. 2a-c** (**Supplementary Fig. 5a-c**).

4. In Figure 1, algorithms like unsupervised k-means should be applied to the original high-dimensional data. t-SNE is primarily a visualisation tool and not ideal for clustering or quantitative analysis since it does not preserve real distances or densities. The analysis should also be done from single samples to see the variation in decreased cell numbers in different cell populations between the genotypes.

We have reanalyzed our data using the PhenoGraph plugin in Flowjo which "constructs a nearest-neighbor graph to capture the phenotypic relatedness of high-dimensional data points and then applies the Louvain graph partition algorithm to dissect the nearest-neighbor graph into phenotypically coherent subpopulations" [12]. The results are now depicted as such in **Fig. 1d-f**, and **Supplementary Fig. 2i-m.**

After analysis of concatenated data (using t-SNE and PhenoGraph plugins) we also constructed 2D tSNE maps of demultiplexed single samples with the cell counts of individual subpopulations which are now shown in **Fig. 5 for reviewers**.

5. The relative frequency or absolute numbers of each cell subset were calculated from the live cell gate and normalised per Spl. Even the spleens were different sizes? How was this considered in calculations?

We chose not to normalize by tissue weight (per mg Spl) particularly for newborn Spl (P0 and P1), as the Spl weight at this age is very low and difficult to measure. However, from 1 wk old onwards, we observed consistent, significant results regarding RPM loss that is independent of normalization method (% of live cells, per mg Spl or per Spl, **Fig. 6 for reviewers**). Therefore, we decided to present the data as relative frequency ('Cells (%)' calculated from live cell gate) or normalized per Spl, as normalization method does not alter our statement regarding Notch2-dependent RPM loss.

6. Figure 1, ``N2ΔCx3cr1 mice exhibited splenomegaly starting at 4 weeks (wk) of age but showed no changes in Spl total cell counts`` Why is spleen size growing but not total cell numbers? How was the data normalised?

It is likely that the compensatory shift in cellular composition observed in $N2^{\Delta Cx3cr1}$ mice accounts for the lack of detectable changes in total cellularity despite persistent splenomegaly. Splenomegaly is considered as one of the hallmarks of extramedually hematopoiesis in mice. The Spl of $N2^{\Delta Cx3cr1}$ mice show an expansion of $CD45^{neg}$ erythroid precursors midEB, lateEB (**Fig. 7a, b**), as well as numerous $CD71^{+}$ islands visualized in confocal images, further supporting its active extramedullary hematopoietic function and likely contributing to spleen enlargement. The increase in total iron and iron deposits in Notch2-deficient animals might also further contribute to splenomegaly.

In **Fig. 1c**, the depicted total cell numbers are normalized per Spl after RBC lysis, which removes the majority of erythrocytes and erythroid precursors. Analysis of total cell numbers in the Spl without lysis and normalized per Spl or per mg Spl, (**Fig. 7 for reviewers**) also show no significant increase in $N2^{\Delta Cx3cr1}$ mice.

[redacted]

[redacted]

[redacted]

Using models such as CX3CR1creERT2, Ms4a3cre/ERT2 mice, or RankCre, authors could point to the time but also the progenitor of Notch2-dependent RPM and BMM cells and provide evidence supporting the statement, "Several lines of evidence suggest a unique, but possible shared origin and developmental pathway of RPM and BMM"

Our statement was meant as an interpretation based on our data as well as on previous reports by other groups, for example Haldar, et. al [1]. Unfortunately, investigating and validating the potential shared ontogeny of RPM and BMM is beyond the scope of the present work. Nonetheless, we have analyzed additional mouse models, as suggested by the reviewer and have included the results in the main manuscript (**Supplementary Fig. 14m, n**).

[redacted]

[redacted]

[redacted]

8. Using a single-cell sequencing experiment to validate the finding in flow cytometry (Figure 3) is a bit confusing.

We have removed the statement "to validate the finding in flow cytometry" in the revised manuscript.

Was there a difference in the phenotype between monocytes or remaining RPM in Ctrl, N2ΔCx3cr1 and RbpjΔCx3cr1? Or are the expanding monocytes seen in N2ΔCx3cr1 mice similar in phenotype to those in the ctrl mice?

We have compared of Ly6C^{hi} (populations #1, 14, 15, 16 pooled), Ly6C$^{lo/int}$ (populations #9 and #11 pooled) and RPM (populations #10 and #18 pooled) populations between Ctrl and *N2*$^{\Delta Cx3cr1}$ and Ctrl and *Rbpj*$^{\Delta Cx3cr1}$ mice and depicted differentially expressed genes as volcano plots (**Fig. 3, Supplementary Fig. 6, 7**).

We observed considerable differences in the remaining RPM and expanded Ly6C$^{lo/int}$ subsets in both knock-out mice when compared with controls while Ly6C^{hi} monocytes showed relatively minor changes as shown in our DEG and GSEA analyses (**Fig. 3e-i, Supplementary Fig. 6, 7, in revised manuscript**).

In line with these data and in a separate experiment (**Fig. 9 for reviewers**), phagocytic activity of the remaining RPM in *N2*$^{\Delta Cx3cr1}$ seem to be diminished. In addition, the expanding monocytes in both *N2*$^{\Delta Cx3cr1}$ mice as well as in wt mice treated with Anti-Notch2 antibody express CD163 on their surface as compared to their Ctrl counterparts (**Fig. 2, Supplementary Fig. 5**).

Lastly, as we have shown in our recent paper [4], loss of Notch2 has broad effects on the development of Ly6C^{lo} monocyte subsets, reducing specifically the frequency of patrolling monocytes and expanding MHCII^{+} monocytes. Loss of Rbpj however, influences the phenotype of patrolling monocytes but has limited effect on the monocyte development.

9. Was the function of the remaining RPMs, such as phagocytosis, preserved in the N2ΔCx3cr1 model?

We performed in vitro culture and phagocytosis assay experiments to address this question.

We stained murine erythrocytes (RBC) with CFSE following a modified protocol [13, 14]. Briefly, following depletion of $CD45^+$ cells, RBC were stained with 2.5 µM CFSE. After thorough washing steps, a fraction of the cells was heat-stressed at 48°C for 30 min. Non-stressed RBC were incubated at 4°C for 30 min. Splenocytes from Ctrl or $N2^{\Delta Cx3cr1}$ mice were isolated and stained with the appropriate antibodies as described in the manuscript. Cells were coincubated with CFSE-labelled stressed or nonstressed RBCs at a 1:10 ratio for 1h at 37°C. Following brief lysis of remaining RBC using RBC-lysis buffer and extensive washing steps, Ctrl and $N2^{\Delta Cx3cr1}$ splenocytes were subjected to flow cytometry. Phagocytic activity was determined by measuring the frequency of $CSFE^+$ RPM or monocytes and the mean fluorescence intensity of CFSE in these cells (**Fig. 9 for reviewers**). We observed reduced phagocytic activity in the remaining RPM in the $N2^{\Delta Cx3cr1}$ mice (**Fig. 9a for reviewers**). In contrast, monocytes of $N2^{\Delta Cx3cr1}$ mice showed increased phagocytic activity, (**Fig. 9b for reviewers**), suggesting a compensatory mechanism to RPM loss.

Minor:

1. Ly6Clo (#5) monocytes (line 118) or CD43+Ly6Clo monocytes (#5) (line 120) ?

We have corrected Ly6Clo (#5), line 147 (revised manuscript) to $CD43^+Ly6C^{lo}$ monocytes, similar to line 150.

2. In figures, some more arrows would help the reader, such as in Figure 4, showing the disruption of MZ ring structure.

We have added more arrows to **Fig. 4** as suggested.

3. The results section sometimes overstretches into a discussion and can complicate the observations.

We have corrected the results section and moved all relevant topics to discussion.

References

1. Haldar, M., et al., *Heme-mediated SPI-C induction promotes monocyte differentiation into iron-recycling macrophages.* Cell, 2014. **156**(6): p. 1223-1234.
2. Gamrekelashvili, J., et al., *Notch and TLR signaling coordinate monocyte cell fate and inflammation.* Elife, 2020. **9**.
3. Yona, S., et al., *Fate mapping reveals origins and dynamics of monocytes and tissue macrophages under homeostasis.* Immunity, 2013. **38**(1): p. 79-91.
4. Xu, Y., et al., *Systematic monocyte subset analysis reveals differential contribution of Notch signaling components to monocyte heterogeneity.* iScience, 2025.
5. Koh, B.I., et al., *Adult skull bone marrow is an expanding and resilient haematopoietic reservoir.* Nature, 2024.
6. Gamrekelashvili, J., et al., *Regulation of monocyte cell fate by blood vessels mediated by Notch signalling.* Nat Commun, 2016. **7**: p. 12597.
7. Kohyama, M., et al., *Role for Spi-C in the development of red pulp macrophages and splenic iron homeostasis.* Nature, 2009. **457**(7227): p. 318-21.
8. Okreglicka, K., et al., *PPARgamma is essential for the development of bone marrow erythroblastic island macrophages and splenic red pulp macrophages.* J Exp Med, 2021. **218**(5).
9. Chen, L., et al., *Dynamic changes in murine erythropoiesis from birth to adulthood: implications for the study of murine models of anemia.* Blood Adv, 2021. **5**(1): p. 16-25.
10. Chow, A., et al., *Bone marrow CD169+ macrophages promote the retention of hematopoietic stem and progenitor cells in the mesenchymal stem cell niche.* J Exp Med, 2011. **208**(2): p. 261-71.
11. Etzerodt, A. and S.K. Moestrup, *CD163 and inflammation: biological, diagnostic, and therapeutic aspects.* Antioxid Redox Signal, 2013. **18**(17): p. 2352-63.
12. Levine, J.H., et al., *Data-Driven Phenotypic Dissection of AML Reveals Progenitor-like Cells that Correlate with Prognosis.* Cell, 2015. **162**(1): p. 184-97.
13. Willingham, S.B., et al., *The CD47-signal regulatory protein alpha (SIRPa) interaction is a therapeutic target for human solid tumors.* Proc Natl Acad Sci U S A, 2012. **109**(17): p. 6662-7.
14. Xu, C., et al., *Protocol for detecting macrophage-mediated cancer cell phagocytosis in vitro and in vivo.* STAR Protoc, 2023. **4**(1): p. 101940.

[redacted]

[redacted]

[redacted]

[redacted]

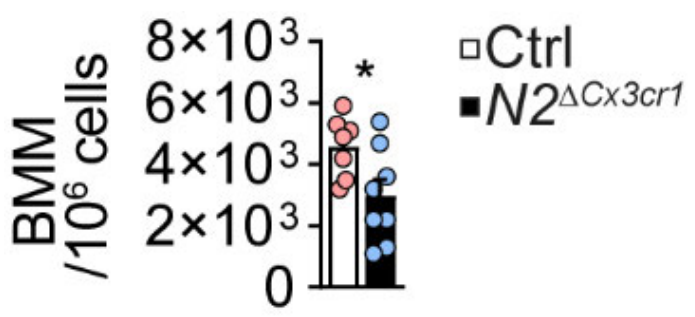

Fig. 2. BMM content, normalized per 10^6 cells. Normalization of BMM cells per 10^6 total viable BM cells. Data are mean ± SEM and pooled from three independent experiments. *P<0.05; **P<0.01; ***P<0.001; (Student's t-test). n = 7/8. Related to **Fig. 1r** in the revised manuscript.

[redacted]

[redacted]

[redacted]

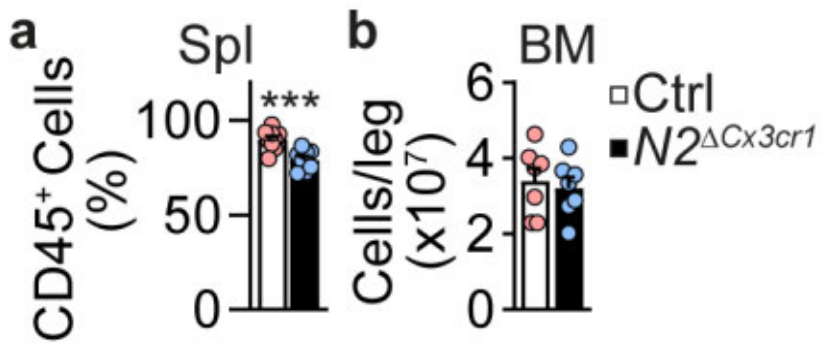

Fig. 4. Comparison of CD45$^+$ splenocytes and total BM cellularity. (**a**) frequency of CD45$^+$ cells (calculated from live cell gate) are decreased in *N2*$^{\Delta Cx3cr1}$ mice (n = 9/8) while (**b**) total cellularity in the BM (normalized per leg) is not significantly different between genotypes (n = 7/7). (**a-b**) Data are mean ± SEM and pooled from three experiments. *P<0.05; **P<0.01; ***P<0.001; (Student's t-test).

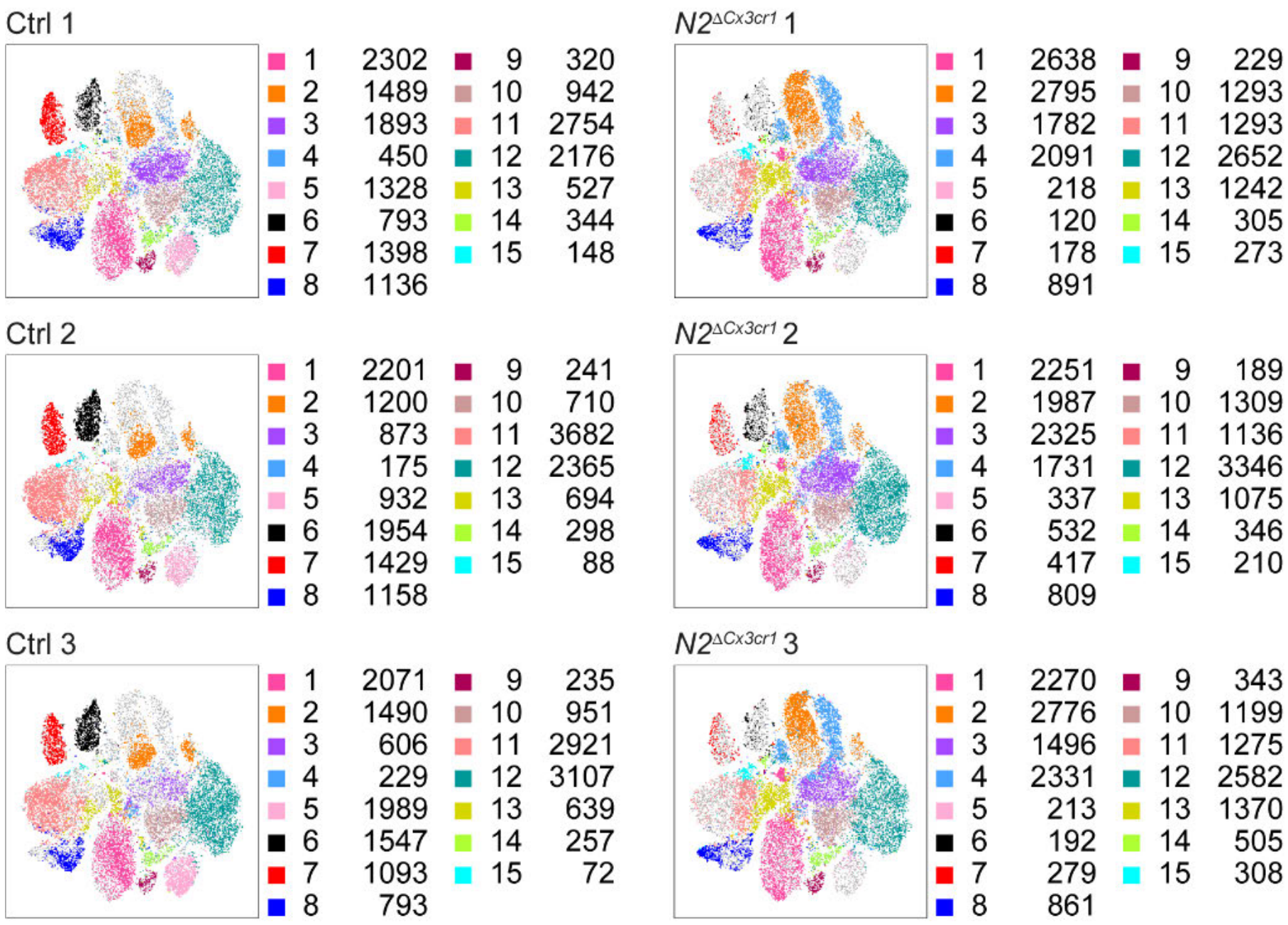

Fig. 5. tSNE maps for individual samples. tSNE maps of individual samples are shown after identification of subsets (#1-15) using PhenoGraph and demultiplexing of concatenated data. Related to **Fig. 1f** in the revised manuscript.

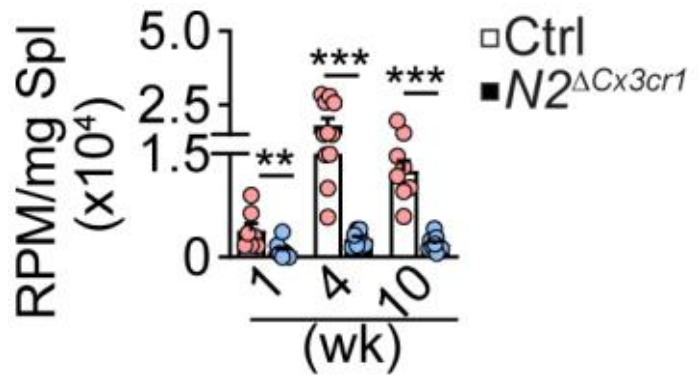

Fig. 6. Normalization of RPM number per mg Spl. Normalization of RPM number per mg Spl (x10^4) yields comparable significant results to normalization of RPM cells per Spl (**Fig. 1h** in revised manuscript). Data are mean ± SEM and pooled from three independent experiments. *P<0.05; **P<0.01; ***P<0.001; (Student's t-test). 1w: n = 7/5, 4w: n = 11/8, 10w: n = 8/9.

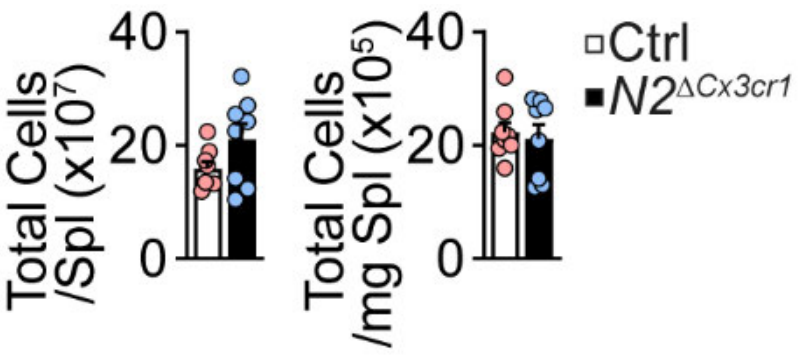

Fig. 7. Total cells per Spl (without lysis) or total cells per mg Spl (without lysis) are not increased in *N2*$^{\Delta Cx3cr1}$ mice. Bar graphs depict total cell counts normalized per Spl or per mg Spl. Data are mean ± SEM and pooled from three experiments. *P<0.05; **P<0.01; ***P<0.001; (Student's t-test). n = 8/8.

[redacted]

[redacted] [redacted]

[redacted]

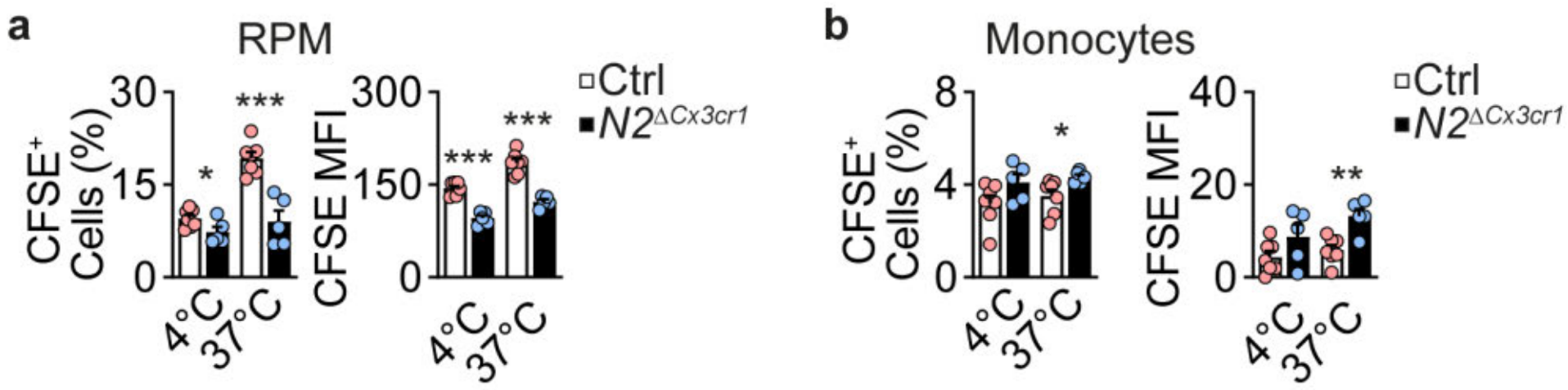

Fig. 9. Decreased phagocytic activity in Notch2-deficient RPM. (**a**) Relative frequency (from live cell gate) of CFSE^{+} RPM cells (left panel) and MFI of CFSE (right panel) within the RPM population *in vitro* in Ctrl or *N2*$^{\Delta Cx3cr1}$ mice. RPMs were defined as Live(PI^{neg})CD45^{+}Lin$^{lo/-}$CD117^{neg}F4/80^{hi}Ly6C$^{lo/-}$I-A/I-E$^{lo/-}$CD11c$^{lo/-}$CD11b$^{lo/-}$CD43$^{lo/-}$cells. (**b**) Relative frequency (from live cell gate) of CFSE^{+} Monocytes (left panel) and MFI of CFSE (right panel) within the monocyte population *in vitro* in Ctrl or *N2*$^{\Delta Cx3cr1}$ mice. Monocytes were defined as Live(PI^{neg})CD45^{+}Lin$^{lo/-}$CD117^{neg}CD11b^{+}F4/80$^{lo/-}$I-A/I-E$^{lo/-}$CD11c$^{lo/-}$ cells. (**a, b**) Data are mean ± SEM and pooled from three experiments. *P<0.05; **P<0.01; ***P<0.001; (Student's t-test). n = 7/5.

REVIEWER COMMENTS

Reviewer #1 (Remarks to the Author):

General Comments

The revised manuscript by Schroth et al. has been significantly improved. The authors have rigorously addressed the concerns raised during the first round of review, particularly regarding the potential for Cre-mediated off-target effects. By incorporating comprehensive genetic controls (Cx3cr1+/+ Notch2f/f, Cx3cr1+/+ Notch2+/+, Cx3cr1Cre/+ Notch2+/+) and utilizing an anti-Notch2 antibody in wild-type mice, the study now convincingly demonstrates that the loss of canonical Notch2-Rbpj signaling directly disrupts red pulp macrophages (RPM) [redacted]

The manuscript provides valuable mechanistic insights into macrophage ontogeny and iron homeostasis. The elegant adoptive transfer models (CD45.1+ bone marrow, fetal liver cells, and mTmG) confirm that the reconstitution of the defective splenic niche is strictly dependent on Notch2-competent progenitors. Furthermore, the observation that Notch2 deficiency triggers a local compensatory expansion of CD163+ Ly6Clo/int monocytes and confers an initial adaptive advantage during PHZ-induced hemolytic anemia highlights a highly dynamic cross-talk between macrophage niches and extramedullary hematopoiesis. The findings are novel and carry substantial translational potential for manipulating iron-recycling macrophages in vascular and hemolytic diseases.

While the primary conclusions are now exceptionally well-supported, there are two remaining points that require the authors' attention prior to final acceptance.

We thank the reviewer for their constructive response and for acknowledging our efforts to improve the quality of the manuscript.

Specific Comments

Inclusion of Functional Assays: In the rebuttal, the authors provided a CFSE-labeled erythrocyte phagocytosis assay demonstrating reduced phagocytic activity in residual Notch2-deficient RPMs and a compensatory increase in monocytes. This is a crucial piece of functional evidence. If this assay is solely confined to the response letter, the authors must either incorporate it into the main manuscript (or supplementary materials) or temper their claims regarding Notch2's regulation of macrophage "function" in the main text.

We agree with the reviewer and have included this data in the manuscript (**Supplementary Fig. 5e, f**) as well as in the accompanying text in Results (Line 209-212) and Methods (Line 990-1000).

scRNA-seq Statistical Rigor: The single-cell transcriptomic data provides robust molecular confirmation of the flow cytometry results. However, given that the design multiplexes two mice per genotype onto a single 10x lane, the analytical approach needs further clarification. The authors must detail their QC thresholds, HTO demultiplexing criteria, and doublet handling strategies. More importantly, differential expression

analysis (DEG) and pathway enrichment must be performed using a replicate-aware or pseudobulk framework to avoid statistical inflation. If conventional cell-level statistics were used, the transcriptomic conclusions should be explicitly framed as exploratory and supportive rather than definitive.

As requested, we updated the Methods section to include the detailed QC thresholds, HTO demultiplexing criteria and doublet handling strategies for our scRNA-seq analysis (Line 947-966).

Since we had two mice per group in our scRNA-seq analysis, we had initially meant the analyses to be exploratory and confirmatory to the results obtained from other experimental approaches and to the conclusions reached based on these approaches. However, as we were requested during the revision to provide more advanced analysis of scRNA-seq data, we performed the additional analyses and modified the manuscript accordingly.

We left these modified analyses in the revised manuscript but modified the text, the title of the respective paragraph and figure headings (main and supplementary figures) to reflect the exploratory and supportive nature of the analyses. In addition, we explicitly indicated in the Methods section that conventional cell-level statistics were used for the DEG and pathway enrichment analyses (Line 974). We have now made the necessary text modifications to indicate the exploratory nature of the DEG and pathway enrichment analyses and tempered our statements in the Results section (Line 232-297, in red).

Recommendation:

Accept with Minor Revisions. (The authors have thoroughly addressed the majority of previous critiques, resulting in a robust manuscript; addressing the two minor methodological points above will finalize the work for publication).

We thank the reviewer for this recommendation.

Reviewer #2 (Remarks to the Author):

I commend the authors on the additional data provided which has at least attempted to address the reviewer concerns. The presentation of the data is improved with respect to transparency of individual values and number of replicates and important controls have been added. However, in some cases I feel the authors may have misunderstood the intention of the reviewer comments, and in many cases, the additions of figures or text do not improve the clarity of the data or the manuscript. I will concede that this may merely be a stylistic preference and defer to the authors if they are happy with the manuscript in its current form. I have listed a few minor comments for their consideration below:

We thank the reviewer for their assessment of the revised manuscript and for acknowledging the improvements in data presentation with respect to transparency of individual values, number of replicates, and important controls.

Where the reviewer feels that we may have misunderstood the intention of certain reviewer comments, we respectfully note that we interpreted the original concerns to the best of our understanding and implemented our revisions accordingly.

While we recognize that alternative approaches to data presentation are possible, we believe that the changes we made improve the clarity and strength of the data and thereby back the statements made in this manuscript. We appreciate the reviewer's acknowledgment that the remaining concerns simply reflect differences in presentation preference, which we address in more detail below (**Point 4**).

Major

1) The authors added some lovely validation data showing that antibody-mediated blocking of notch2 can phenocopy the loss of RPM observed in Notch2-deleted animals however, this is a little confusing and requires better explanation in the text.

a. In the text they state "treatment of adult wild-type (wt) mice with anti-Notch2 (aN2) antibody, but not control antibody (Ctrl-Ab), was sufficient to phenocopy…" (lines 165-166), but this would demonstrate that notch2 is also required for maintenance of the RPM pool in adults, rather than a role in development. But,

b. In the methods (supplemental, line 1003), it states that anti-Notch2 and control antibodies were injected at 20mg/kg per mouse in PBS on days 0 and 7" which is much more appropriate for demonstrating a role in development.

This obviously requires clarification.

We suspect this is a misunderstanding coming from our description of the anti-Notch2 (anti-N2) antibody treatment. In the Methods section, days 0 and 7 refer to the time points of antibody administration to adult animals rather than to the age of the animals. We have now modified it to explicitly say that the treatment was done in adult mice and that days 0 and 7 refer to treatment time points (Line 1046). This protocol was adapted from Cruz Tleugabulova et. al (2024) [1], where they performed two injections of Ctrl or anti-N2 antibodies (adult mice on days 0 and 7) and analyzed the effects two weeks after the initial injection.

Although we do not believe the reviewer was necessarily requesting additional experiments in P0/P7 mice, we provide the following response in case this was the intended suggestion: Considering that anti-N2 treatment also alters the established RPM pool, the proposed experiment (treatment of P0/7 mice with anti-N2 antibody) will not answer the development vs maintenance question, because in this scenario, the experimental endpoint is a reduction in RPM numbers. Such a reduction could arise from impaired differentiation of potential progenitor/precursor into RPM or from compromised maintenance of cells that have already acquired the RPM phenotype. Since anti-N2 treatment at P0/P7 could potentially affect both processes simultaneously, it would be challenging to determine whether the observed RPM reduction reflects a defect in RPM development, maintenance, or both.

Having this in mind, when adopting this experiment, we did not aim to answer development vs maintenance questions but aimed to see **a**) whether targeting of Notch2 in a Cre-independent manner has any effect on RPM, and **b**) to confirm the phenotype [redacted] observed in Notch2-deficient mice as requested by the editor and reviewers. We indeed showed that Notch2 is also required for the maintenance of the RPM pool.

At the same time, we have shown that the acquisition of RPM identity (specifically, expression of *Slc40a1*, *Spic*, *Nr1h3*, *Hmox1*) in vitro (**Fig. 6i-n**) was dependent on Notch2 signaling, confirming the role of this signaling cascade in macrophage development. Identification of the exact molecular mechanisms by which Notch2 regulates the development (differentiation of progenitor/precursor cell into RPM) and maintenance of RPM identity, and its interaction with other known (Pparg, Spic, Bach1) or novel signaling molecules is a matter for further studies.

To emphasize these details in the manuscript, we modified the title of the manuscript, "Canonical Notch2 Signaling Regulates Development **and Maintenance** of Iron-recycling Macrophages and Iron Homeostasis" and also included respective clarifications in the text.

2) I appreciate the authors including the requested FMOs, which are meant to serve as guides for the setting gates on true positive events. What is the interpretation of the high % positive in the FMOS for CD169 and TIM4 (6.5% and 3.4% respectively, supp fig 3b). If 6.5% positive is the background for CD169, what then does 9% positive in the WT really mean?

We would like to clarify that the sample depicting a 9.16% of the parental population corresponds to a Notch2-KO sample (not a WT as is stated in the reviewer's comment). Likewise, the FMOs for CD169 and Tim4 were derived from the same Notch2-KO sample. These FMOs served as a guide for gate placement and were applied to all samples. The percentages in this case represent the background signal attributable to anti-Tim4 staining (6.51%, FMO for CD169) and anti-CD169 staining (3.41%, FMO for Tim4). In contrast, the actual sample itself contains 9.16% of the population within the gated region. Application of the same gate on all samples did not alter the conclusion that the relevant cell population is indeed expanded in KO mice compared to Ctrl.

To independently confirm our statement that we have expansion of CD169^{+} cells, we performed a separate staining where we excluded anti-Tim4 and used a different fluorochrome-labelled anti-CD169 antibody (**Fig. 1** for Reviewers) which confirm results shown in **Fig. 1k** and **Supplementary Fig. 3b**.

Importantly, these results are also confirmed by confocal microscopy (**Fig. 4**), which clearly demonstrates massive CD169^{+} signal in the RP of Notch2-KO mice.

3) line 448 references supp fig 14, but I believe this should be 15

Yes, this should be **Supplementary Fig. 15** and we corrected this accordingly.

Minor points

4) As the characterization of the phenotype relies heavily on abundance of particular cell populations, I find the continued reliance on relative frequence, or % of parent or live cell population, difficult to interpret. I concede that in some cases, this can accurately reflect the changes that are occurring, but also note that care must be taken in the interpretation of these because they are indeed "relative" and can be misleading depending on the circumstances, especially when there are changes in overall cellularity or abundance of the parent population. Absolute numbers would always be preferred and unless it serves some purpose, I see no reason to include both as in Fig 1.

We thank the reviewer for their awareness of the types of analysis, and we agree with them. However, in our previous studies published in the past 10 years, we were requested to provide both relative and absolute quantification of cells [2-7]. This approach is now established as a standard in our group and is well accepted by other researchers in the field, including Reviewer #1 and Reviewer #3 for the current manuscript.

We are, of course, well aware that overall cellularity and the abundance of the parent populations can influence the interpretation of the data. For this exact reason, we provided both analyses and show that they do not contradict each other but rather confirm the conclusions made in this study.

5) In the tsne clustering in Fig 1, it is not clear why only clusters 1-7 are highlighted and quantified, especially when #5,6,7, and 8 were decreased in the N2 KO (line 149-150), so 8 at least should also be labeled and quantified in the main figure. Why only the first 7?

Since our aim was to focus on RPM and expanded monocyte populations and to reduce the complexity of the figure, we highlighted only clusters 1-7 in the main figure. However, the complete quantification for all identified clusters is also presented in **Supplementary Fig. 2j-m**.

As regard to the other populations: The role of Notch in the development of patrolling monocytes, specifically the reduction of $CD43^{+}Ly6C^{lo}$ monocytes (Population #5) upon Notch2 deletion, has been previously described by our group [2, 5] and was therefore not further described in this study. Populations #6 and #7 are $F4/80^{+}$ RPM and are labelled and quantified in the main figure (Line 149, **Fig. 1f**).

In the initial submission, population #8 was designated as $CD11c^{hi}CD11b^{+}I\text{-}A/I\text{-}E^{+}$ DC. In the current version, which relies on Phenograph-based clustering, this DC population, which is reduced in Notch2-deficient mice, is designated as population #11 (Line 150). We corrected the numbering of the populations in the text accordingly. The reduction of this splenic DC subset (cDC2) in Notch2-deficient mice has already been shown [8-11] and therefore not further described in this study.

6) The authors state that residual RPM in αN2-treated wt mice displayed increased CD163 surface expression, as reflected by the increased CD163 MFI. (supp fig 5d-h) (line 213). In Supp Fig 5h, the rpm population is bimodal for CD163 and there appears to be an increase in the number with the higher expression. Could the authors clarify if the actual mfi of that population is increased relative to the same pop in WT or is the overall mfi is just higher because there more are in the CD163 high pop relative to the lo pop?

The MFI within the gated $CD163^{+}$ RPM population is indeed increased, compared to the same population in Ctrl-treated mice. The increased MFI is not solely due to increased frequency of CD163-expressing cells.

7) CD163 is not mentioned in the section on the transcriptomics differences in RPM and Ly6Clo monos, despite the previous paragraph being about increased CD163 expression...it is not shown in the bubble plot (3c, or any of the volcano plots 3e-g) nor is it identified as differentially expressed in most of the comparisons. Protein does not always = transcript and there may be many explanations for this, but the authors should at least mention this disparity rather than avoid it.

We thank the reviewer for this comment and have added a short statement regarding the apparent disparity in the discussion section (Line 568-572).

Notably, this gene was downregulated in RBPJ-KO RPM as described in the results (Line 271 and volcano plot in **Supplementary Fig. 7a**), whereas it was not significantly changed or detected in the Notch2-KO scRNA-seq dataset, likely reflecting the lower sensitivity of scRNA-seq for detecting modest expression changes.

However, to strengthen our flow cytometry-based analysis and provide independent confirmation of the observed phenotype at the transcript level for Notch2-KO, we employed a more sensitive qRT-PCR approach and performed analysis on sorted RPM and $Ly6C^{hi}$ and $Ly6C^{lo}$ monocyte subsets from Ctrl or Notch2-KO mice. The results of this study are described in **Fig. 1 n-p**. These data show that overall *Cd163* is highly expressed in RPM as compared to monocytes, independent of genotype; however, Notch2-KO RPMs show a statistically significant reduction of *Cd163*, while $Ly6C^{lo}$ monocytes show upregulation of it as compared to wt controls.

8) Much of the added transcriptomic analysis does not contribute much to the overall conclusions of the paper. The authors could really just hit the highlights in a quick summary and move the rest of the details to the legend of the supp figure.

In the initial submission, we had a relatively brief description of the transcriptomic analysis, and depicted it as confirmation of other analyses we had performed. However, we were requested to perform a more in-depth and extended analysis of the transcriptomic data (See also response to reviewer No1 and 3). To accommodate all reviewers' suggestions regarding this particular figure, we kept the corresponding figures in their current format, but have now streamlined the corresponding text, as suggested.

9) The transplant experiments are nice, but not well explained. If "neither BM nor Spl cell transfer rescued BMM" (line 348) then why did "Transfer of the ctrl mG BM cells did rescue the BMM" (line 385). I searched for details if these were GFP+ sorted or something to explain the difference, but appeared to be total bone marrow? Why the difference in rescue?

We would like to point out that we did observe a slightly higher number of BMMs (albeit not statistically significant) after transfer of $CD45.1^+$ BM cells, and when analyzed, around 28% of recipient BMMs showed donor origin.

In the initial repopulation experiments with $Ly5.1^+$ BM, donor cells were injected in P7 (on day 7 of age) recipients, whereas subsequent transfers using mTmG BM were performed in P5 recipients. By P7, donor cells could encounter a more developed recipient BM niche, and this could have affected efficient engraftment as the cells need to compete with endogenous BM cells for the niche.

Another possibility is that, although donor mice are maintained on the same BL6 background, subtle strain- or colony-specific differences could still influence the number or the homing/repopulation ability of potential progenitors. Our CD45.1 mice (B6.SJL-PtprcaPepcb/BoyJ) are on 'J background' while the Ctrl *mG*, KO *mG* donors as well as recipients are on 'NRJ background'. Furthermore, we cannot exclude the possibility of technical variability during BM isolation, cell counting and injection that may have resulted in a subtle difference in the number of BM progenitor cells transferred between these experiments.

Consequently, this particular data should be interpreted as supportive rather than conclusive, whereas the parallel Ctrl vs Notch2-deficient BM and FL transfer provides stronger evidence for Notch2-dependent BMM reconstitution.

10) If I understand the flow gating figures correctly, the same gating strategy was used for BMM as for RPM, meaning all CD11b-/CD11c- cells were excluded. The authors should take note that there is a macrophage population in the murine bone marrow which is Cd11b- that is involved in the erythropoietic niches which is not quantified by their analysis. [redacted] **To be clear, I am not asking the authors to perform additional analyses, but rather to acknowledge the limitations of their bone marrow macrophage definition and the possibility that other niche macrophages may or may not play a role in their phenotype**.

We recognize the reviewer's concern, which we agree with, and have therefore added an additional paragraph acknowledging this limitation to the discussion of the manuscript (Line 639).

We applied the same gating strategy for the BMM definition and analysis, which excludes $CD11b^{neg}/CD11c^{neg}$ (double-negative, but not $CD11b^{neg}CD11c^+$ cells) from the analysis. Of course, we completely agree with the reviewer that other subsets ($F4/80^{lo/neg}$) of BMM may also play the critical role in the establishment and maintenance of the erythropoietic niche in the BM and (indirectly) contribute to the phenotype observed in our studies. [redacted]

[redacted]

We also agree that further in-depth studies are needed for the definition of BMM subsets and delineation of their function in erythropoiesis and BM-niche maintenance, but this lies outside the scope of this manuscript.

[redacted]

Reviewer #3 (Remarks to the Author):

I have reviewed the revised manuscript by Schroth et al. The authors have adequately addressed most of my comments through revisions to the text and the inclusion of additional experimental evidence. The revised article is clearly improved. I have no further comments.

We thank the reviewer for acknowledging our efforts to answer all questions/concerns raised by the reviewers and to improve the quality of the manuscript.

Figures for Reviewers

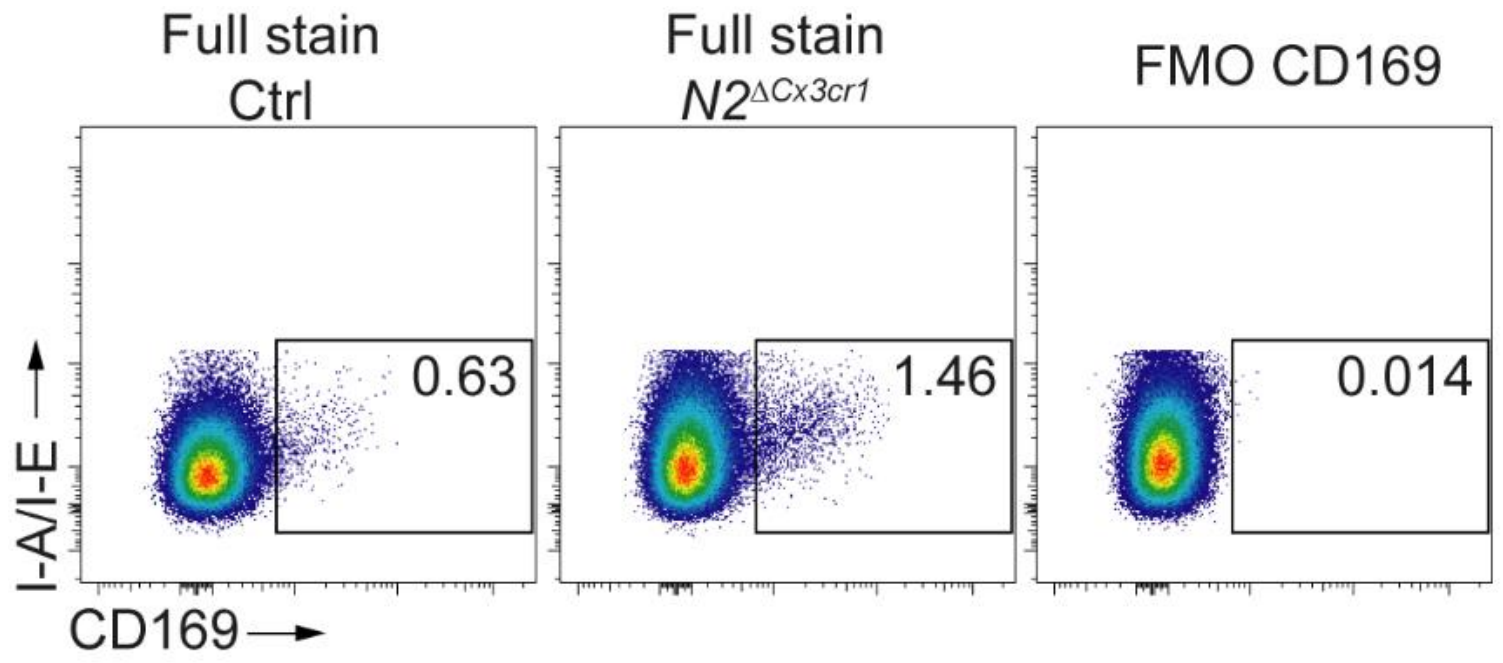

Fig. 1. Additional FMO Control for CD169 without anti-Tim4. Values indicate frequency of parent population. Upstream gate as in **Supplementary Fig. 3a.**

References

1. Cruz Tleugabulova, M., et al., *Induction of a distinct macrophage population and protection from lung injury and fibrosis by Notch2 blockade.* Nat Commun, 2024. **15**(1): p. 9575.
2. Gamrekelashvili, J., et al., *Regulation of monocyte cell fate by blood vessels mediated by Notch signalling.* Nat Commun, 2016. **7**: p. 12597.
3. Krishnasamy, K., et al., *Blood vessel control of macrophage maturation promotes arteriogenesis in ischemia.* Nat Commun, 2017. **8**(1): p. 952.
4. Gamrekelashvili, J., et al., *Notch and TLR signaling coordinate monocyte cell fate and inflammation.* Elife, 2020. **9**.
5. Xu, Y., et al., *Systematic monocyte subset analysis reveals differential contribution of Notch signaling components to monocyte heterogeneity.* iScience, 2025.
6. Kapanadze, T., et al., *CSF-1 and Notch signaling cooperate in macrophage instruction and tissue repair during peripheral limb ischemia.* Front Immunol, 2023. **14**: p. 1240327.
7. Kapanadze, T., et al., *Multimodal and Multiscale Analysis Reveals Distinct Vascular, Metabolic and Inflammatory Components of the Tissue Response to Limb Ischemia.* Theranostics, 2019. **9**(1): p. 152-166.
8. Caton, M.L., M.R. Smith-Raska, and B. Reizis, *Notch-RBP-J signaling controls the homeostasis of CD8- dendritic cells in the spleen.* J Exp Med, 2007. **204**(7): p. 1653-64.
9. Lewis, K.L., et al., *Notch2 receptor signaling controls functional differentiation of dendritic cells in the spleen and intestine.* Immunity, 2011. **35**(5): p. 780-91.
10. Satpathy, A.T., et al., *Notch2-dependent classical dendritic cells orchestrate intestinal immunity to attaching-and-effacing bacterial pathogens.* Nat Immunol, 2013. **14**(9): p. 937-48.
11. Minutti, C.M., et al., *Distinct ontogenetic lineages dictate cDC2 heterogeneity.* Nat Immunol, 2024. **25**(3): p. 448-461.
12. Seu, K.G., et al., *Unraveling Macrophage Heterogeneity in Erythroblastic Islands.* Front Immunol, 2017. **8**: p. 1140.
13. Chow, A., et al., *CD169(+) macrophages provide a niche promoting erythropoiesis under homeostasis and stress.* Nat Med, 2013. **19**(4): p. 429-36.

REVIEWERS' COMMENTS

Reviewer #1 (Remarks to the Author):

I consider this to be a well-developed study with a clear rationale and a relatively complete body of data, and I recommend publication after minor revision. The manuscript focuses on the role of canonical Notch2-RBPJ signaling in the development, maintenance, and iron homeostatic function of iron-recycling macrophages, including RPM and BMM. By combining genetic models, antibody-mediated blockade, single-cell transcriptomics, tissue imaging, iron quantification, adoptive transfer experiments, and in vitro induction assays, the authors establish a coherent line of evidence.

The authors show that conditional deletion of Notch2 or its downstream mediator Rbpj leads to a marked reduction of splenic RPM and bone marrow BMM, accompanied by iron accumulation in the spleen and bone marrow, downregulation of RPM signature genes, expansion of CD163+ monocytes, and enhanced extramedullary hematopoiesis. Anti-Notch2 antibody treatment in adult wild-type mice recapitulates part of the phenotype, suggesting that Notch2 is involved not only in development but also in the maintenance of the mature iron-recycling macrophage pool. The in vitro DLL1-hemin experiment further links Notch2 signaling to the heme-induced RPM differentiation program. Compared with previous studies implicating Spic, Pparg, Hmox1, CSF-1, and IL-33 in RPM development and function, this study incorporates Notch2-RBPJ signaling into this regulatory network and provides a new mechanistic connection.

We thank Reviewer #1 for their recommendation for publication and their additional comments, which we address in detail below.

The following points could be improved:

1. Individual conclusions should be slightly tempered. For example, the statement “Notch2 deficiency confers improved adaptation to hemolytic anemia” could be revised to “is associated with an altered early response to PHZ-induced hemolytic anemia.” This result is based on early observations in a specific acute hemolysis model and should not be overinterpreted as indicating that Notch2 deficiency is generally beneficial.

We thank the reviewer for this comment and suggestion. We have modified our statements, as suggested. See Lines #474, #515, #622-623, #1356-1357 as well as the figure title in the accompanying Supplementary Figure (**Supplementary Fig. 17**)

2.The interpretation of the single-cell transcriptomic and pathway enrichment results should remain cautious in the Discussion. Some enriched pathways, such as metabolic, inflammatory, and mitochondrial pathways, are relatively broad. The authors should emphasize that these analyses mainly support and help interpret the observed phenotypes, rather than serving as standalone mechanistic conclusions.

We agree with the reviewer and have omitted a discussion of scRNA-seq data from the Discussion section. Similarly, in the Results section, we have specifically stated that scRNA-seq is exploratory and supports the observed phenotypes (Lines #219, 292)

3. The authors may consider adding or improving a summary model figure to help readers understand the relationships among Notch2-RBPJ, DLL1/heme, RPM/BMM, iron homeostasis, and extramedullary hematopoiesis.

We provide in **Supplementary Fig. 18** a graphical summary of this study.

Reviewer #2 (Remarks to the Author):

No further comments.
