# Supplementary material for: Canonical Notch2 signaling regulates development and maintenance of iron-recycling macrophages and iron homeostasis
Source: Nat Commun. 2026 Sep 23;17:10132. doi: 10.1038/s41467-026-77938-7 (PMC13601512; doi:10.1038/s41467-026-77938-7)
Supplement: Supplementary file 3 [file 41467_2026_77938_MOESM1_ESM.pdf]

Supplementary Information File

Canonical Notch2 Signaling Regulates Development and Maintenance of Iron-recycling Macrophages and Iron Homeostasis

Frauline Nicole Schroth[1,2,3], Tamar Kapanadze[1,2], Stefan Sablotny[1,2], Yuangao Xu[1,2], Alessia Ascierto[4], Matthias Ballmaier[5], Katharina Mauel[6], Bo Mee Chung[7], Lena Deuper[8], Andrey S. Shaw[9], Andreas Kispert[8], Matthias Lochner[10], Tibor Kempf[7,11], Elvira Mass[6], Hermann Haller[3], Kai M. Schmidt-Ott[2], Jaba Gamrekelashvili[1,2,*,†], Florian P. Limbourg[1,2,*,†]

Supplementary Fig. 1-18

Supplementary Table 1, 2

Uncropped images of Gel, related to Supplementary Fig. 1a, b

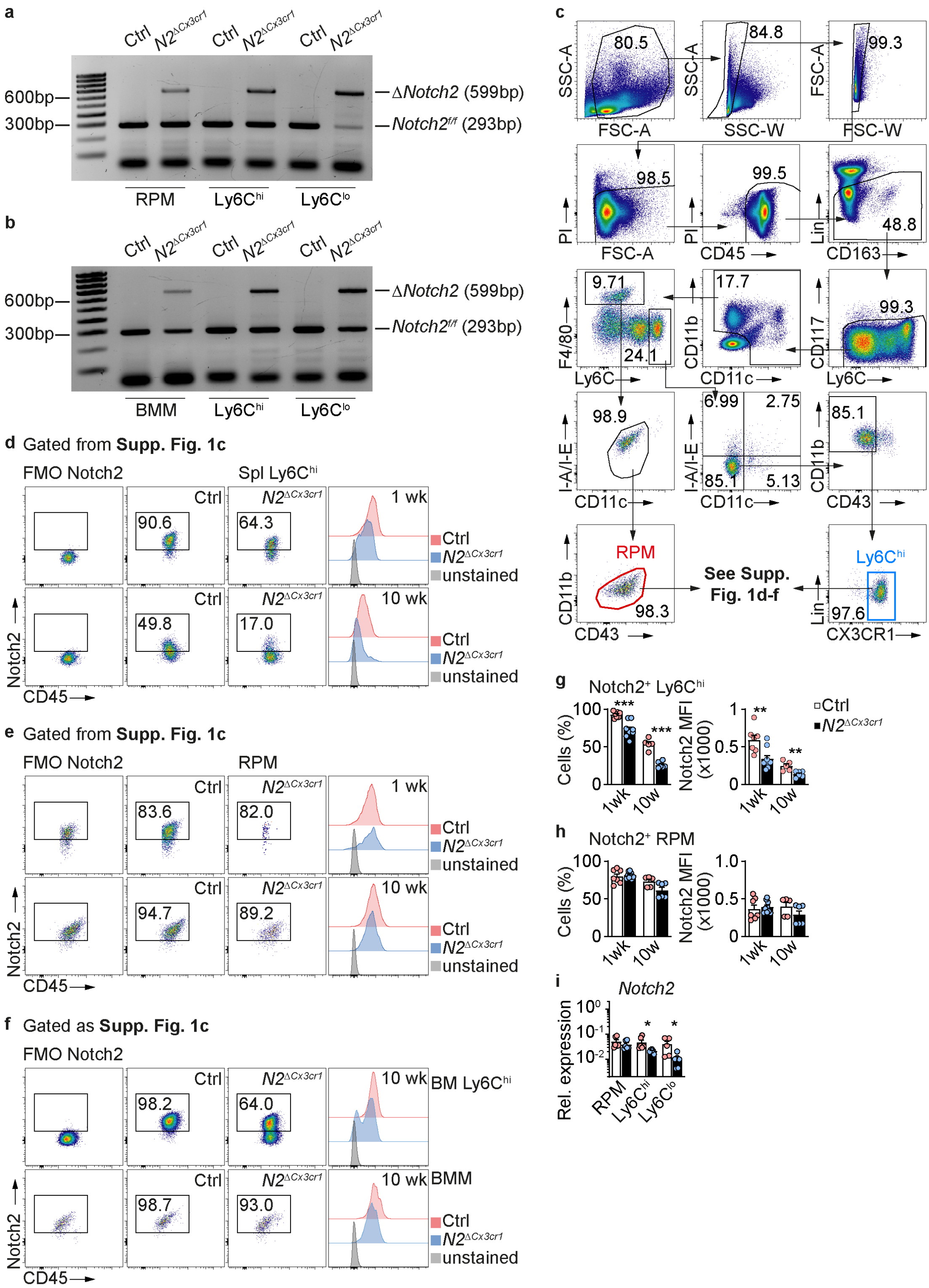

Supplementary Fig. 1. Validation of Notch2 deletion in the *N2$^{\Delta Cx3cr1}$* knockout mouse model. (**a-b**) PCR and gel-electrophoresis of sorted RPM, BMM, Ly6C^{hi} and Ly6C^{lo} monocytes from the Spl (**a**) and BM (**b**) showing the floxed (*Notch2$^{f/f}$*, 293 bp) and recombined alleles (*ΔNotch2*, 599 bp) in Ctrl or *N2$^{\Delta Cx3cr1}$* mice. (**c**) Gating strategy for identification of Notch2 staining in splenic RPM and Ly6C^{hi} monocyte populations. (**d, e**) Representative flow cytometry plots and histograms depicting Notch2 staining in Spl Ly6C^{hi} monocytes (**d**) and RPM (**e**) from 1wk- and 10wk old Ctrl and *N2$^{\Delta Cx3cr1}$* mice. Values depicted

are frequency of parent population. FMO for Notch2 served as an additional control for staining and gating. Gated from **Supplementary Fig. 1c**. (**f**) Flow cytometry plots and histograms showing Notch2 staining in BM Ly6C^{hi} monocytes (upper panel) and BMM (lower panel) from 10wk old Ctrl and *N2*$^{\Delta Cx3cr1}$ mice. Gating strategy is similar to **Supplementary Fig. 1c**. FMO for Notch2 served as an additional control for staining and gating. (**g, h**) Bar graphs showing relative frequency of Notch2^{+} cells (calculated within the depicted population) and Mean Fluorescence Intensity (MFI) of Notch2 in 1- and 10wk old Ctrl and *N2*$^{\Delta Cx3cr1}$ splenic Ly6C^{hi} monocytes and (**h**) RPM populations. (1wk: n = 7/9, 10wk: n = 5/6). (**i**) Bar graphs showing *Notch2* expression in splenic RPM, Ly6C^{hi} and Ly6C^{lo} monocytes sorted from Ctrl or *N2*$^{\Delta Cx3cr1}$ mice and analyzed by qRT-PCR. (RPM, Ly6C^{hi}: n = 6/6, Ly6C^{lo}: n = 5/6). (**g-i**) Data are mean ± SEM pooled from three experiments. *P<0.05; **P<0.01; ***P<0.001; (unpaired two-tailed Student's *t*-test). Source data are provided as a Source Data file.

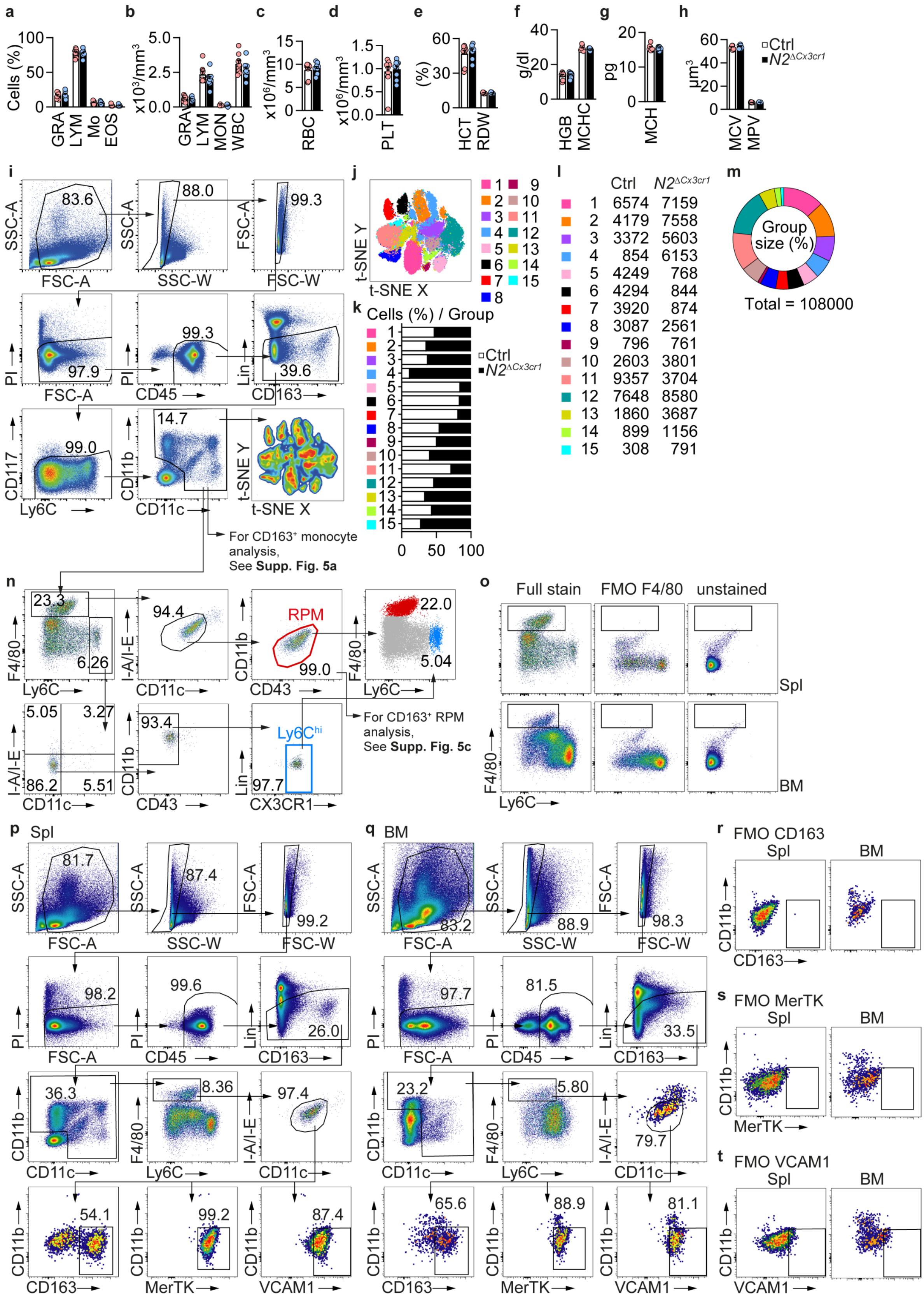

Supplementary Fig. 2. Gating strategies and general characterization of MPS. (**a-h**) Complete blood counts from 10 wk old Ctrl or *N2*$^{\Delta Cx3cr1}$ mice (n = 9/9). (**i**) Gating strategy for t-SNE and clustering analysis (PhenoGraph), and for definition of splenic phagocytic cells. Doublets, dead (PI^{+}), CD45^{neg}, Lin^{+}, CD117^{+} and CD11b/CD11c-double-negative cells were

excluded from Ctrl or *N2*$^{\Delta Cx3cr1}$ spleen samples prior to down-sampling, concatenation, t-SNE and PhenoGraph analysis. (**j**) t-SNE map with identified populations concatenated from Ctrl and *N2*$^{\Delta Cx3cr1}$ mice (n = 3/3, 108000 cells). (**k**) Ratio of cells in each population. (**l**) Absolute number of Ctrl and *N2*$^{\Delta Cx3cr1}$ cells across identified cell populations. (**m**) Donut chart showing relative group sizes of individual populations from total concatentated cells. (**n**) Gating strategy and (**o**) corresponding fluorescence minus one (FMO) controls for definition of splenic RPM and Ly6C^{hi} monocytes for conventional flow cytometry analysis. The same gating strategy was applied for definition and analysis of BMM and BM Ly6C^{hi} monocytes. (**p, q**) Coexpression of classical macrophage markers MerTK, VCAM1 and CD163 in F4/80^{hi} populations in the (**p**) Spl and (**q**) BM. (**r-t**) Corresponding FMOs. (**a-h**) Data are mean ± SEM pooled from three experiments. *P<0.05; **P<0.01; ***P<0.001; (unpaired two-tailed Student's *t*-test). Source data are provided as a Source Data file.

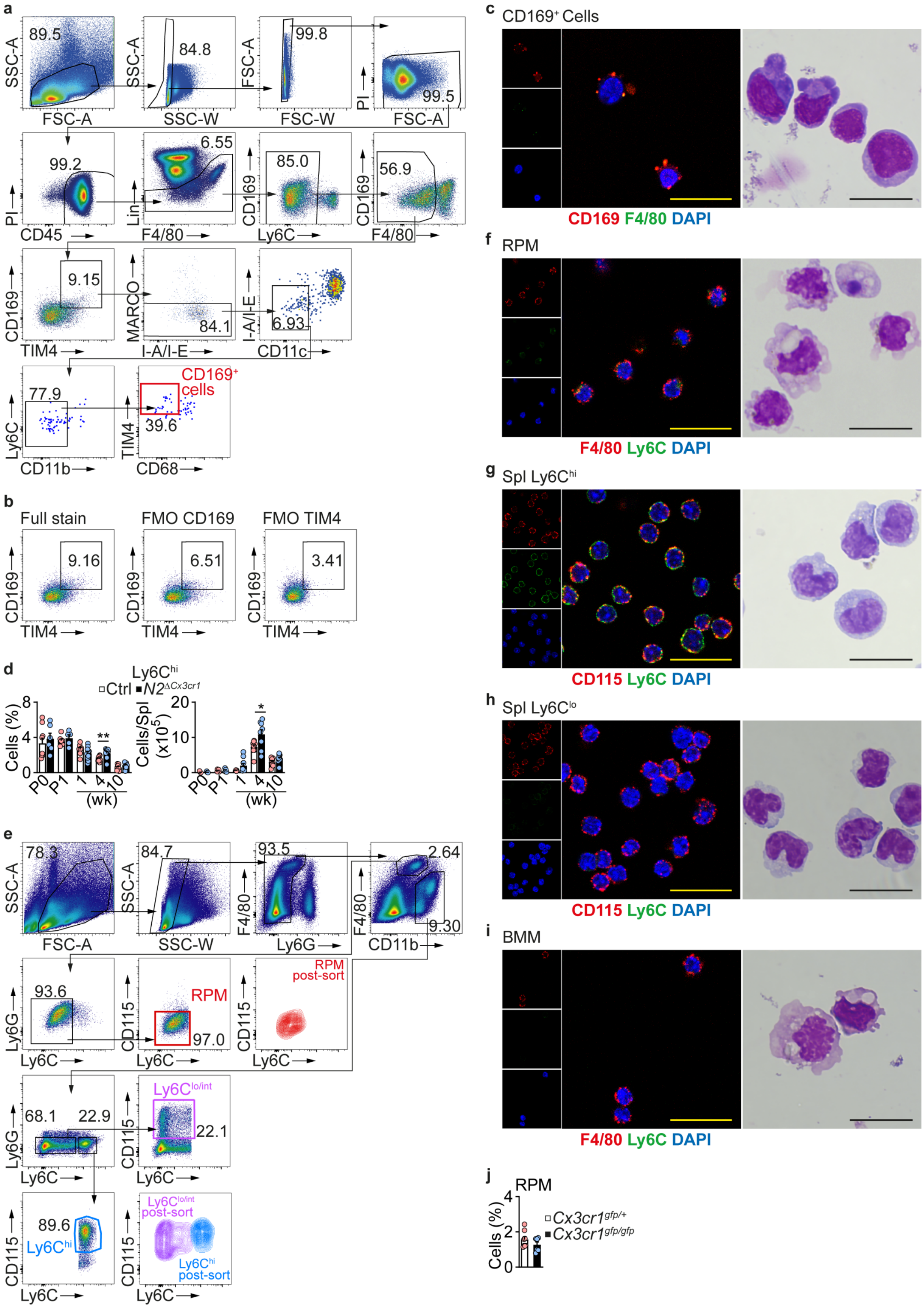

Supplementary Fig. 3. Strategies for identification, purification and morphological validation of Spl and BM cell populations. (**a**) Gating strategy and (**b**) corresponding FMO controls for definition of CD169^{+} cells by conventional flow cytometry. (**c**) CLSM and cytospin images of Spl CD169^{+} cells. (**d**) Relative (from live cell gate) and absolute numbers of splenic Ly6C^{hi} monocytes. Pooled from three experiments. For exact n numbers see Source Data.

(**e**) Sorting strategy and (**f-h**) corresponding CLSM and cytospin images of Spl RPM ($Lin^{lo/-}Ly6G^{lo/-}CD11b^{lo/-}Ly6C^{lo/-}F4/80^{hi}CD115^{neg}$), and $Ly6C^{hi}$ ($Lin^{lo/-}Ly6G^{lo/-}CD11b^{+}Ly6C^{hi}F4/80^{lo/-}CD115^{+}$) and $Ly6C^{lo}$ ($Lin^{lo/-}Ly6G^{lo/-}CD11b^{+}Ly6C^{lo/-}F4/80^{lo/-}CD115^{+}$) monocytes sorted for gene expression analysis. RPM and monocytes were sorted from Lin (CD3, CD19, B220, NK1.1) depleted splenic cells after exclusion of doublets and $Ly6G^{+}$ granulocytes. Related to **Figure 1m-p**. (**i**) CLSM and cytospin images of sorted BMM cells. (**j**) Bar graphs showing relative frequency (from live cell gate) of RPM in *Cx3cr1*$^{gfp/+}$ and *Cx3cr1*$^{gfp/gfp}$ mice (n = 8/6) Pooled from three experiments. (**c, f-i**) Images are representative of three experiments. Scale bar 20 µm. (**d, j**) Data are mean ± SEM. *P<0.05; **P<0.01; ***P<0.001; (unpaired two-tailed Student's *t*-test). Source data are provided as a Source Data file.

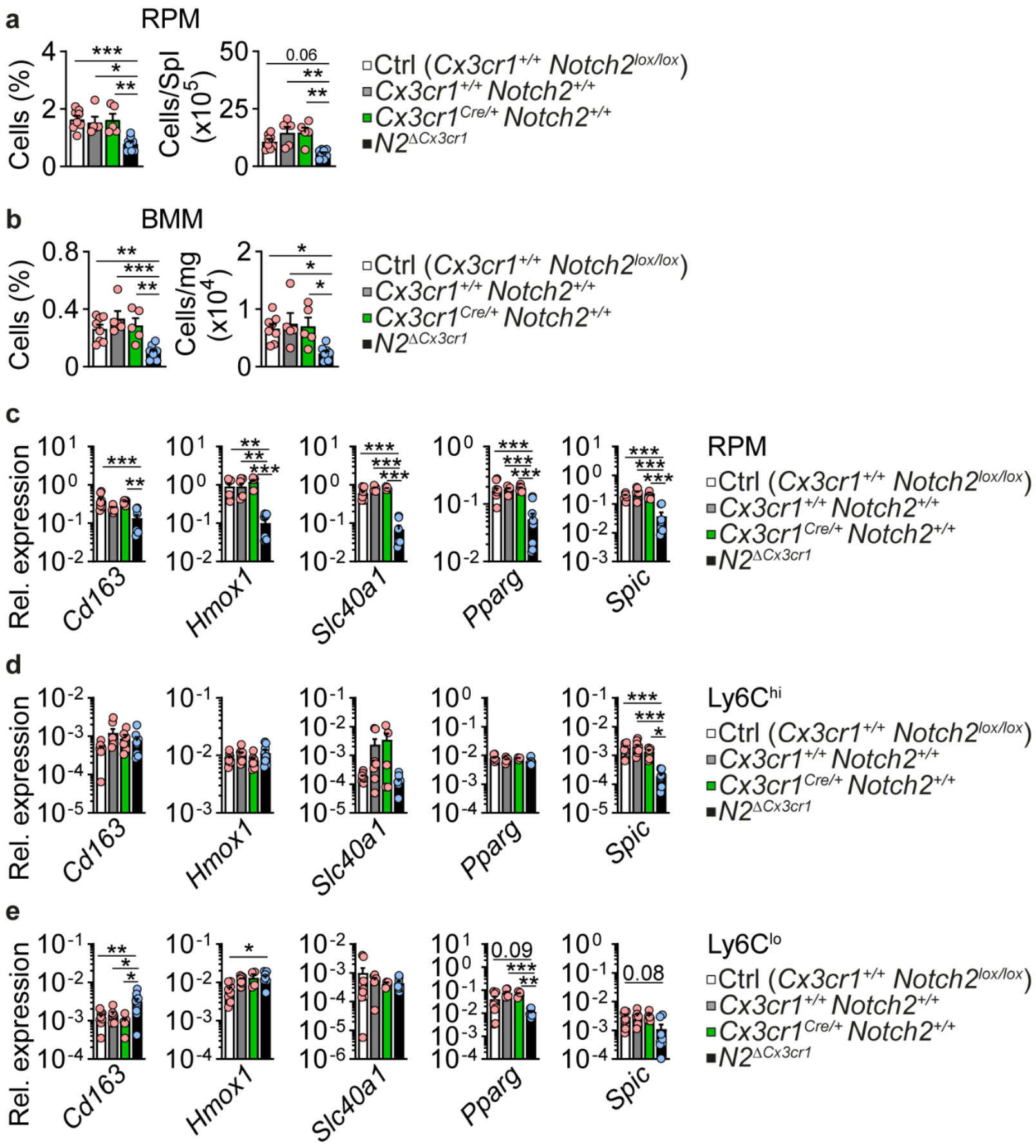

Supplementary Fig. 4. Control comparison excluding Cre-mediated off target effects. (**a,b**) Bar graphs showing relative (from live cell gate, left panel) or absolute frequency (right panel, per Spl or per mg BM) of (**a**) RPM and (**b**) BMM in Ctrl (Cre negative floxed mice; *Cx3cr1*$^{+/+}$ *Notch2*$^{lox/lox}$), Cre negative non-floxed (*Cx3cr1*$^{+/+}$ *Notch2*$^{+/+}$), Cre positive non-floxed (*Cx3cr1*$^{Cre/+}$ *Notch2*$^{+/+}$) and Cre positive floxed mice (*N2*$^{\Delta Cx3cr1}$) mice (n = 8/5/5/8). (**c-e**) Expression of signature genes in RPM and splenic monocytes. Ctrl and *N2*$^{\Delta Cx3cr1}$ groups include the samples shown in the main figure (**Fig. 1n-p**) plus additional data points. Exact n numbers are provided in the Source Data. **(a-e)** Data are mean ± SEM pooled from three experiments. *P<0.05; **P<0.01; ***P<0.001 (One-way ANOVA with Bonferroni's multiple comparison test). Source data are provided as a Source Data file.

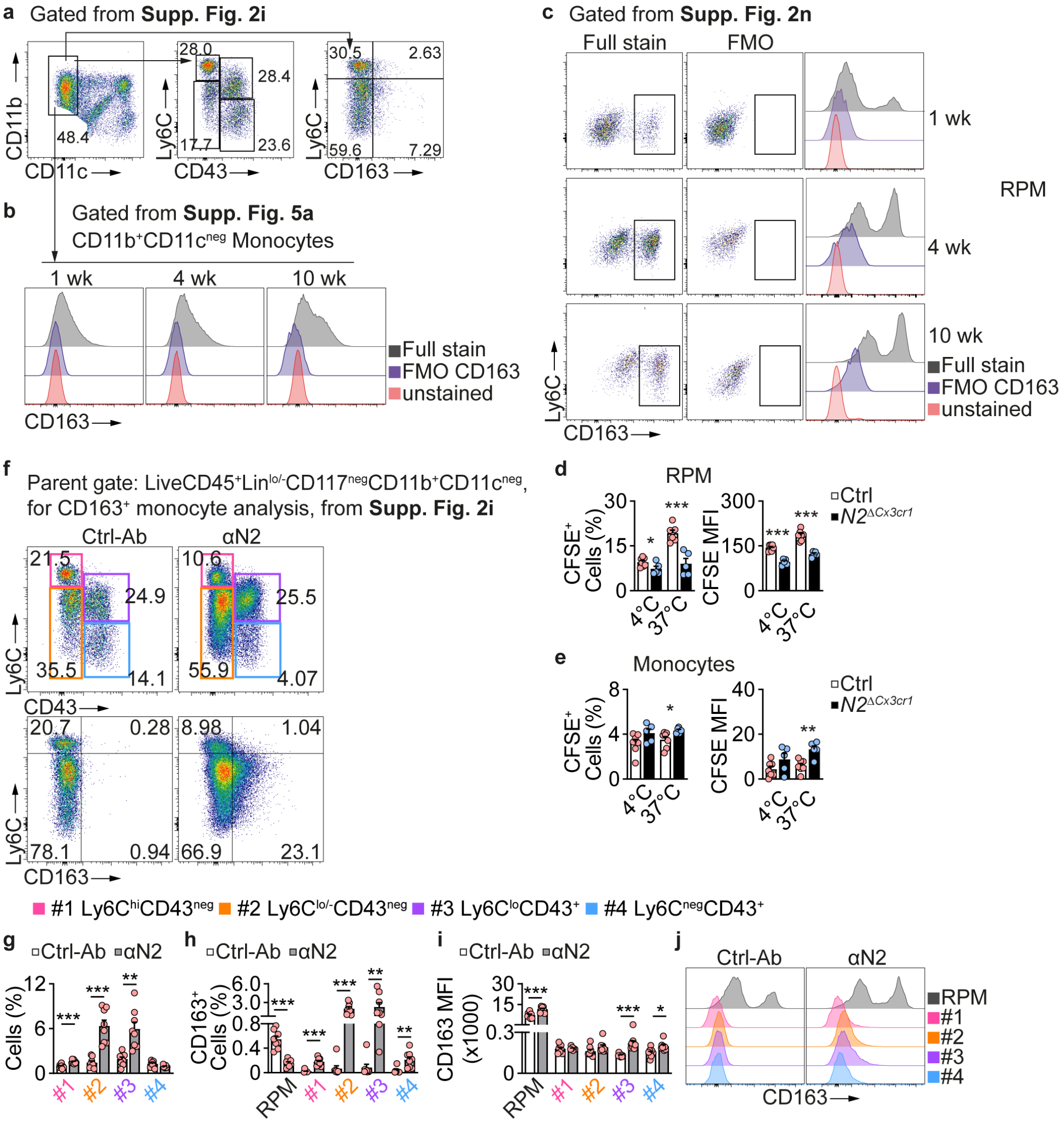

Supplementary Fig. 5. Gating strategy for identification of CD163^{+} population. (**a-c**) Gating strategy (**a**) and corresponding FMO controls (**b, c**) for definition of CD163^{+} monocytes and CD163^{+} RPM by conventional flow cytometry. (**d**) Relative frequency (from live cell gate) of CFSE^{+} RPM cells (left panel) and MFI of CFSE (right panel) within the RPM population *in vitro* in Ctrl or *N2$^{\Delta Cx3cr1}$* mice. RPM were defined as in **Supplementary Fig. 2n.** (**e**) Relative frequency (from live cell gate) of CFSE^{+} Monocytes (left panel) and MFI of CFSE (right panel) within the monocyte population *in vitro* in Ctrl or *N2$^{\Delta Cx3cr1}$* mice. Monocytes were defined as **Supplementary Fig. 5a**. (**d, e**) n = 7/5. (**f-j**) Analysis of monocyte subpopulations in wt mice treated with Ctrl-Ab or αN2-Ab. (**f**) Representative flow cytometry plots showing Spl monocyte populations (#1-4, upper panel), and expression of CD163 in all monocytes (lower panel). Values depict frequency of parent population. (**g**) Relative frequency (from live cell gate) of monocyte populations. (**h**) Relative frequency (from live cell gate) of CD163^{+} cells within RPM and monocyte populations. (**i, j**) MFI of CD163 and (**j**) representative histograms showing CD163 expression. (**g-i**) n = 8/8 (**d, e, g-i**) Data are mean ± SEM and pooled from three experiments. *P<0.05; **P<0.01; ***P<0.001; (unpaired two-tailed Student's t-test). Source data are provided as a Source Data file.

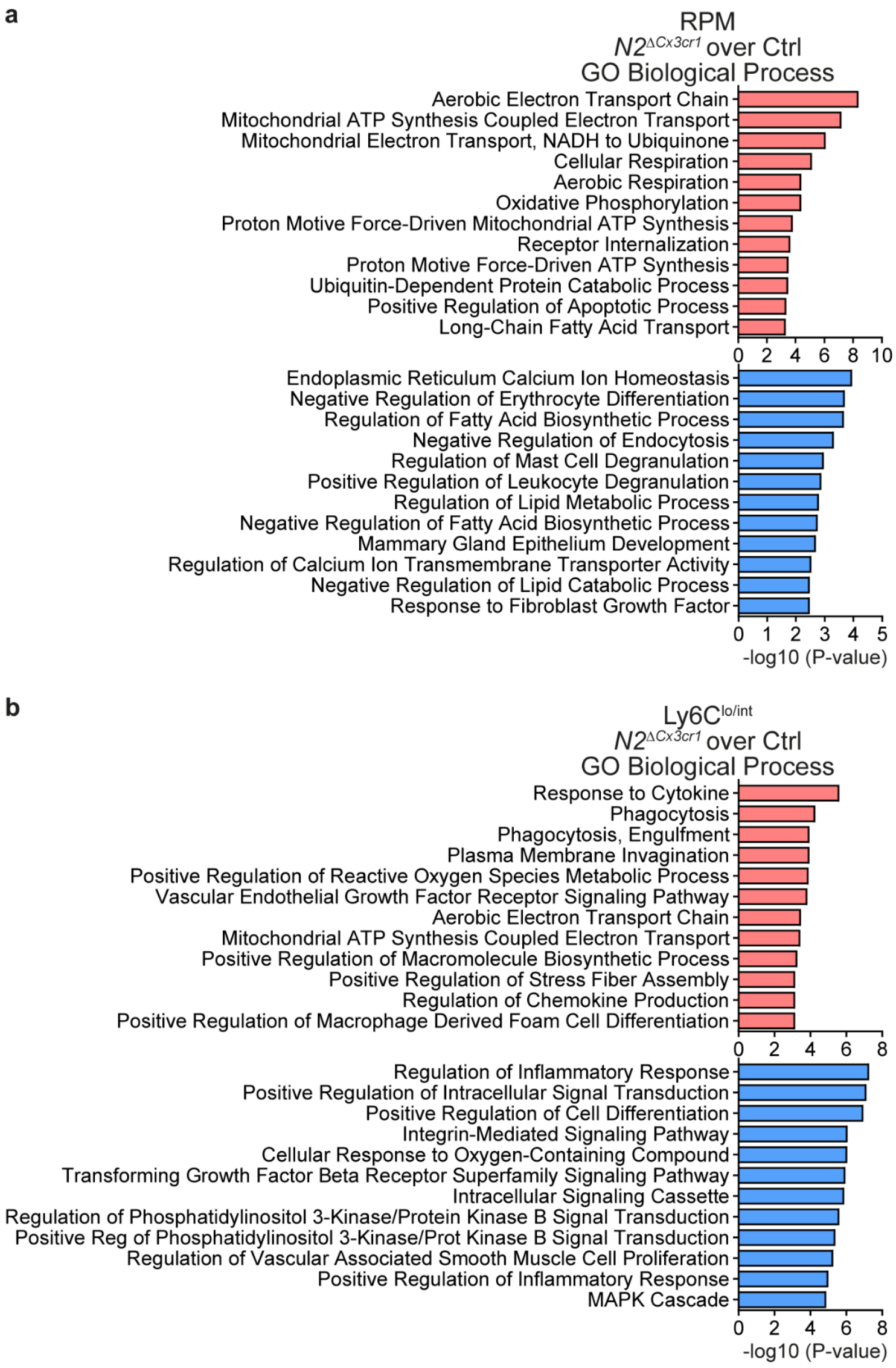

Supplementary Fig. 6. Gene Ontology (GO) enrichment analysis in ***N2$^{\Delta Cx3cr1}$*** **mice.** (**a, b**) Bar chart of the top 12 enriched terms from the GO Biological Process 2025 gene set library**.** Upregulated (red) and downregulated (blue) GO terms for Biological Process (BP) in *N2$^{\Delta Cx3cr1}$* (**a**) RPM or (**b**) Ly6C$^{lo/int}$ compared to Ctrl. (P < 0.05).

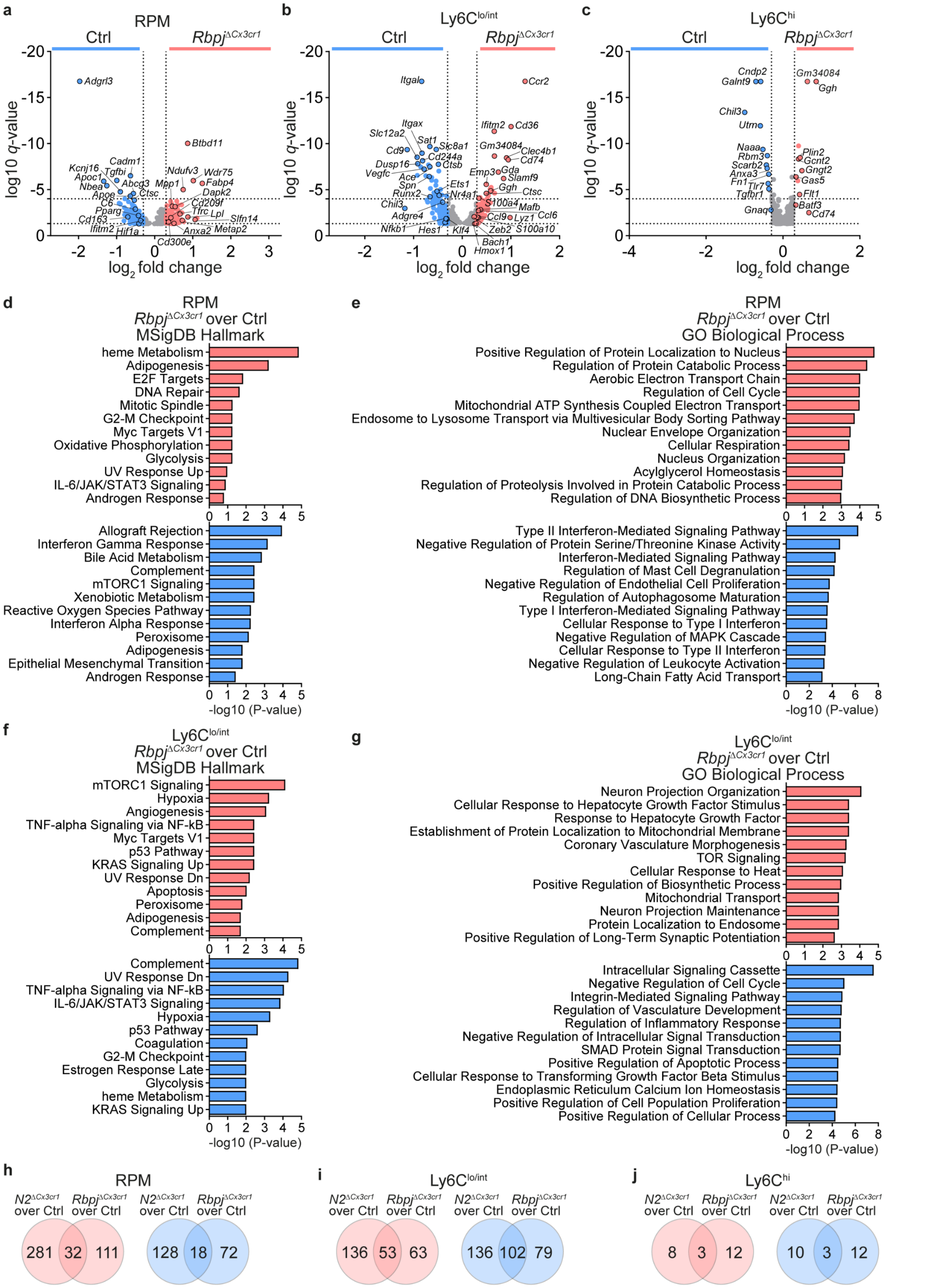

Supplementary Fig. 7. *Rbpj* loss is associated with broad alterations in transcriptional programs across RPM and Ly6C$^{lo/int}$ monocytes. (**a-c**) Volcano plots showing DEGs in splenic (**a**) RPM, (**b**) Ly6C$^{lo/int}$, and (**c**) Ly6C^{hi} monocytes between Ctrl and *Rbpj*$^{\Delta Cx3cr1}$ mice. Vertical dash lines indicate $\log_2$ fold change = 0.3 or -0.3. Horizontal lines indicate *q*-value =

0.05 or 0.0001. (**d, e**) Bar chart of top 12 enriched (**d**) Hallmark pathways from MSigDB or enriched terms from the (**e**) GO Biological Process 2025 gene set library for upregulated (red) or downregulated (blue) genes in *Rbpj*$^{\Delta Cx3cr1}$ RPM compared to Ctrl. ($P < 0.05$). (**f, g**) Bar chart of top 12 enriched (**f**) Hallmark pathways from MSigDB or enriched terms from the (**g**) GO Biological Process 2025 gene set library for upregulated (red) or downregulated (blue) genes in *Rbpj*$^{\Delta Cx3cr1}$ Ly6C$^{lo/int}$ monocytes compared to Ctrl. ($P < 0.05$). (**h-j**) Venn diagrams showing overlap of significantly upregulated (red) and downregulated (blue) genes in (**h**) RPM, (**i**) Ly6C$^{lo/int}$, or (**j**) Ly6C^{hi} monocytes between *N2*$^{\Delta Cx3cr1}$ and *Rbpj*$^{\Delta Cx3cr1}$ compared to Ctrl. ($P < 0.05$, $\log_2$ fold change ≥ 0.3 or ≤ -0.3).

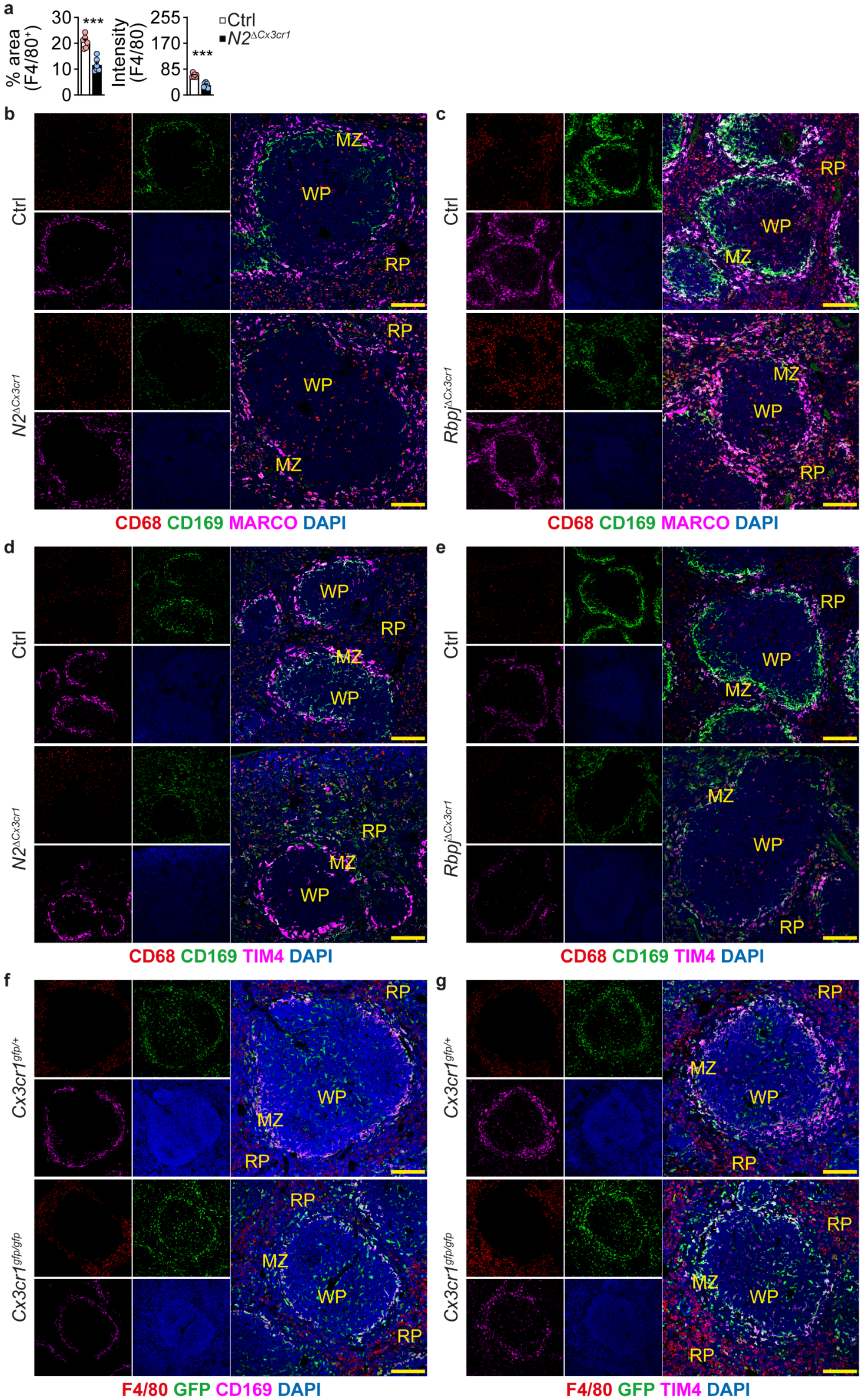

Supplementary Fig. 8. Impaired splenic microarchitecture and iron homeostasis in canonical *Notch2* signaling-deficient mice. (**a**) *In situ* quantification of F4/80$^+$ area and F4/80 signal intensity (n = 6/6). Data are mean ± SEM pooled from four experiments. *P<0.05; **P<0.01; ***P<0.001; (unpaired two-tailed Student's *t*-test). Source data are

provided as a Source Data file. (**b-e**) CLSM images of Spl from 10 wk old (**b, d**) Ctrl or *N2*$^{\Delta Cx3cr1}$ or (**c, e**) Ctrl or *Rbpj*$^{\Delta Cx3cr1}$ mice. (**f, g**) CLSM images of Spl from 10 wk old *Cx3cr1*$^{gfp/+}$ or *Cx3cr1*$^{gfp/gfp}$ mice. (**b-g**) Data are representative of (**b-e**) three or (**f, g**) two experiments. Scale bar 100 µm.

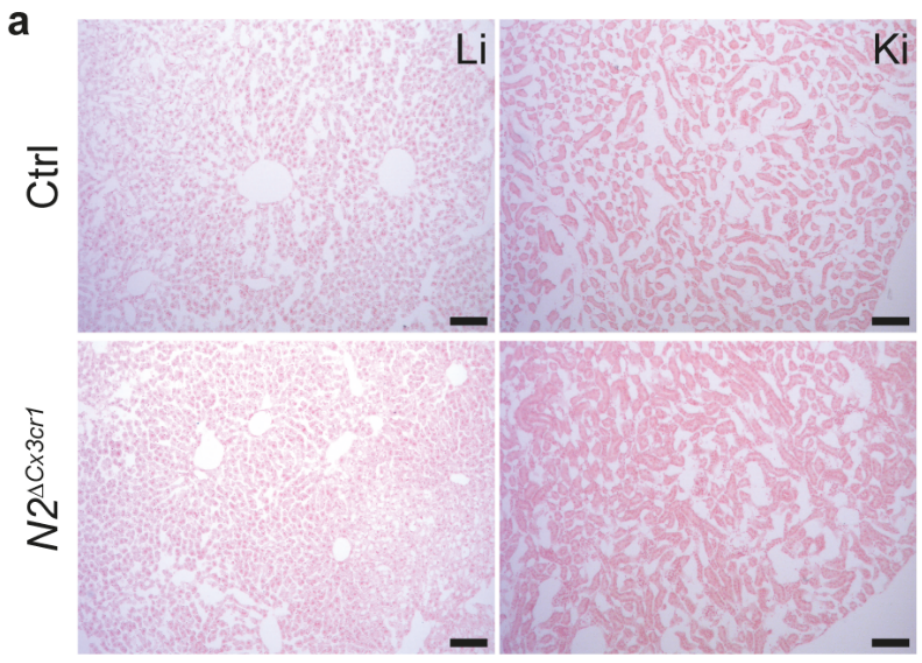

Supplementary Fig. 9. Absence of iron deposits in the Liver and Kidney. Perl's Prussian blue staining of Liver (Li) and Kidney (Ki) of 10 wk old Ctrl or *N2*$^{\Delta Cx3cr1}$ mice. Representative microscopy images from three experiments. Scale bar 100 µm.

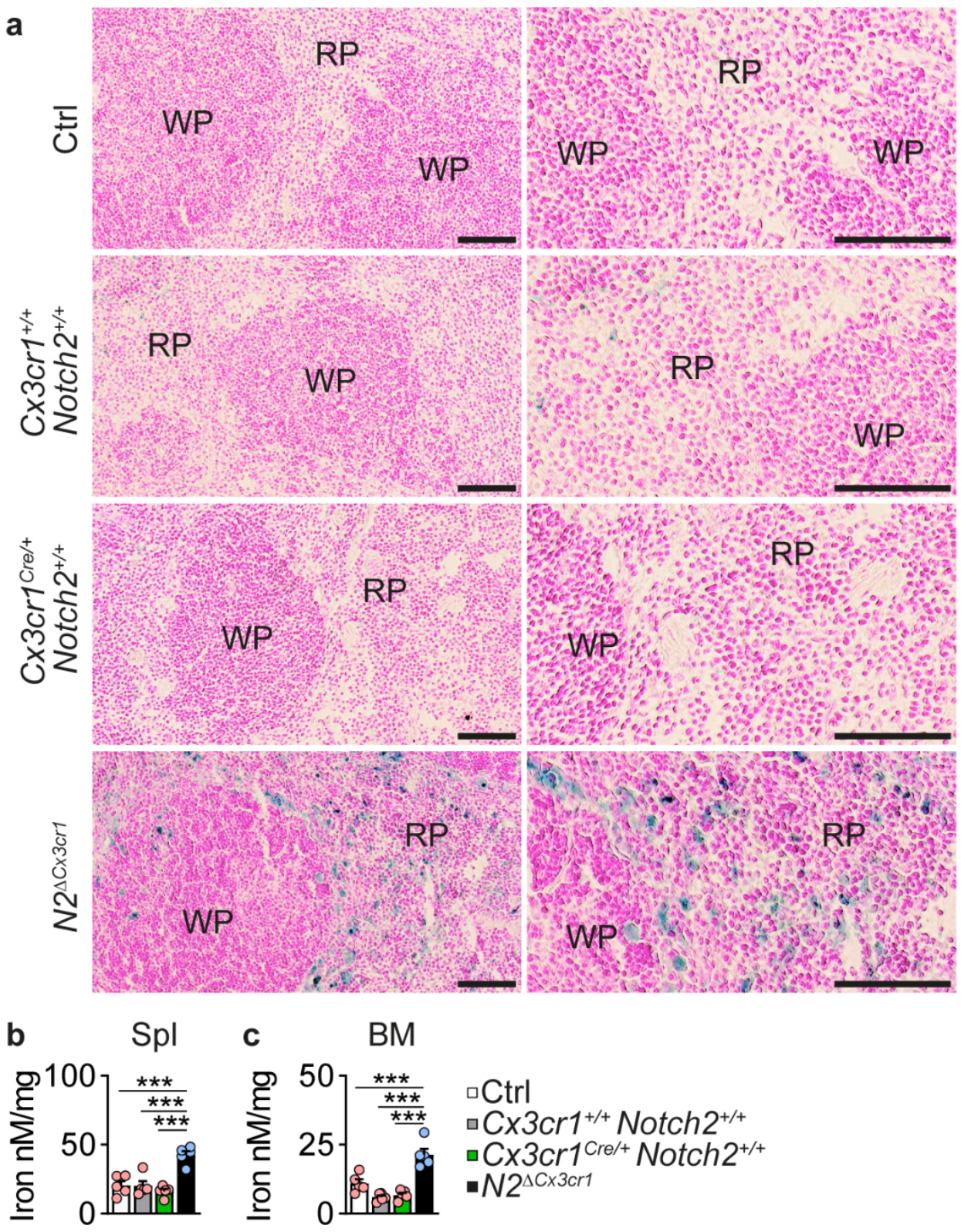

Supplementary Fig. 10. Prussian blue staining and iron quantification comparing control groups. (**a**) Perl's Prussian blue staining of Spl of 10 wk old Ctrl (Cre negative floxed mice; *Cx3cr1*$^{+/+}$ *Notch2*$^{lox/lox}$), Cre negative non-floxed (*Cx3cr1*$^{+/+}$ *Notch2*$^{+/+}$), Cre positive non-floxed (*Cx3cr1*$^{Cre/+}$ *Notch2*$^{+/+}$) and Cre positive floxed mice (*N2*$^{\Delta Cx3cr1}$) mice. Representative microscopy images from three experiments. Scale bar 100 µm. (**b, c**) Iron quantification in (**b**) Spl and (**c**) BM. Data are mean ± SEM. (Spl, BM: n = 5/5/5/5). Data are mean ± SEM pooled from three experiments. *P<0.05; **P<0.01; ***P<0.001 One-way ANOVA with Bonferroni's multiple comparison test. Source data are provided as a Source Data file.

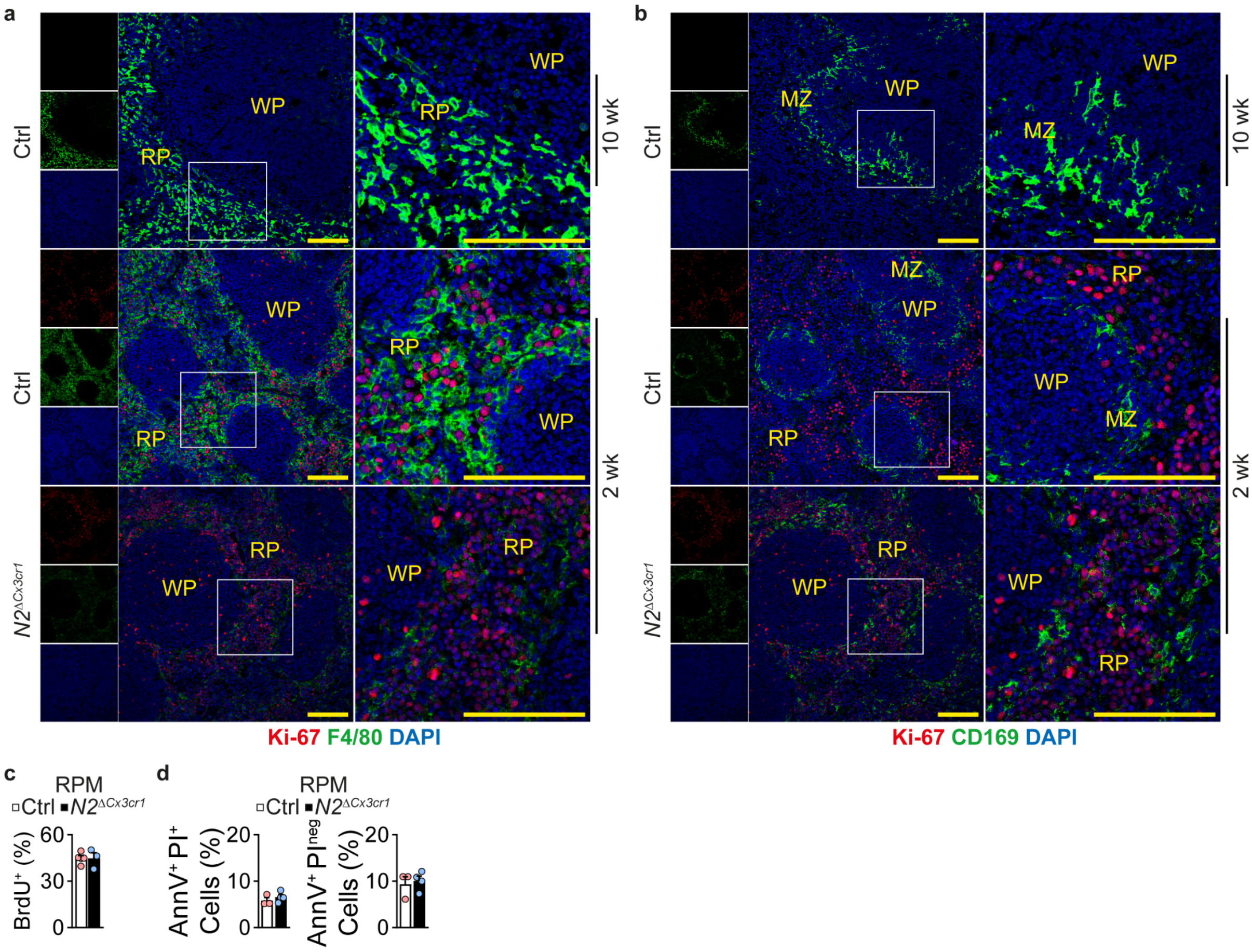

Supplementary Fig. 11. RPM Proliferation and Apoptosis. (**a, b**) CLSM images of Spl from 10 wk- or 2 wk-old Ctrl or $N2^{\Delta Cx3cr1}$ mice. Images are derived from the same sample but depicted separately for simplicity. Representative of two experiments. Scale bar 100 µm. (**c**) Frequency of $BrdU^{+}$ RPM in 2 wk old Spl (n = 5/3). (**d**) Frequency of $AnnV^{+}PI^{+}$ or $AnnV^{+}$ PI^{neg} RPM in 10 wk old mice (n = 3/4). (**c, d**) Data are mean ± SEM and pooled from two experiments. *P<0.05; **P<0.01; ***P<0.001; (unpaired two-tailed Student's *t*-test). Source data are provided as a Source Data file.

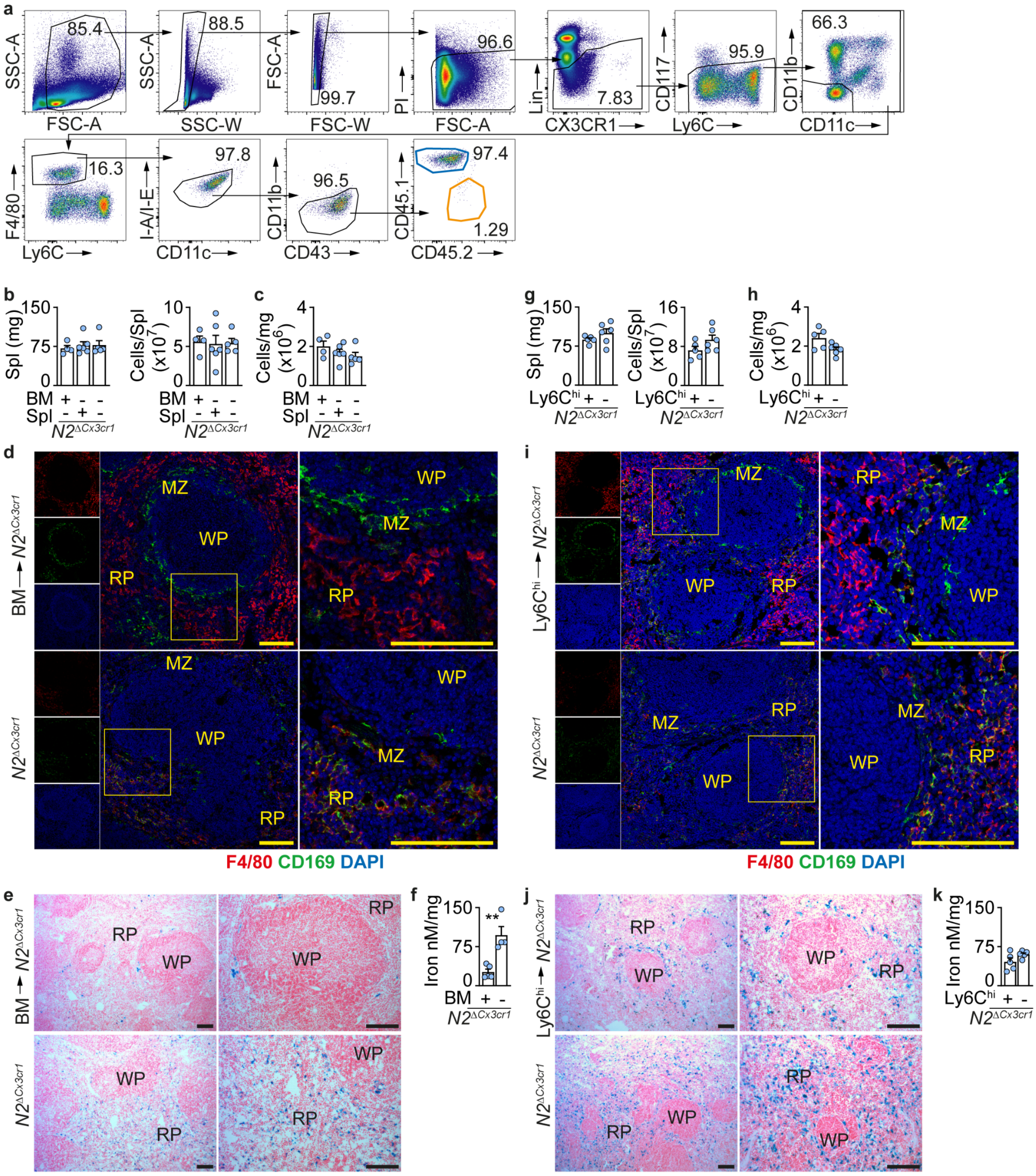

Supplementary Fig. 12. Reconstitution of the splenic niche with BM monocyte-derived RPM. (**a**) Complete gating strategy for identification of congenic CD45.1^{+} donor cells in recipient $N2^{\Delta Cx3cr1}$ mice. (**b, c**) Spl weight and total cell number (**b**) or frequency of BM cells normalized per mg BM (**c**) in recipient mice reconstituted with BM or Spl cells. (**d**) CLSM images of Spl from BM-reconstituted or non-reconstituted recipient $N2^{\Delta Cx3cr1}$ mice. (**e**) Perl's Prussian blue staining showing iron deposits in Spl of BM-reconstituted or non-reconstituted recipient mice. Representative of two experiments. Scale bar 100 µm. (**f**) Iron quantification in Spl of BM-reconstituted or non-reconstituted $N2^{\Delta Cx3cr1}$ mice (n = 5/4). (**g, h**) Spl weight and total cell number (**g**) or frequency of BM cells normalized per mg BM (**h**) in recipient $N2^{\Delta Cx3cr1}$ mice after reconstitution with BM Ly6C^{hi} monocytes. (**i**) CLSM images of Spl from BM Ly6C^{hi} monocyte-reconstituted or non-reconstituted recipient $N2^{\Delta Cx3cr1}$ mice. (**j**) Perl's Prussian blue staining showing iron deposits in recipient Spl of BM Ly6C^{hi} monocyte-reconstituted or non-reconstituted mice. Representative of three experiments. Scale bar 100 µm. (**k**) Iron quantification in Spl of BM Ly6C^{hi} monocyte-reconstituted or non-reconstituted $N2^{\Delta Cx3cr1}$ mice

(n = 5/6). (**b, c, f-h, k)** Data are mean ± SEM and pooled from two (**b, c, f**) or three (**g, h, k**) experiments. (**b, c**) n = 4/6/5. *P<0.05; **P<0.01; ***P<0.001; (One-way ANOVA with Bonferroni's multiple comparison test). (**g, h**) n = 5/6. (**f-h, k**) *P<0.05; **P<0.01; ***P<0.001; (unpaired two-tailed Student's *t*-test). (**d, i**) Images are derived from the same samples as in **Figure 5g, h (d)** and **Figure 5o, p** (**i**) respectively. Depicted as separate images for simplicity. Representative of two (**d**) or three (**i**) experiments. Scale bar 100 µm. Source data are provided as a Source Data file.

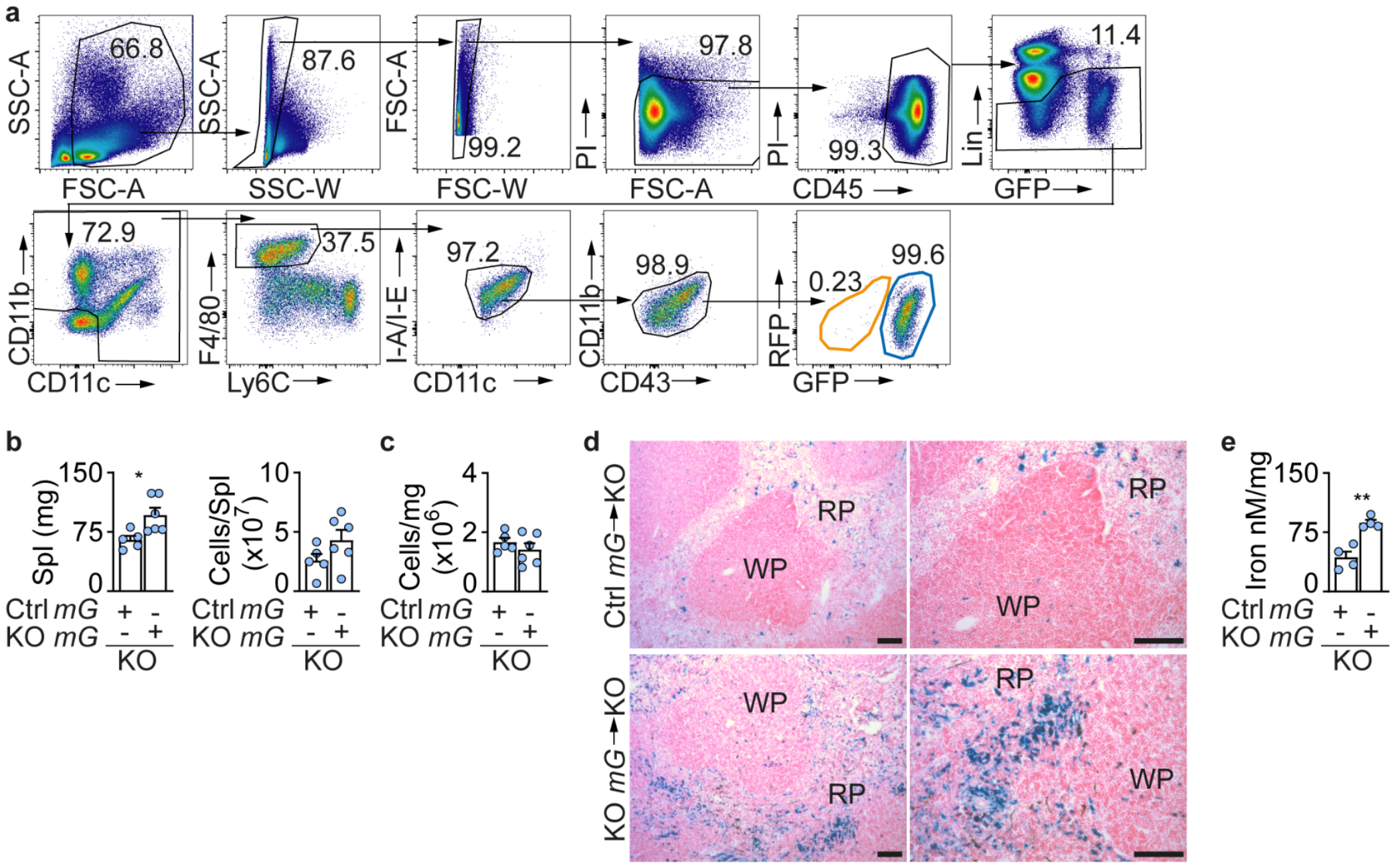

Supplementary Fig. 13. Development of RPM and BMM requires intrinsic Notch2 signaling. (**a**) Complete gating strategy for identification of donor cells in recipient *N2*$^{\Delta Cx3cr1}$ mice. (**b**) Spl weight and total cell number in recipient mice reconstituted with Ctrl *mG* or KO *mG* BM. (**c**) Frequency of BM cells normalized per mg BM in recipient mice after reconstitution with Ctrl *mG* or KO *mG* BM. (**d**) Perl's Prussian blue staining showing iron deposits in recipient Spl after reconstitution. Representative of three experiments. Scale bar 100 µm. (**e**) Iron quantification in recipient Spl after reconstitution (n = 4/4). (**b, c, e**) Data are mean ± SEM and pooled from three experiments. (**b, c**) n = 5/6. *P<0.05; **P<0.01; ***P<0.001; (unpaired two-tailed Student's *t*-test). Source data are provided as a Source Data file.

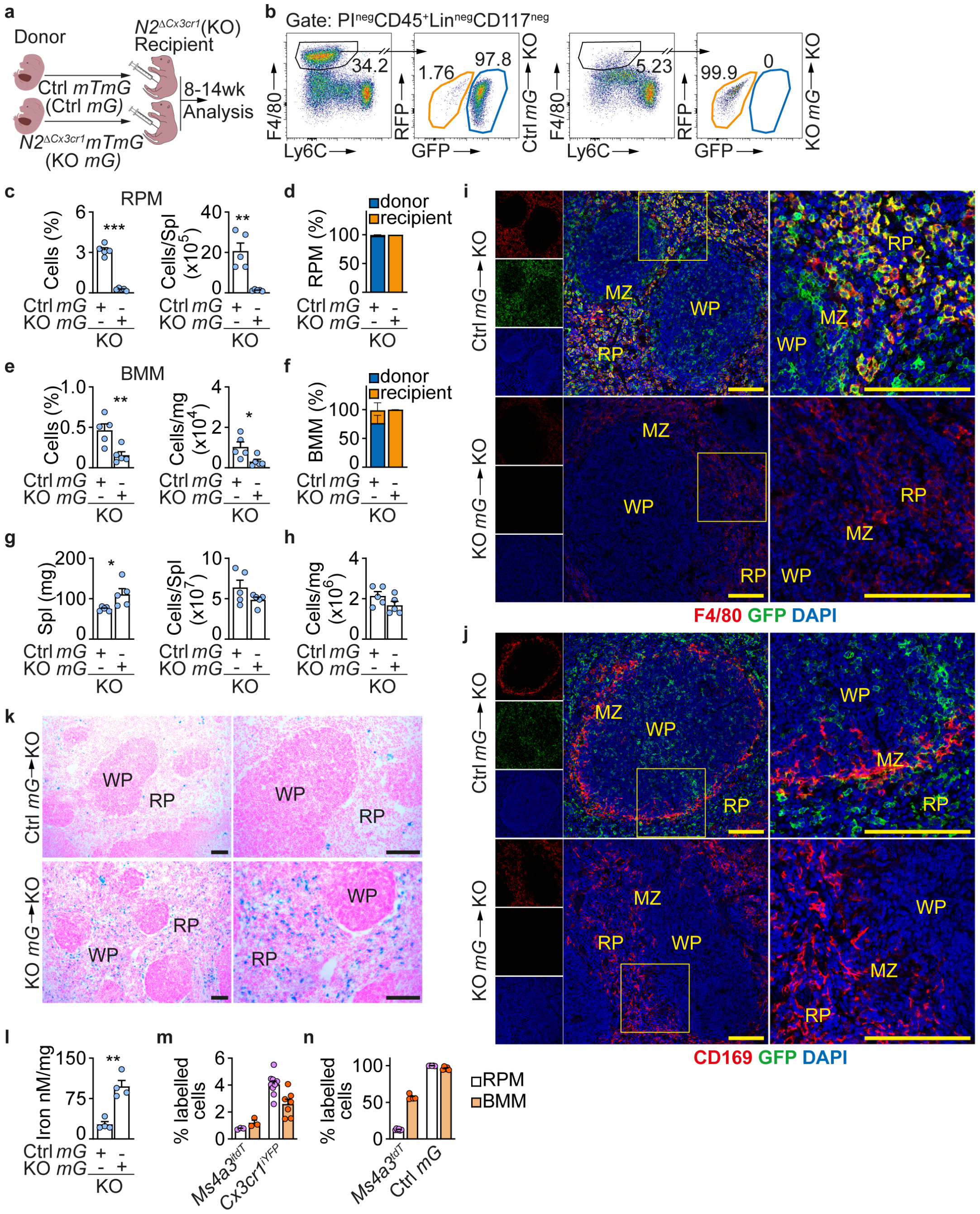

Supplementary Fig. 14. FL cells give rise to RPM in *Notch2*-dependent manner. (**a**) Experimental scheme of reconstitution study using FL cells. (**b**) Representative flow cytometry plot depicting donor FL-derived RPM in $N2^{\Delta Cx3cr1}$ recipients. Values depict frequency of parent population. Full gating strategy in **Supplementary Fig. 13a**. (**c**) Relative (from live cell gate) and absolute frequency of RPM in recipient mice. (**d**) Frequencies of donor- and recipient-derived RPM in whole RPM pool. (**e**) Relative (from live cell gate) and absolute frequency of BMM calculated per mg BM in recipient mice. (**f**) Frequencies of donor- and recipient-derived BMM in whole BMM pool. (**g**) Spl weight and total cell number in recipient mice reconstituted with Ctrl *mG* or KO *mG* FL cells. (**h**) Frequency of recipient BM cells normalized per mg BM after reconstitution with Ctrl *mG* or KO *mG* FL cells. (**i, j**) CLSM images of Spl from Ctrl *mG* or KO *mG* FL-reconstituted $N2^{\Delta Cx3cr1}$ mice. (**k**) Perl's Prussian blue staining showing iron deposits in recipient Spl after reconstitution with Ctrl *mG*- or KO *mG* FL cells. (**l**) Iron quantification in Spl of Ctrl *mG* or KO *mG* FL-reconstituted $N2^{\Delta Cx3cr1}$ mice

(n = 4/4, pooled from two experiments). (**m**) Frequency of labelled RPM (white) or BMM (orange) cells in adult *Ms4a3*^{itdT} (n = 3, pooled from two experiments) or *Cx3cr1*^{iYFP} animals (n = 9, pooled from three experiments). (**n**) Frequency of labelled RPM (white) or BMM (orange) cells in adult *Ms4a3*^{tdT} (n = 6, pooled from two experiments) or Ctrl *mG* animals (n = 5, pooled from two experiments). (**c-h**) n = 5/5, pooled from three experiments. (**c, e, g, h, l-n**) Data are mean ± SEM. *P<0.05; **P<0.01; ***P<0.001; (unpaired two-tailed Student's *t*-test). (**i-k**) Representative of (**i, j**) three or (**k**) two experiments. Scale bar 100 µm. Source data are provided as a Source Data file.

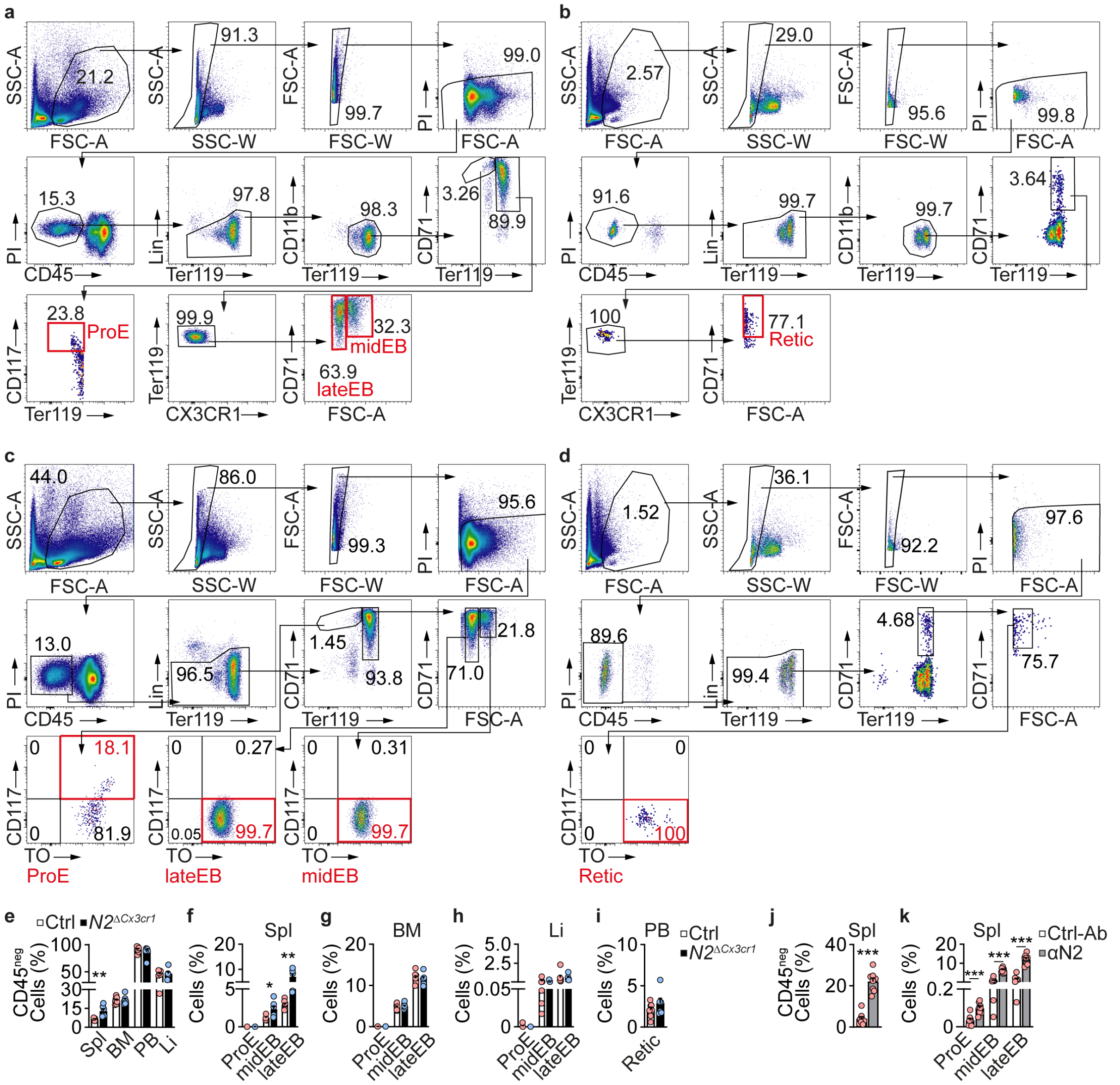

Supplementary Fig. 15. Gating strategy for identification of erythroid precursors. (**a, b**) Complete, primary gating strategy to identify (**a**) erythroid precursors (ProE, midEB, lateEB) and (**b**) reticulocytes in the PB (Retic). Lin (CD3, CD19, B220, Ly6G, CD41, NK1.1). (**c, d**) Alternative gating strategy with nuclear stain (TO, thiazole orange) to validate primary gating strategy. (**e-i**) Relative frequency of (**e**) CD45^{neg} cells, erythroid precursors (**f-h**) in the Spl, BM and Liver (Li), and reticulocytes (**i**) in PB using alternative gating strategy with nuclear stain. Spl, BM, Li, PB/Retic: n = 7/6, pooled from three experiments. (**j, k**) Relative frequency of CD45^{neg} cells (**j**) and erythroid precursors (**k**) in the Spl of wt mice treated with Ctrl-Ab or αN2-Ab. n = 8/8, pooled from three experiments. (**e-k**) Bar graphs depict mean ± SEM. *P<0.05; **P<0.01; ***P<0.001; (unpaired two-tailed Student's *t*-test). Source data are provided as a Source Data file.

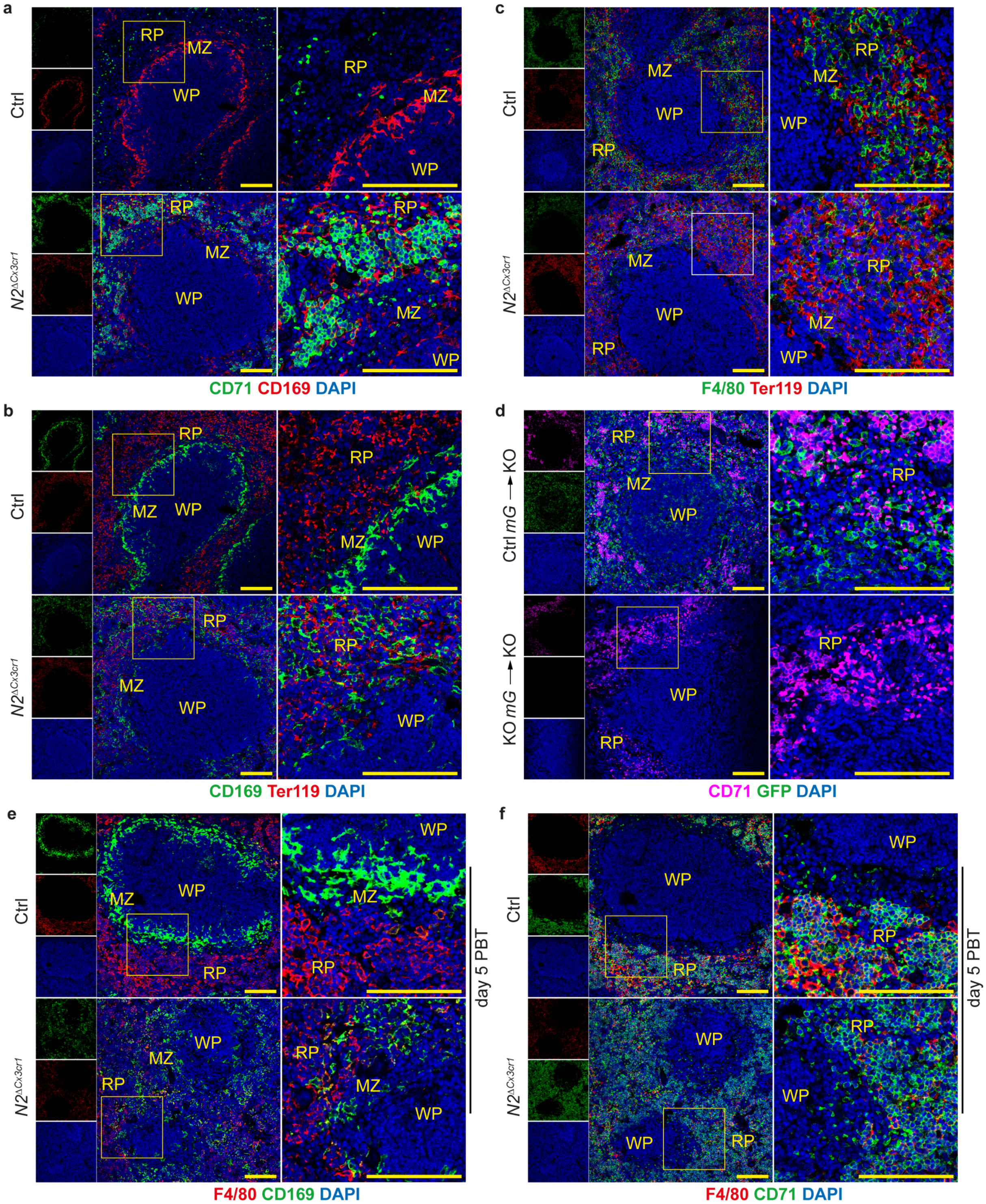

Supplementary Fig. 16. Enhanced extramedullary erythropoiesis in *N2*$^{\Delta Cx3cr1}$ mice (**a-c**) CLSM images of Spl from 10 wk old Ctrl or *N2*$^{\Delta Cx3cr1}$ mice. (**a, b**) Images are derived from the same sample but are shown separately for simplicity. (**c**) Derived from the same sample as in **Fig. 7i, j** but shown separately for simplicity. (**d**) CLSM images of Spl from 10 wk old *N2*$^{\Delta Cx3cr1}$ reconstituted with Ctrl *mG* or KO *mG* FL cells. (**e, f**) CLSM images of Spl from 10 wk old Ctrl or *N2*$^{\Delta Cx3cr1}$ mice 5 days post phlebotomy (PBT). (**a-f**) Representative of three experiments. Scale bar 100 µm.

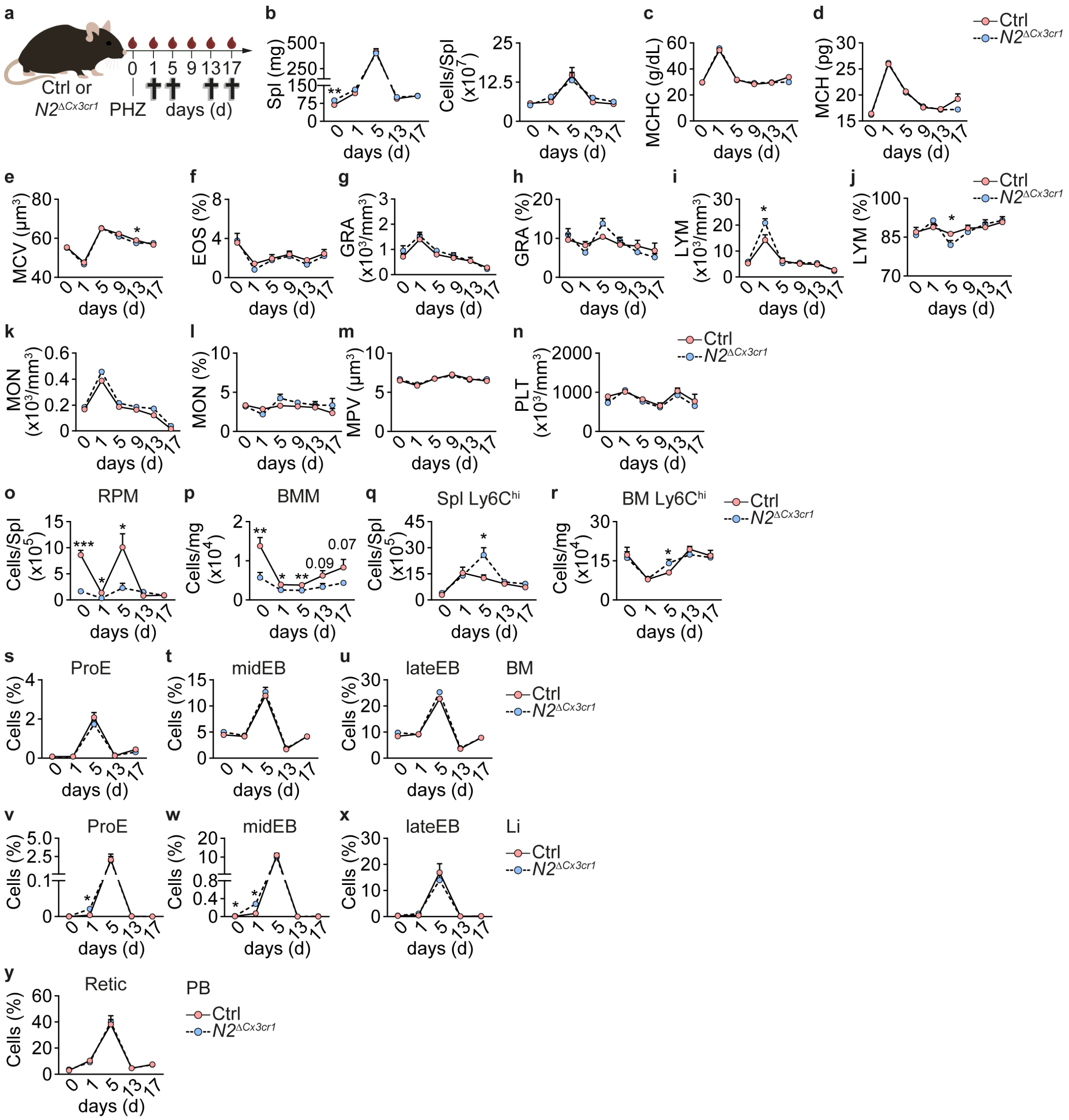

Supplementary Fig. 17. Notch2-deficiency is associated with an altered early response to PHZ-induced hemolytic anemia. (**a**) Experimental scheme. Blood samples were collected on indicated days, crosses represent experimental end points. (**b**) Spl weight and total cell number after induction of anemia. (**c-n**) Complete blood counts in Ctrl and *N2*$^{\Delta Cx3cr1}$ mice injected with PHZ. (**o-r**) Absolute frequency of (**o**) RPM, (**p**) BMM, (**q**) Spl Ly6C^{hi} monocytes and (**r**) BM Ly6C^{hi} monocytes in PHZ-treated mice. (**s-y**) Relative frequency (from live cell gate) of erythroid precursors in (**s-u**) BM or (**v-x**) Li, and reticulocytes in (**y**) PB of PHZ-treated mice. (**b-y**). Data are mean ± SEM pooled. *P<0.05; **P<0.01; ***P<0.001; (unpaired two-tailed Student's *t*-test). Each time point pooled from three experiments except (**c-n**) where 0d is pooled from five experiments. For exact n numbers see Source Data. Source data are provided as a Source Data file.

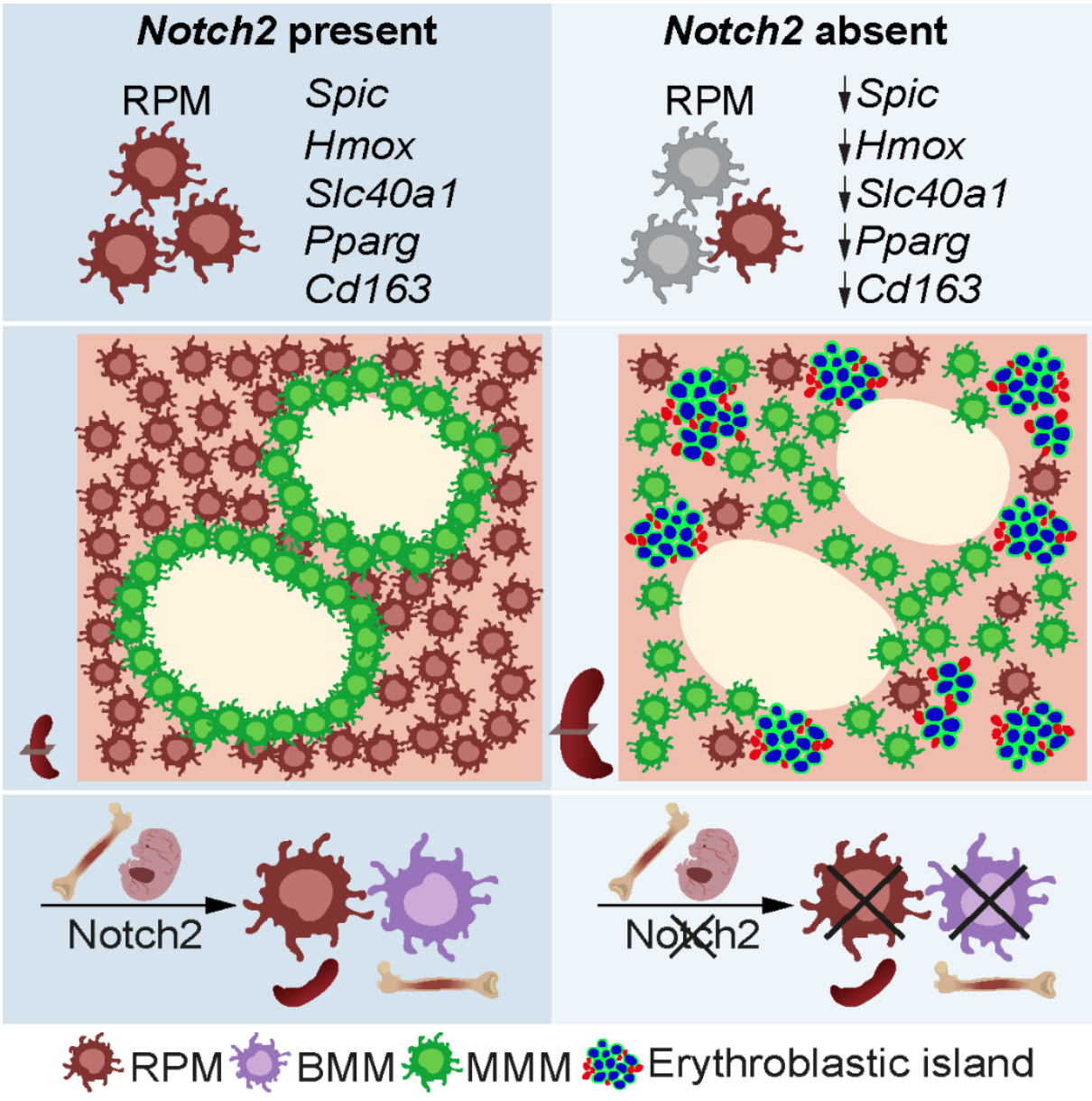

Supplementary Fig. 18. Role of Notch2 in Development and Maintenance of Iron-recycling Macrophages and Hematopoiesis. Loss of Notch2 leads to a marked reduction of RPM and BMM and downregulation of RPM signature genes, accompanied by splenomegaly, altered splenic architecture and enhanced extramedullary hematopoiesis. Postnatal transfer of bone marrow and fetal liver progenitors rescues RPM and BMM defects in a Notch2-dependent manner.

Supplementary Table 1: Surface phenotype signatures used for identification of distinct cell populations *in vivo*.

Population	Phenotype
RPM/BMM	Live(PI^{neg})CD45^{+}Lin$^{lo/-}$CD117^{neg}F4/80^{hi}Ly6C$^{lo/-}$I-A/I-E$^{lo/-}$CD11c$^{lo/-}$CD11b$^{lo/-}$ CD43$^{lo/-}$
CD169^{+} Cells	Live(PI^{neg})CD45^{+}Lin$^{lo/-}$Ly6C^{neg}F4/80^{lo}CD169^{+}MARCO^{neg}CD11b^{neg}CD11c^{neg} I-A/I-E$^{lo/-}$TIM4^{+}CD68^{neg}
Ly6C^{hi} monocytes	Live(PI^{neg})CD45^{+}Lin$^{lo/-}$CD117^{neg}F4/80$^{lo/-}$Ly6C^{hi}I-A/I-E^{neg}CD11c^{neg}CD11b^{+} CD43^{neg}CX3CR1^{+}
ProE	Live(PI^{neg})CD45^{neg}Lin$^{lo/-}$CD11b$^{lo/-}$CD71^{int}Ter119^{int}CD117^{+}
midEB	Live(PI^{neg})CD45^{neg}Lin$^{lo/-}$CD11b$^{lo/-}$CD71^{hi}Ter119^{hi}FSC-A^{hi}
lateEB/Retic	Live(PI^{neg})CD45^{neg}Lin$^{lo/-}$CD11b$^{lo/-}$CD71^{hi}Ter119^{hi}FSC-A^{lo}

For RPM/BMM, CD169^{+} cells, Ly6C^{hi} monocytes;

Lin: CD3, CD19, B220, Ly6G, Ter119, NK1.1

For ProE, earlyEB, lateEB, Retic;

Lin: CD3, CD19, B220, Ly6G, CD41, NK1.1

Supplementary Table 2: List of antibodies, reagents and software used in this study.

Antibody/Reagent	Dilution	Supplier	Identifier
Annexin V APC	1:200	Biolegend	Cat#640920; RRID: AB_2561515
Armenian Hamster Monoclonal Anti-mouse CD11c Brilliant Violet™ 605	1:400	Biolegend	Cat#117334; Clone N418; RRID: AB_2562415
Armenian Hamster Monoclonal Anti-mouse CD16.2 (FcγRIV)	1:200	Biolegend	Cat#149602; Clone 9E9; RRID: AB_2565302
Armenian Hamster Monoclonal Anti-mouse FcεRIα PE	1:200	Biolegend	Cat#134307; Clone MAR-1; RRID: AB_1626104
DAPI (4',6-Diamidino-2-phenylindol Dihydrochlorid)	0.2µg/ml	Carl Roth	Cat#6335.1
Goat Polyclonal Anti-Rabbit FITC	1:400	Jackson ImmunoResearch	Cat#111-095-144; RRID: AB_2337978
MojoSort™ Mouse anti-APC Nanobeads	1:20	Biolegend	Cat#480072
MojoSort™ Mouse anti-PE Nanobeads	1:10	Biolegend	Cat#480080
Monoclonal Anti-Mouse BrdU Alexa Fluor® 647	1:200	BD Biosciences	Cat#560209; Clone 3D4; RRID: AB_1645615
Mouse Monoclonal Anti-mouse CD45.1 APC	1:400	Biolegend	Cat#110713; Clone A20; RRID: AB_313502
Mouse Monoclonal Anti-mouse CD45.2 Alexa Fluor® 700	1:200	Biolegend	Cat#109821; Clone 104; RRID: AB_493730
Mouse Monoclonal Anti-mouse CX3CR1 Alexa Fluor® 488	1:200	Biolegend	Cat#149022; Clone SA011F11; RRID: AB_2565705
Mouse Monoclonal Anti-mouse CX3CR1 PE	1:200	Biolegend	Cat#149005; Clone SA011F11; RRID: AB_2564314
Mouse Monoclonal Anti-mouse NK1.1 Biotin	1:400	Biolegend	Cat#108703; Clone PK136; RRID: AB_313390
Mouse Monoclonal Anti-mouse NK1.1 PE	1:300	Biolegend	Cat#108707; Clone PK136; RRID: AB_313394
Propidium iodide (PI)	1 mg/ml (Stock) 1:12000	Sigma-Aldrich	Cat#81845; RRID:
Rabbit Polyclonal Anti-GFP	1:300	Acris/OriGene	Cat#SP3005P; RRID: AB_1002028
Rat Monoclonal Anti mouse CD43 PerCP-Cy5.5	1:400	BD Biosciences	Cat#562865; Clone S7; RRID: AB_2737851
Rat Monoclonal Anti-mouse CD115 (CSF-1R) Alexa Fluor® 488	1:100	Biolegend	Cat#135512; Clone AFS98; RRID: AB_11218983

Rat Monoclonal Anti-mouse CD115 (CSF-1R) PE	1:100	Biolegend	Cat#135505; Clone AFS98; RRID: AB_1937254
Rat Monoclonal Anti-mouse CD117 (c-kit) APC-Cy7	1:100	Biolegend	Cat#105825; Clone 2B8; RRID: AB_1626280
Rat Monoclonal Anti-mouse CD163 PE	1:200	Biolegend	Cat#155307; Clone S15049I; RRID: AB_2814061
Rat Monoclonal Anti-Mouse CD169 (Siglec-1) Alexa Fluor® 647	1:200	Biolegend	Cat#142407; Clone 3D6.112; RRID:AB_2563620
Rat Monoclonal Anti-mouse CD169 (Siglec-1) FITC	1:200	Biolegend	Cat#142405; Clone 3D6.112; RRID: AB_2563106
Rat Monoclonal Anti-mouse CD169 (Siglec-1) PE/Dazzle™ 594	1:200	Biolegend	Cat#142423; Clone 3D6.112; RRID: AB_2750058
Rat Monoclonal Anti-mouse CD19 Biotin	1:400	Biolegend	Cat#115503; Clone 6D5; RRID: AB_313638
Rat Monoclonal Anti-mouse CD19 PE	1:400	Biolegend	Cat#115507; Clone 6D5; RRID: AB_313642
Rat Monoclonal Anti-mouse CD3 Biotin	1:200	Biolegend	Cat#100243; Clone 17A2; RRID: AB_2563946
Rat Monoclonal Anti-mouse CD3 PE	1:200	Biolegend	Cat# 100205; Clone 17A2; RRID: AB_2563946
Rat Monoclonal Anti-Mouse CD41 PE/Dazzle™ 594	1:200	Biolegend	Cat#133935; Clone MWReg30; RRID: AB_2750529
Rat Monoclonal Anti-mouse CD45 Alexa Fluor® 700	1:200	Biolegend	Cat#103128; Clone 30-F1; RRID: AB_493715
Rat Monoclonal Anti-Mouse CD49b PE	1:300	BD Biosciences	Cat#553858; Clone DX5; RRID: AB_395094
Rat Monoclonal Anti-mouse CD68 APC/Cyanine7	1:100	Biolegend	Cat#137023; Clone FA-1; RRID: AB_2616812
Rat Monoclonal Anti-Mouse CD71 APC	1:800	Biolegend	Cat#113819; Clone RI7217; RRID: AB_2728134
Rat Monoclonal Anti-Mouse CD71 FITC	1:200	Biolegend	Cat#113805; Clone RI7217; RRID: AB_313566
Rat Monoclonal Anti-Mouse CD90.2 (Thy1.2) PE	1:800	Biolegend	Cat#140307; Clone 53-2.1; RRID: AB_10641145
Rat Monoclonal Anti-Mouse F4/80 Alexa Fluor® 594	1:200	Biolegend	Cat#123140; Clone BM8; RRID: AB_2563241
Rat Monoclonal Anti-Mouse F4/80 APC	1:400	Biolegend	Cat#123116; Clone BM8; RRID: AB_893481
Rat Monoclonal Anti-mouse F4/80 Brilliant Violet™ 650	1:200	Biolegend	Cat#123149; Clone BM8; RRID: AB_2564589
Rat Monoclonal Anti-Mouse F4/80 PE	1:200	Biolegend	Cat#123110; Clone BM8; RRID: AB_893486

Rat Monoclonal Anti-mouse I-A/I-E Brilliant Violet™510	1:400	Biolegend	Cat#107635; Clone M5/114.15.2; RRID: AB_2561397
Rat Monoclonal Anti-mouse Ly6C PE/Cyanine7	1:2800	Biolegend	Cat#128017; Clone HK1.4; RRID: AB_1732093
Rat Monoclonal Anti-mouse Ly6G Biotin	1:400	Biolegend	Cat#127603; Clone 1A8; RRID: AB_1186105
Rat Monoclonal Anti-mouse Ly6G FITC	1:400	Biolegend	Cat#127605; Clone 1A8; RRID: AB_1236488
Rat Monoclonal Anti-mouse Ly6G PE	1:800	Biolegend	Cat#127608; Clone 1A8; RRID: AB_1186099
Rat Monoclonal Anti-mouse MARCO	1:2	Bio-Techne	Cat# FAB2956F; Clone 579511; RRID: AB_2044695
Rat Monoclonal anti-mouse MerTK APC	1:100	Biolegend	Cat#151507; Clone 2B10C42; RRID: AB_2650738
Rat Monoclonal Anti-mouse Ter119 Biotin	1:400	Biolegend	Cat#116203; Clone Ter119; RRID: AB_313704
Rat Monoclonal Anti-mouse Ter119 Brilliant Violet™ 650	1:100	Biolegend	Cat# 116235; Clone Ter119; RRID: AB_11204244
Rat Monoclonal Anti-mouse Ter119 PE	1:200	Biolegend	Cat# 116208; Clone Ter119; RRID: AB_ 313709
Rat Monoclonal Anti-mouse Tim-4 Alexa Fluor® 647	1:200	Biolegend	Cat#130007; Clone RMT4-54; RRID: AB_2201842
Rat Monoclonal anti-mouse TruStain FcX™ (CD16/32)	1:200	Biolegend	Cat#101319; Clone 93; RRID: AB_1574973
Rat Monoclonal anti-mouse VCAM1 (CD106) PerCP-Cy5.5	1:100	Biolegend	Cat#105715; Clone 429; RRID: AB_1595594
Rat Monoclonal Anti-mouse/human B220 (CD45R) Biotin	1:400	Biolegend	Cat#103203; Clone RA3-6B2; RRID: AB_312988
Rat Monoclonal Anti-mouse/human B220 (CD45R) PE	1:600	Biolegend	Cat#103207; Clone RA3-6B2; RRID: AB_312992
Rat Monoclonal Anti-mouse/human CD11b Pacific Blue™	1:400	Biolegend	Cat#101223; Clone M1/70; RRID: AB_755985
Rat Monoclonal Anti-mouse/human Ki-67 Alexa Fluor® 647	1:200	Biolegend	Cat#151206; Clone 11F6; RRID: AB_2566801
Streptavidin APC/Cyanine7	1:800	Biolegend	Cat#405208
Streptavidin PE/Dazzle™ 594	1:400	Biolegend	Cat#405247

Thiazole Orange (TO)	10 µg/ml (Stock); 1:5 PB 1:100 Organs	Sigma-Aldrich	Cat#390062-250MG
Anti-Notch2 Antibody	20 mg/kg	Genentech	Non-commercial
Control Antibody	20 mg/kg	Genentech	Non-commercial
**Chemicals**			
4-(2-hydroxyethyl)-1-piperazineethanesulfonic acid (HEPES)		Sigma-Aldrich	Cat#H3375-25G
5-Bromo-2-deoxyuridine (BrdU)		Sigma-Aldrich	Cat#B5002-100MG
BD Cytoperm™ Permeabilization Buffer Plus		BD Biosciences	Cat#561651
Collagenase Type II		Worthington	Cat#LS004177
Deoxyribonuclease I (DNAse)		Sigma-Aldrich	Cat#D4527
Ferrozine		Sigma-Aldrich	Cat#160601
Fraction V, BSA Bovine Serum Albumin (BSA)		Roth	Cat#8076.2
Hemin		Sigma-Aldrich	Cat#51280-1G
Histopaque®-1083		Sigma-Aldrich	Cat#10831-100ML
Hydrochloric acid (HCl) solution 1.0N		Sigma-Aldrich	Cat#H9892
Iron (III) Chloride ($FeCl_3$)		Sigma-Aldrich	Cat#8.03945
L-(+)-Ascorbic Acid		Sigma-Aldrich	Cat#255564-5G
Phenylhydrazine-hydrochloride		Sigma-Aldrich	Cat#114715-5G
Red Blood Cell Lysis Buffer (10x)		Biolegend	Cat#420301
Sodium acetate anhydrous		Sigma-Aldrich	Cat#71183
Sodium chloride		Roth	Cat#3957.3
Sodium hydroxide		Sigma-Aldrich	Cat#1.09136
Sodium pyrophosphate tetrabasic		Sigma-Aldrich	Cat#P8010-500G
Trichloracetic acid (TCA)		Sigma-Aldrich	Cat#T6399-250G
Tamoxifen		Sigma-Aldrich	Cat#T5638
**Critical commercial assays**			
APC Annexin V Apoptosis Detection Kit with PI		Biolegend	Cat#640932
BD Cytofix/Cytoperm™ Kit		BD Biosciences	Cat#554714
BD APC BrDU Kit		BD Biosciences	Cat#552598
SuperScript™ III First-Strand Synthesis System		Invitrogen	Cat#18080051
Ce3D™ Tissue Clearing Kit		Biolegend	Cat#427701
**Oligonucleotides**			
qRT-PCR Primer Sequences		See method details	n/a
**Software**			
FlowJo 10.8.0			https://www.flowjo.com/
SeqGeq v 1.8.0			https://www.flowjo.com/solutions/seqgeq/downloads
Graphpad v 9.3.0			https://www.graphpad.com/
Leica Application Suite AF 2.6.3			https://www.leica-microsystems.com/
Imaris x64 v8.2.1			https://imaris.oxinst.com/imaris-viewer

Labscope v3.4.2		https://www.zeiss.com/microscopy/en/products/software/zeiss-labscope.html
ImageJ v1.54		https://imagej.net/ij/download.html

a

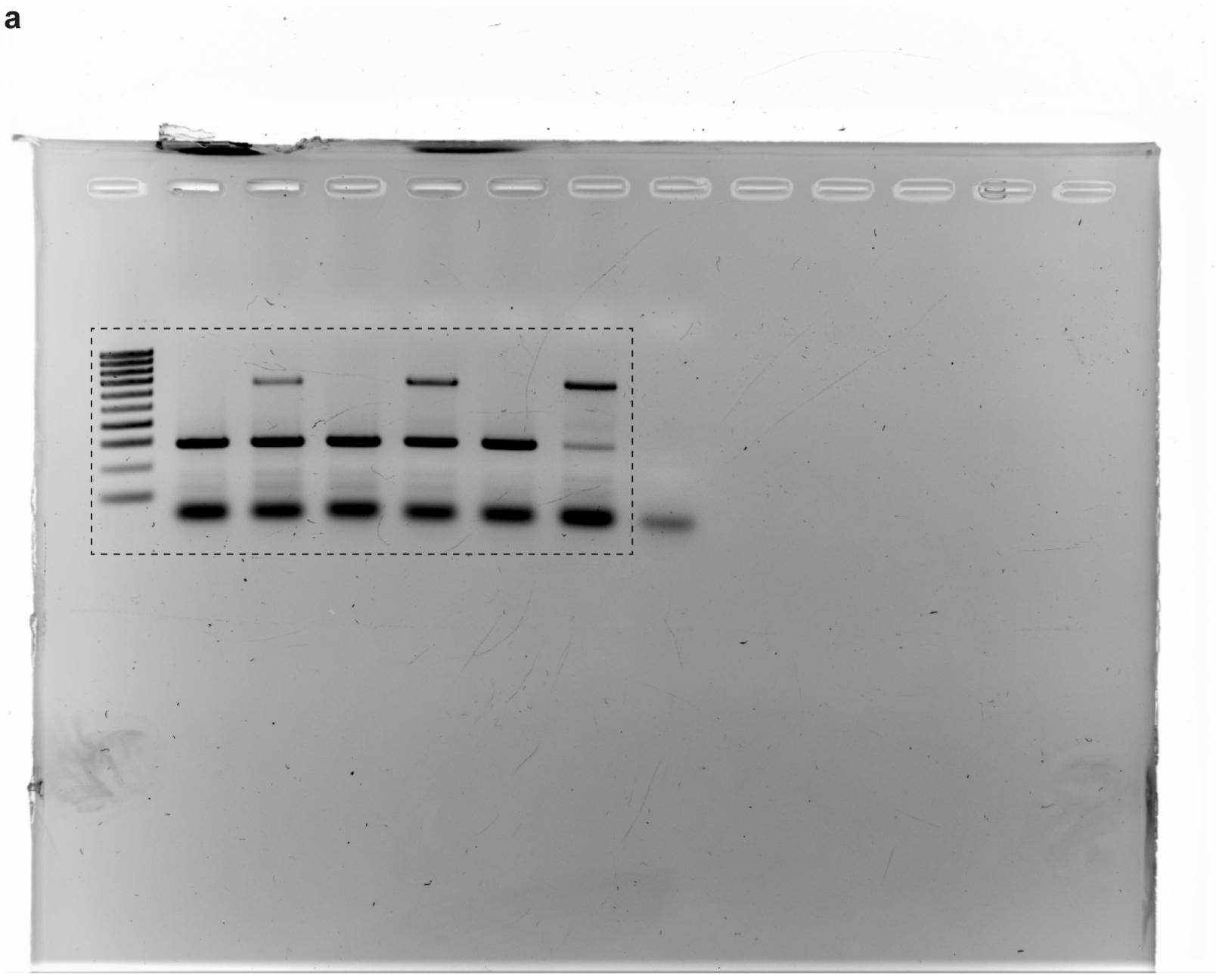

b

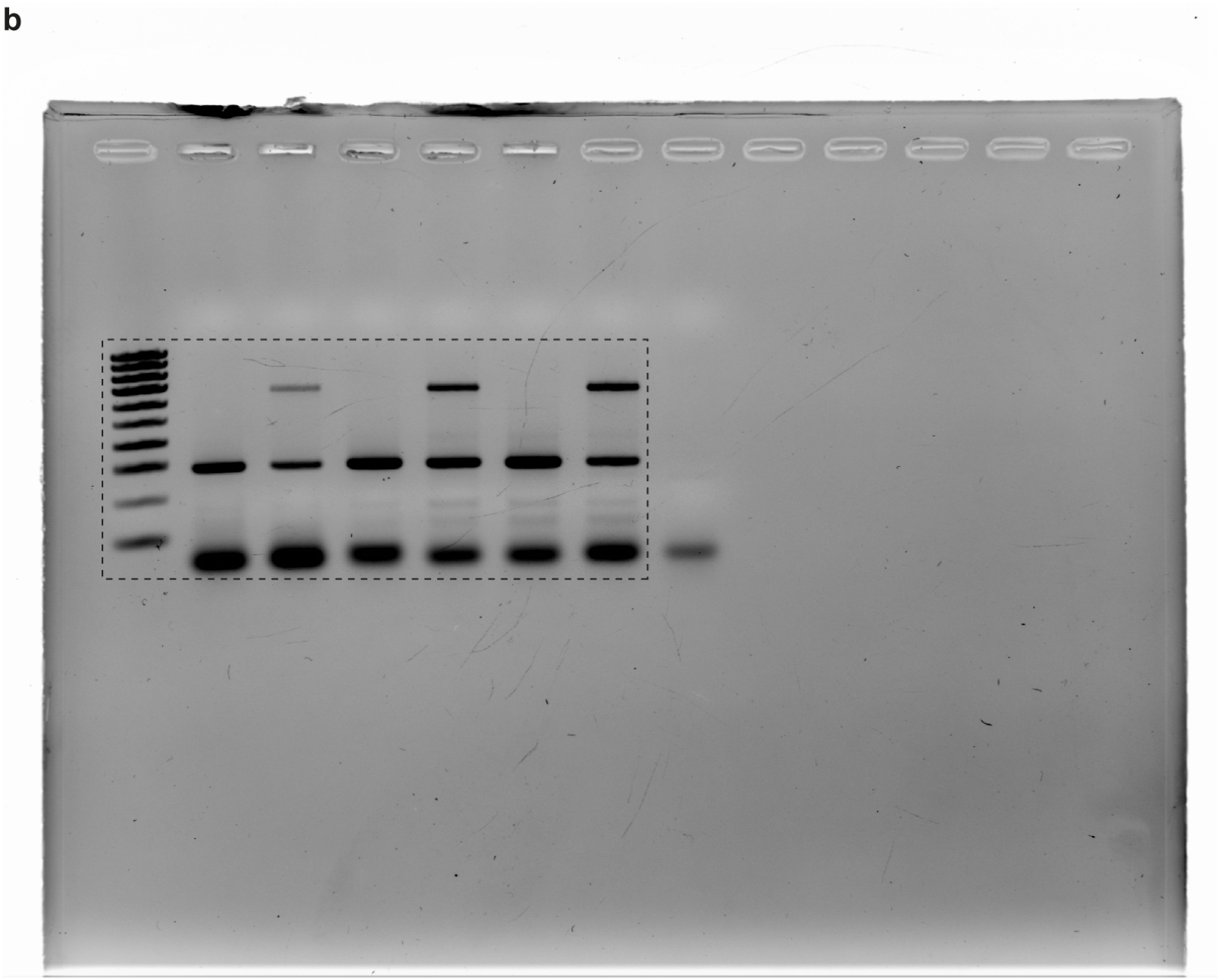

Uncropped images of Gel, related to Supplementary Fig. 1a, b.

PCR and gel-electrophoresis of sorted RPM, BMM, Ly6C^{hi} and Ly6C^{lo} monocytes from the Spl (**a**) and BM (**b**) showing the floxed (*Notch2$^{f/f}$*, 293 bp) and recombined alleles (*ΔNotch2*, 599 bp) in Ctrl or *N2^{ΔCx3cr1}* mice.
