# Supplementary material for: Canonical Notch2 signaling regulates development and maintenance of iron-recycling macrophages and iron homeostasis
Source: Nat Commun. 2026 Sep 23;17:10132. doi: 10.1038/s41467-026-77938-7 (PMC13601512; doi:10.1038/s41467-026-77938-7)
Supplement: Supplementary file 4 [file 41467_2026_77938_MOESM2_ESM.pdf]

Description of Additional Supplementary Files

Supplementary Data 1. Raw and filtered differential gene expression results (q < 0.05) comparing $N2^{\Delta Cx3cr1}$ or $Rbpj^{\Delta Cx3cr1}$ RPM over Ctrl.

Supplementary Data 2. Raw and filtered differential gene expression results (q < 0.05) comparing $N2^{\Delta Cx3cr1}$ or $Rbpj^{\Delta Cx3cr1}$ Ly6C$^{lo/int}$ monocytes over Ctrl.

Supplementary Data 3. Raw and filtered differential gene expression results (q < 0.05) comparing $N2^{\Delta Cx3cr1}$ or $Rbpj^{\Delta Cx3cr1}$ Ly6C^{hi} monocytes over Ctrl.

Supplementary Data 4. Enrichment analysis results from MSigDB Hallmark and Gene Ontogeny (GO) Biological Process gene sets comparing $N2^{\Delta Cx3cr1}$ RPM over Ctrl.

Supplementary Data 5. Enrichment analysis results from MSigDB Hallmark and Gene Ontogeny (GO) Biological Process gene sets comparing $N2^{\Delta Cx3cr1}$ Ly6C$^{lo/int}$ over Ctrl.

Supplementary Data 6. Enrichment analysis results from MSigDB Hallmark and Gene Ontogeny (GO) Biological Process gene sets comparing $Rbpj^{\Delta Cx3cr1}$ RPM over Ctrl.

Supplementary Data 7. Enrichment analysis results from MSigDB Hallmark and Gene Ontogeny (GO) Biological Process gene sets comparing $Rbpj^{\Delta Cx3cr1}$ Ly6C$^{lo/int}$ over Ctrl.

Supplementary Video 1: 3D Model depicting normal splenic microarchitecture in Ctrl mice. F4/80^{+} cells in red. CD169^{+} cells in green. Scale bar 50 μm.

Supplementary Video 2: 3D Model depicting impaired splenic microarchitecture in canonical Notch2 signaling-deficient mice. F4/80^{+} cells in red. CD169^{+} cells in green. Scale bar 50 μm.
